# Supplementary material for: A flavin-monooxygenase catalyzing oxepinone formation and the complete biosynthesis of vibralactone
Source: Nat Commun. 2023 Jun 10;14:3436. doi: 10.1038/s41467-023-39108-x (PMC10257657; doi:10.1038/s41467-023-39108-x)
Supplement: Supplementary file 1 — Supporting Information [file 41467_2023_39108_MOESM1_ESM.pdf]

Supplementary Information

A flavin-monooxygenase catalyzing oxepinone formation and the complete biosynthesis of vibralactone

Ke-Na Feng[1#], Yue Zhang[1,5#], Mingfang Zhang[2,5#], Yan-Long Yang[1,4], Ji-Kai Liu[3], Lifeng Pan[2*], Ying Zeng[1*]

[1] State Key Laboratory of Phytochemistry and Plant Resources in West China and Yunnan Key Laboratory of Natural Medicinal Chemistry, Kunming Institute of Botany, Chinese Academy of Sciences, Kunming 650201, China

[2] State Key Laboratory of Chemical Biology, Shanghai Institute of Organic Chemistry, University of Chinese Academy of Sciences, Chinese Academy of Sciences, Shanghai 200032, China

[3] School of Pharmaceutical Sciences, South-Central Minzu University, Wuhan 430074, China

[4] College of Chemistry and Chemical Engineering, Lanzhou University, Lanzhou 730000, China

[5] University of Chinese Academy of Sciences, Beijing 100049, China

[#] These authors contributed equally: Ke-Na Feng, Yue Zhang, Mingfang Zhang

**Corresponding authors*

Lifeng Pan, email: panlf@sioc.ac.cn; ORCID: 0000-0002-9229-6288

Ying Zeng, email: biochem@mail.kib.ac.cn; ORCID: 0000-0002-7581-6042

Supplementary Figures

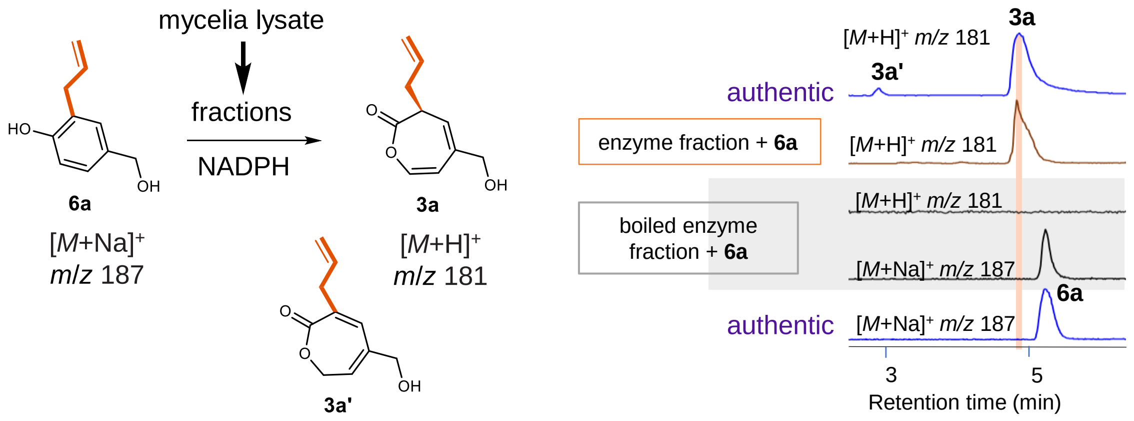

Supplementary Fig. 1. Specific assays for activity-guided fractionation of proteins with the VibO activity. The biosynthetic intermediate **6** may lead to production of **3** presumably by the catalysis of an oxygenase, which was subsequently identified and named VibO in this study. The metabolite **3** is obvious not only in the fungal *B. vibrans* culture broth, but also in mycelia lysates. Moreover, **3** may still exist in lysate fractions even after DEAE-Sepharose FF chromatography. Consequently, it seems unlikely to recognize the product **3** specifically attributed to the VibO activity in lysate fractions if normal **6** is used as substrate in in vitro enzyme assays during the activity-guided fractionation. Since an allyl C_3 mark is extremely rare in natural products from mushrooms, we previously synthesized **6a** (an analogue of **6**) that harbor an allyl rather than the prenyl moiety and fed to the *B. vibrans* fungal culture. Scale-up (15 L) feedings of **6a** (4.92 g) and systematic purification afforded **3a** (34 mg) and **1a** (18 mg). Incubation of **6a** with the cleared mycelia lysate led to accumulation of **3a**, as observed by LC-MS. Therefore, by using **6a** as a substrate and **3a** as a product standard, we can establish a highly specific assay for the VibO activity during the activity-guided fractionation.

Mycelia lysate fraction of 0.2 mL was incubated with **6a** (0.5 mM) and NADPH (0.5 mM) at 28 °C for 2 hours, then the reaction mixture was extracted with equal volume of ethyl acetate three times. The dried extract was dissolved in 50 μL of methanol and analyzed by LC-MS. Chromatographic separation of **6a**, **3a**, and **3a′** (an isomer of **3a**) was conducted with elution of 25% B over 4.8 min and 100% B over the next 3.2 min where A was H_2O and B was methanol. The mass spectrometer was run in positive ionization mode.

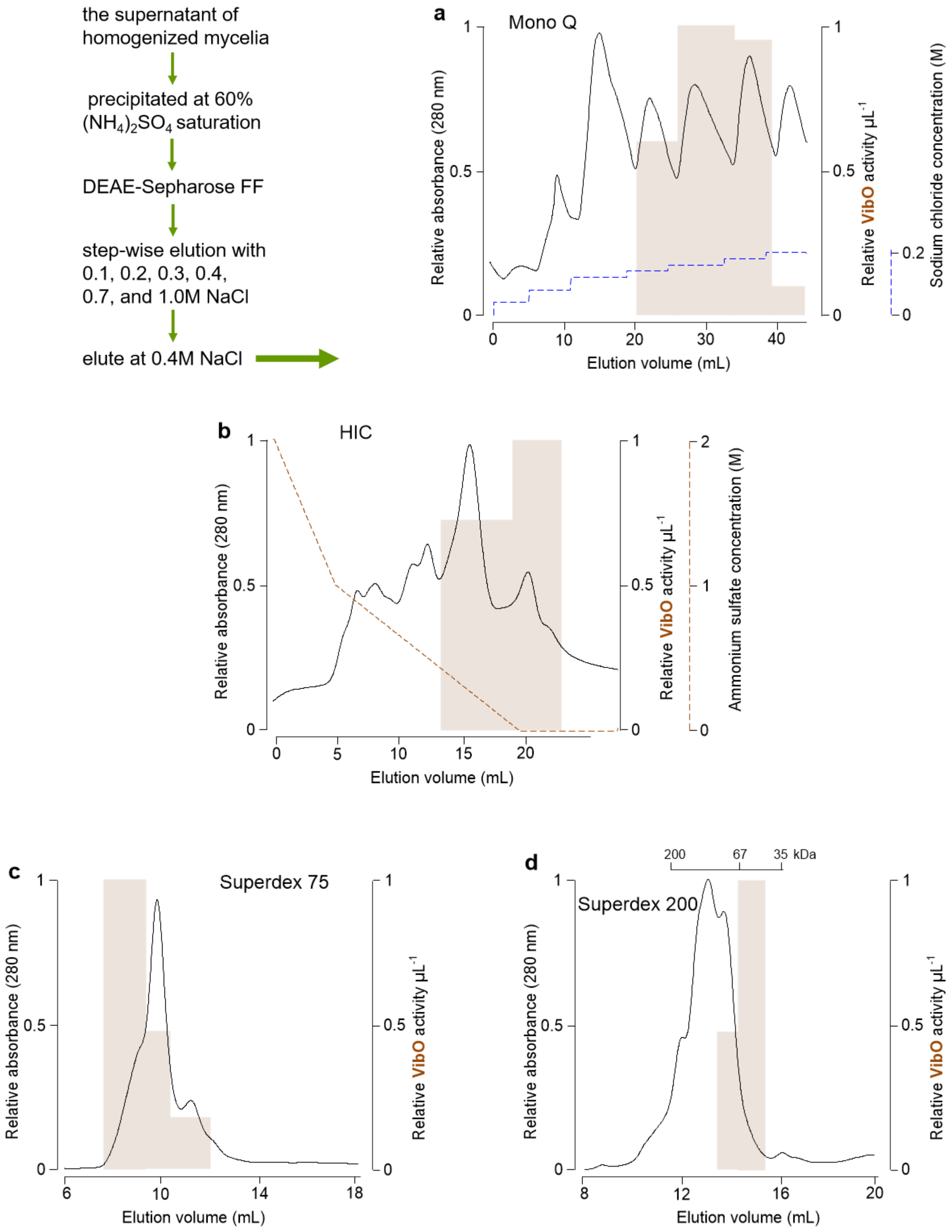

Supplementary Fig. 2. Activity-guided fractionation of proteins with the VibO activity. The enriched enzyme proteins were separated in sequential order by (**a**) ion-exchange chromatography with a Mono Q column, (**b**) hydrophobic interaction chromatography with a Resource PHE column, and size-exclusion chromatography with (**c**) Superdex 75 10/300 GL and (**d**) Superdex 200 Increase 10/300 GL columns. See Supplementary Method 1 for details. Fractions having the VibO activity were shaded in gray columns. Assays were repeated twice with similar results.

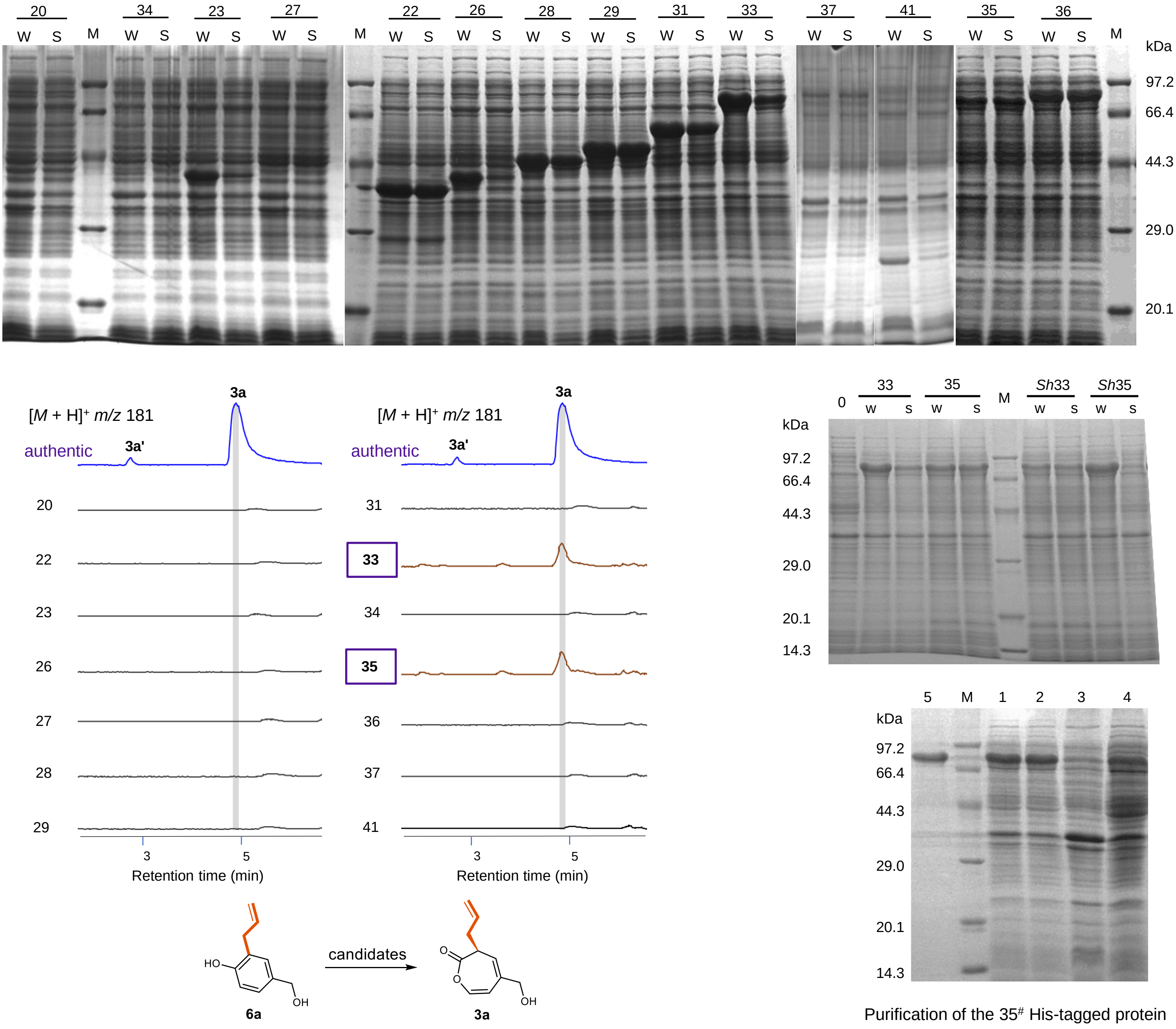

Supplementary Fig. 3. SDS-PAGE for VibO candidates and their reactions with 6a **analyzed by LC-MS**. Proteomic analyses identified 69 proteins common to all the seven active fractions, among which 19 candidates were selected for cDNA clones but five candidates (in gray numbers, Source Data and Supplementary Table 2) failed in full-length cloning. Candidates were cloned from mRNAs of cultured mycelia of *B. vibrans*, expressed via pET28a(+) in *Escherichia coli* BL21(DE3), and induced with 0.1 mM IPTG (isopropyl β-D-thiogalactoside) at 16 °C for 24 h. Out of 14 candidates, only two (33# and 35#, sharing 99% amino acid identity) can produce **3a** from **6a** in the in vitro assays using 0.2 mL cleared *E. coli* lysate with 0.5 mM **6a** and 0.2 mM NADPH, incubated at 28 °C for 2 hours. Chromatographic separation of **6a** and **3a** was the same as described in Supplementary Fig. 1.

The SDS-PAGE experiments were repeated twice independently with similar results and the original gel photographs were supplied at the end of this file. M, protein size marker; w, whole proteins; s, soluble proteins; 0, control with empty vector. For purification of the His6-tagged recombinant candidate 35#: 1, whole proteins; 2, soluble proteins; 3, unbinding proteins; 4, washing; 5, eluate of the purified protein.

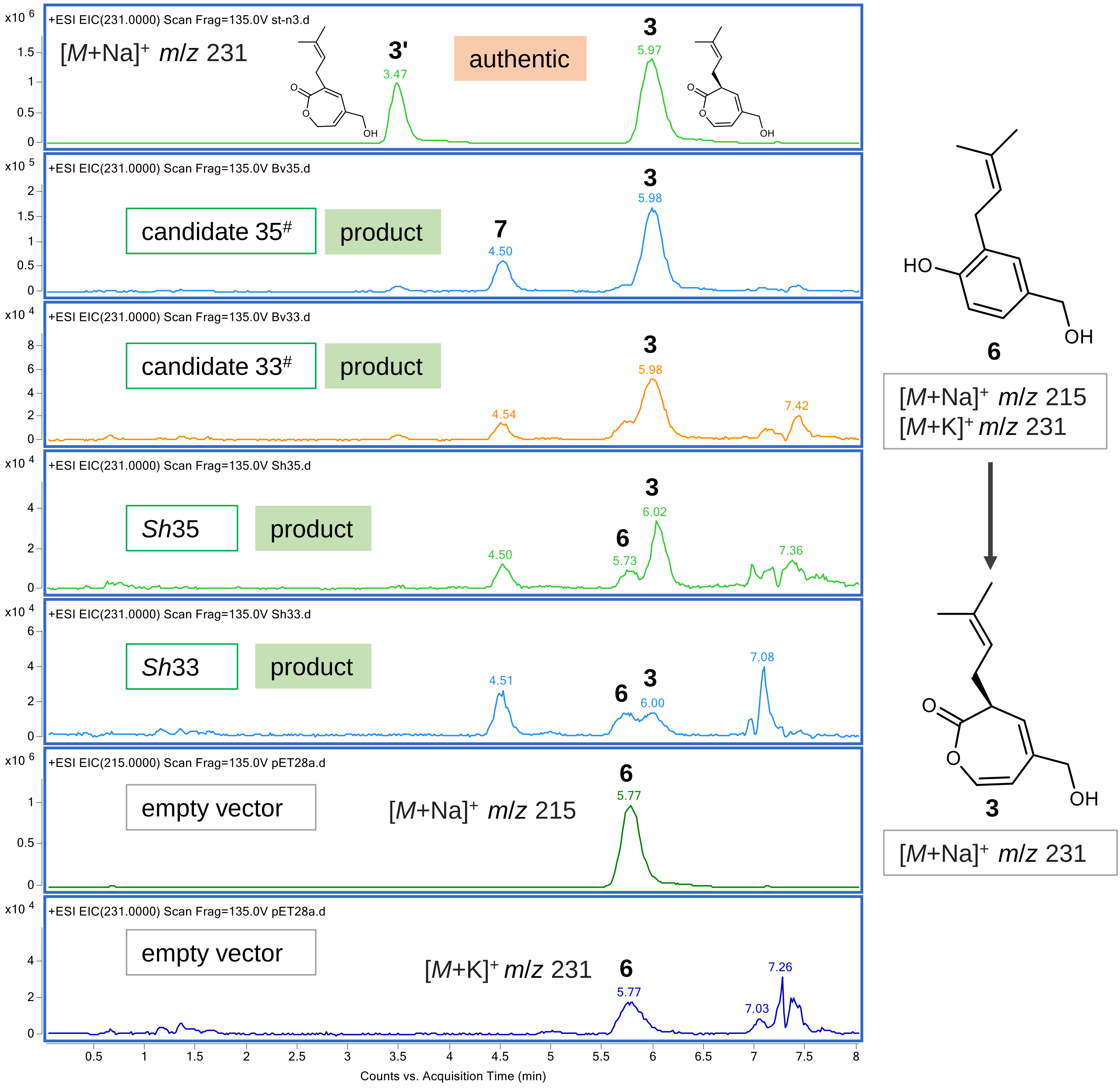

Supplementary Fig. 4. LC-MS analysis of candidates 33# and 35# incubating with 6 **as substrate.** The cleared *E. coli* lysates as crude enzymes were incubated with **6** (0.5 mM) and NADPH (0.2 mM) at 28 °C for 2 hours. The *E. coli* strain containing pET28a(+) without target sequences (denoted by “empty vector”) was used as control. The candidate 35# was designated as VibO.

*Sh*33 and *Sh*35, homologues of VibO, were cloned from mRNA of the cultured *Stereum hirsutum* using primers listed in Supplementary Table 2 and expressed in *E. coli* as described in Supplementary Fig. 3.

The chromatographic separation was performed with elution of 43% B over 4.9 min and 100% B over the next 3.1 min where A was H_2O and B was methanol. The mass spectrometer was run in positive ionization mode. The peak at 4.5 min was subsequently confirmed as **7**, see Supplementary Fig. 10.

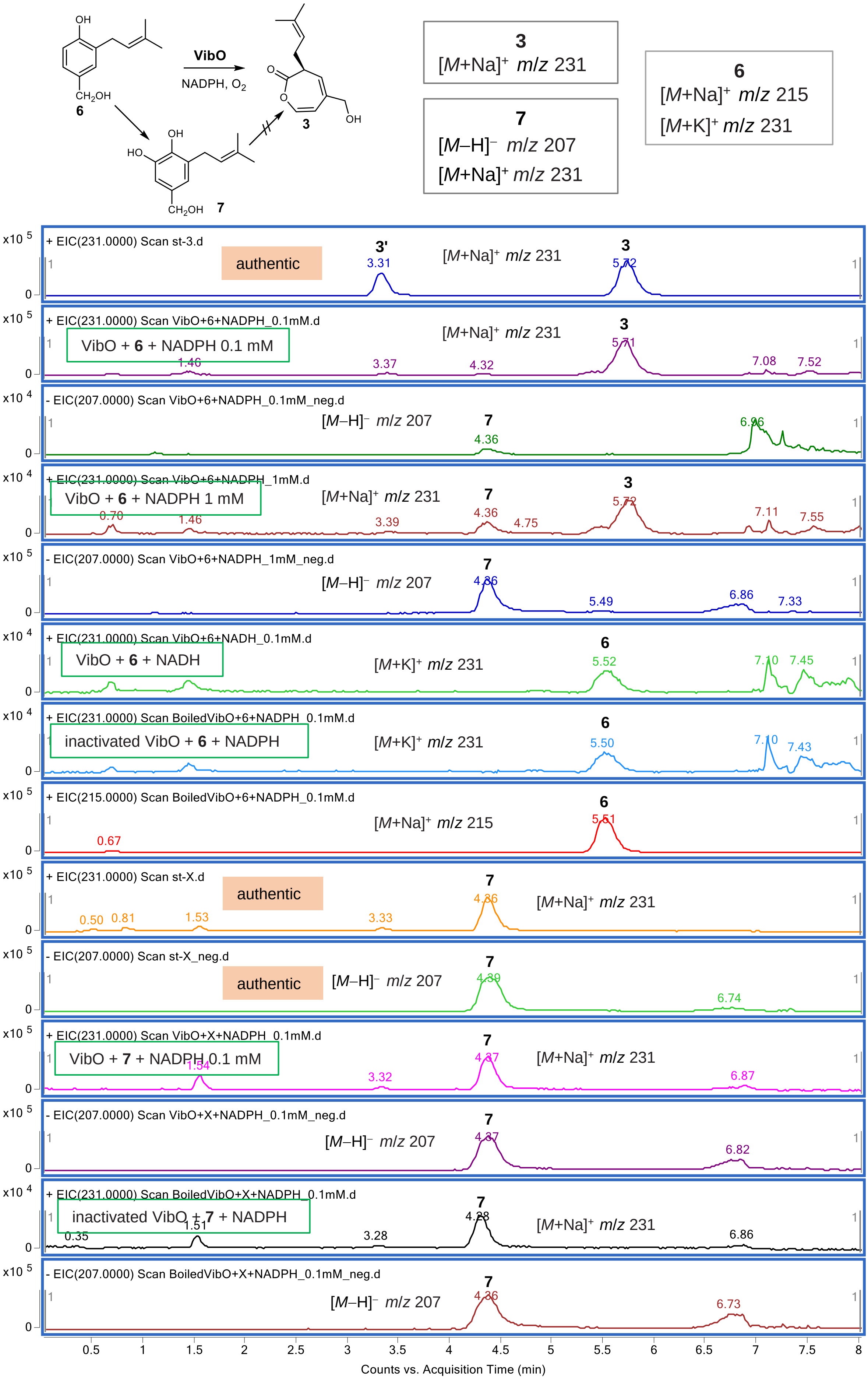

Supplementary Fig. 5. The original LC-MS data for Figure 2a.

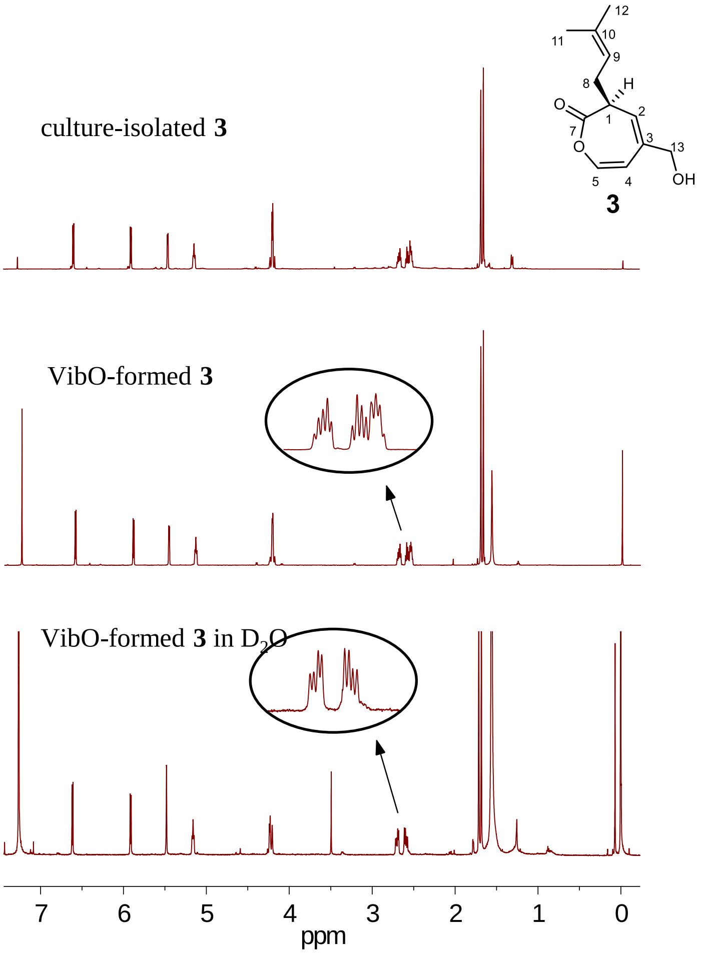

	δ_H (*J* in Hz)		
	culture-isolated **3**	VibO-formed **3**	VibO-formed **3** in D_2O
C1	2.53(1H, m)	2.55(1H, m)	
C2	5.46(1H, d, 4.6)	5.48(1H, d, 4.7)	5.48(1H, s)
C4	5.90(1H, d, 6.9)	5.91(1H, d, 6.9)	5.91(1H, d, 6.8)
C5	6.59(1H, d, 6.9)	6.62(1H, d, 6.9)	6.62(1H, d, 6.8)
C8	2.67(1H, m)	2.70(1H, m)	2.70(1H, m)
	2.58(1H, m)	2.60(1H, m)	2.60(1H, m)
C9	5.14(1H, m)	5.16(1H, m)	5.16(1H, m)
C11	1.67(3H, s)	1.68(3H, s)	1.68(3H, s)
C12	1.70(3H, s)	1.71(3H, s)	1.71(3H, s)
C13	4.22(1H, d, 14.1)	4.25(1H, d, 13.9)	4.25(1H, d, 13.8)
	4.19(1H, d, 13.3)	4.22(1H, d, 13.5)	4.22(1H, d, 14.4)

Supplementary Fig. 6. ^{1}H-NMR data of the product 3 **formed by VibO in normal and in D_2O system.**

The authentic **3** was isolated from *B. vibrans* mycelial culture broth. In one case[1], 46 mg **3** and 1.8 g **1** were obtained from 20 L culture for 21 days at 25 °C. In another case[2], only 65 mg **1** was isolated from 12 L culture.

The VibO-formed **3** (~12 mg) was extracted and purified from a whole-cell transformation (in vivo) for 24 h at 25 °C by feeding 90 mg of **6** to the *E. coli* cell culture (2.5 L) expressing VibO after induction with IPTG (0.1 mM) for 4 h. By the same way of whole-cell transformation, D-labelled **3** was obtained in D_2O system.

See Supplementary Figure 7 for the original ^{1}H-NMR spectra (600 MHz, $CDCl_3$).

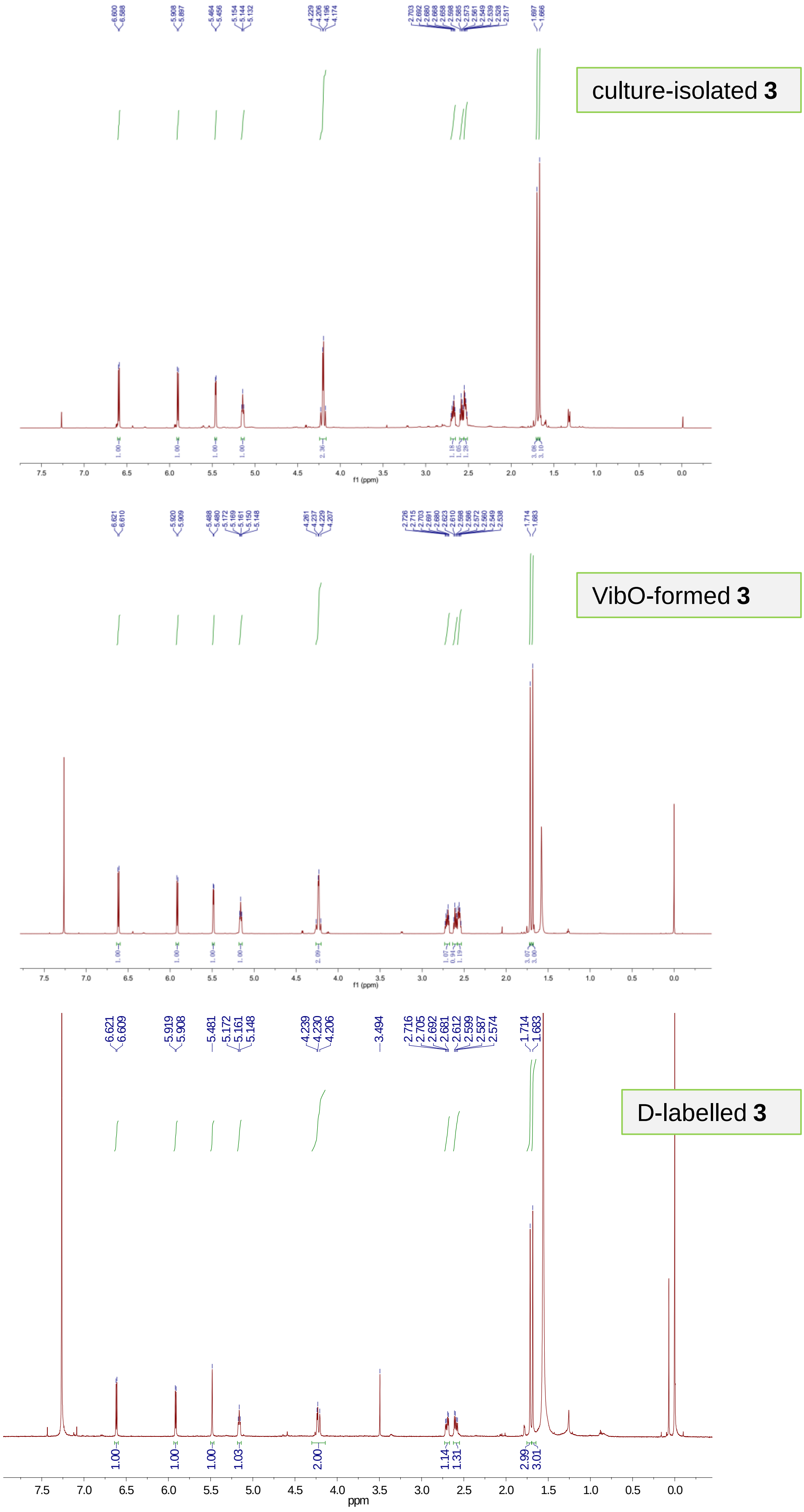

Supplementary Fig. 7. The original ^{1}H-NMR spectra (600 MHz, $CDCl_3$) in Supplementary Fig. 6.

culture-isolated **3**

n	Average	Std.Dev.	% RSD	Maximum	Minimum
5	23.80	0.97	4.07	25.25	22.92

S.No	Sample ID	Time	Result	Scale	OR °Arc	WLG.nm	Lg.mm	Conc.g/100ml	Temp.
1	FKN-3	03:36:27 PM	24.25	SR	0.0291	589	100.00	0.120	22.2
2	FKN-3	03:36:35 PM	23.00	SR	0.0276	589	100.00	0.120	22.2
3	FKN-3	03:36:44 PM	25.25	SR	0.0303	589	100.00	0.120	22.2
4	FKN-3	03:36:52 PM	22.92	SR	0.0275	589	100.00	0.120	22.2
5	FKN-3	03:37:00 PM	23.58	SR	0.0283	589	100.00	0.120	22.2

VibO-formed **3**

n	Average	Std.Dev.	% RSD	Maximum	Minimum
5	23.13	1.46	6.31	24.83	21.17

S.No	Sample ID	Time	Result	Scale	OR °Arc	WLG.nm	Lg.mm	Conc.g/100ml	Temp.
1	FKN-VibO-3	05:55:45 PM	21.17	SR	0.0254	589	100.00	0.120	22.5
2	FKN-VibO-3	05:55:54 PM	22.17	SR	0.0266	589	100.00	0.120	22.5
3	FKN-VibO-3	05:56:02 PM	23.58	SR	0.0283	589	100.00	0.120	22.4
4	FKN-VibO-3	05:56:10 PM	23.92	SR	0.0287	589	100.00	0.120	22.4
5	FKN-VibO-3	05:56:18 PM	24.83	SR	0.0298	589	100.00	0.120	22.4

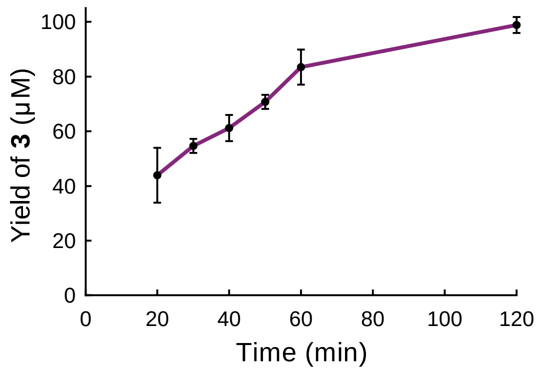

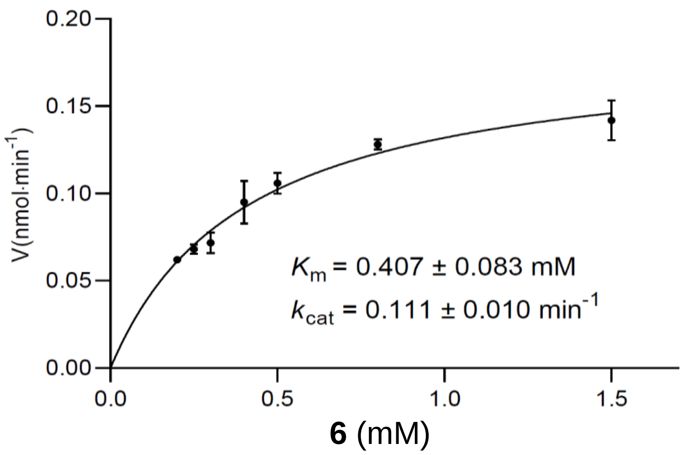

Supplementary Fig. 8. Optical rotations of **3** were measured on Autopol VI (Rudolph Research Analytical, USA) in $CHCl_3$. **The time-course production** of **3** from in vitro reactions was calculated by its standard curve; the reaction mixture (0.1 mL) contained 0.5 mM **6** (8.1 μg), 0.1 mM NADPH, and 17 μM VibO, incubating for 10~120 min at 28 °C; this in vitro reaction for 2 h was estimated to give ~2 μg **3**. **Kinetic parameters** for VibO with **6**; error bars represent standard deviations of velocity calculated from three independent replicates; Michaelis-Menten plots were constructed by Graphpad Prism 9. Source data are provided as a Source Data file.

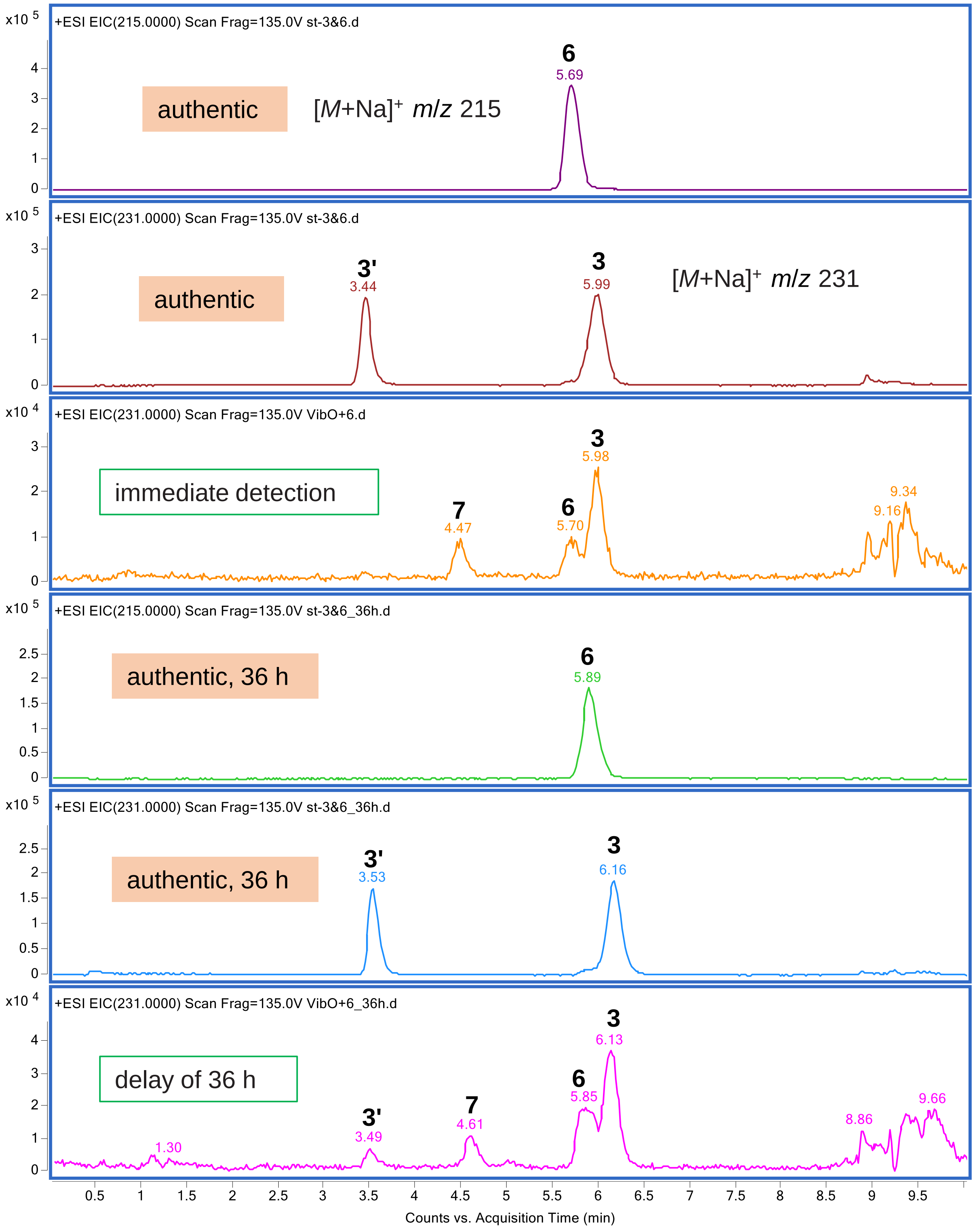

Supplementary Fig. 9. Verification of 3 **as the direct product of VibO incubated with** 6 **as substrate.** The reaction mixture was analyzed immediately after incubation without extraction and drying overnights, in which **3** was significantly observed but **3′** was barely detected. The remaining mixture was extracted and dried for 36 h prior to detection. In the 36 h-sample a small amount of **3′** was detected by LC-MS but **3** was still the majority. Authentic standards were analyzed in parallel each time. The chromatographic separation was performed with elution of 43% B over 6.9 min and 100% B over the next 3.1 min where A was H_2O and B was methanol.

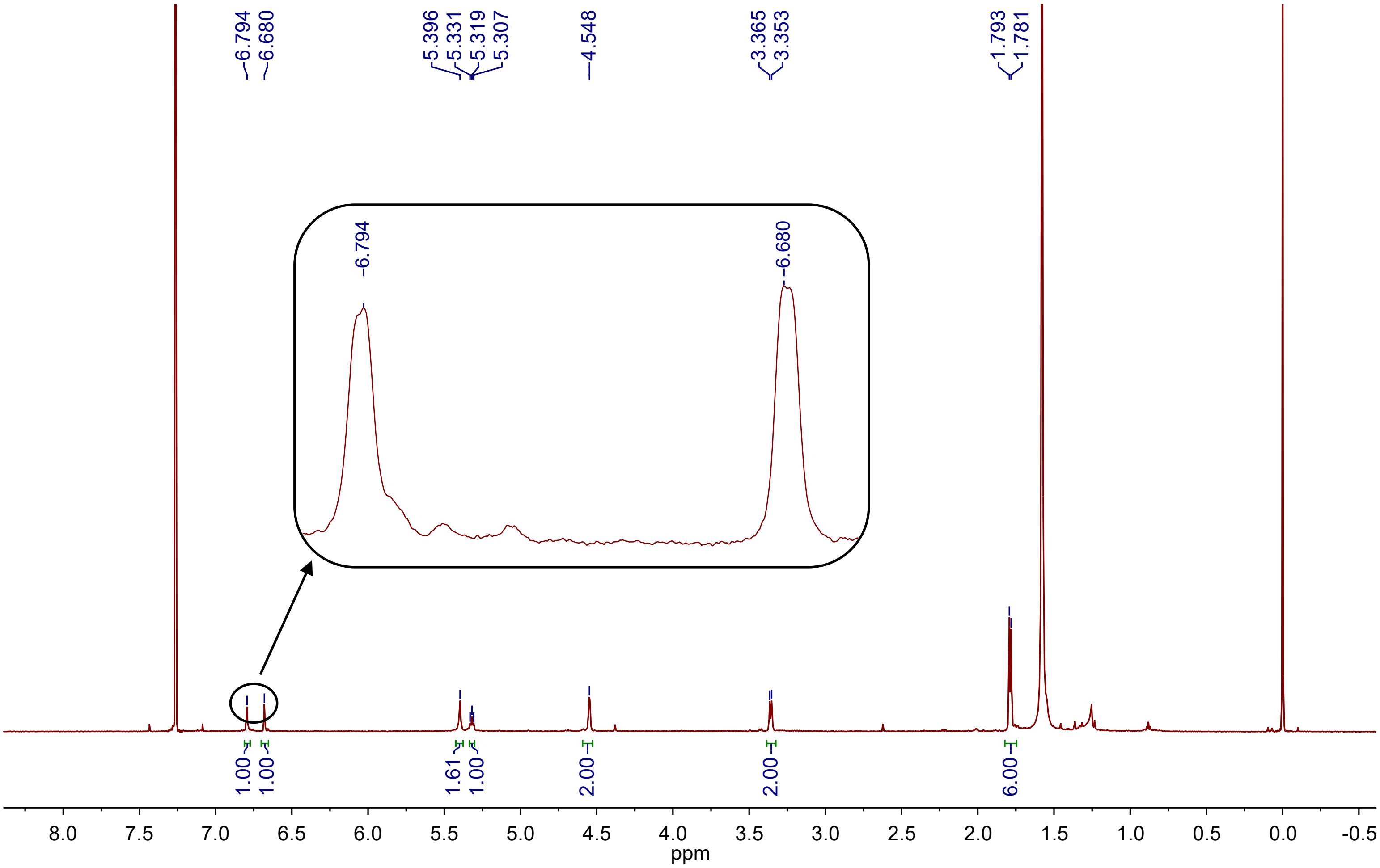

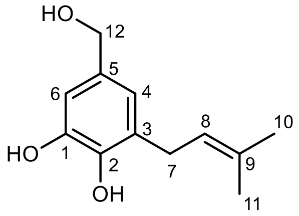

7

	δ_H ($CHCl_3$, 600 MHz)<br>**This study**	δ_H ($CHCl_3$, 270 MHz)<br>Reported
C4	6.68 (1H, s)	6.67 (1H, d)
C6	6.79 (1H, s)	6.78 (1H, d)
C7	3.36 (2H, d, 7.2)	3.35 (2H, d)
C8	5.32 (1H, t, 7.2)	5.31 (1H, m)
C10<br>C11	1.79 (6H, d, 6.8)	1.78 (6H, m)
C12	4.55 (2H, s)	4.54 (2H, s)
OH	5.40 (s)	5.5 (s)

Supplementary Fig. 10. ^{1}H-NMR spectral data for the compound 7. Since isolation of the side product was unsuccessful due to its very low production in the in vivo *E. coli* whole-cell transformation (Supplemental figure 14), we tried the in vitro reaction instead. The crude enzymes were prepared from cleared lysates of the induced *E. coli* cells (15 L) and incubated with 70 mg **6** for 4 h at 28 °C. Subsequent extraction and purification yielded ~0.5 mg of the product for ^{1}H-NMR analysis. The spectral data are consistent with those of the known compound **7** which was originally isolated from the fungal culture broth of *Verticillium biguttatum*[3].

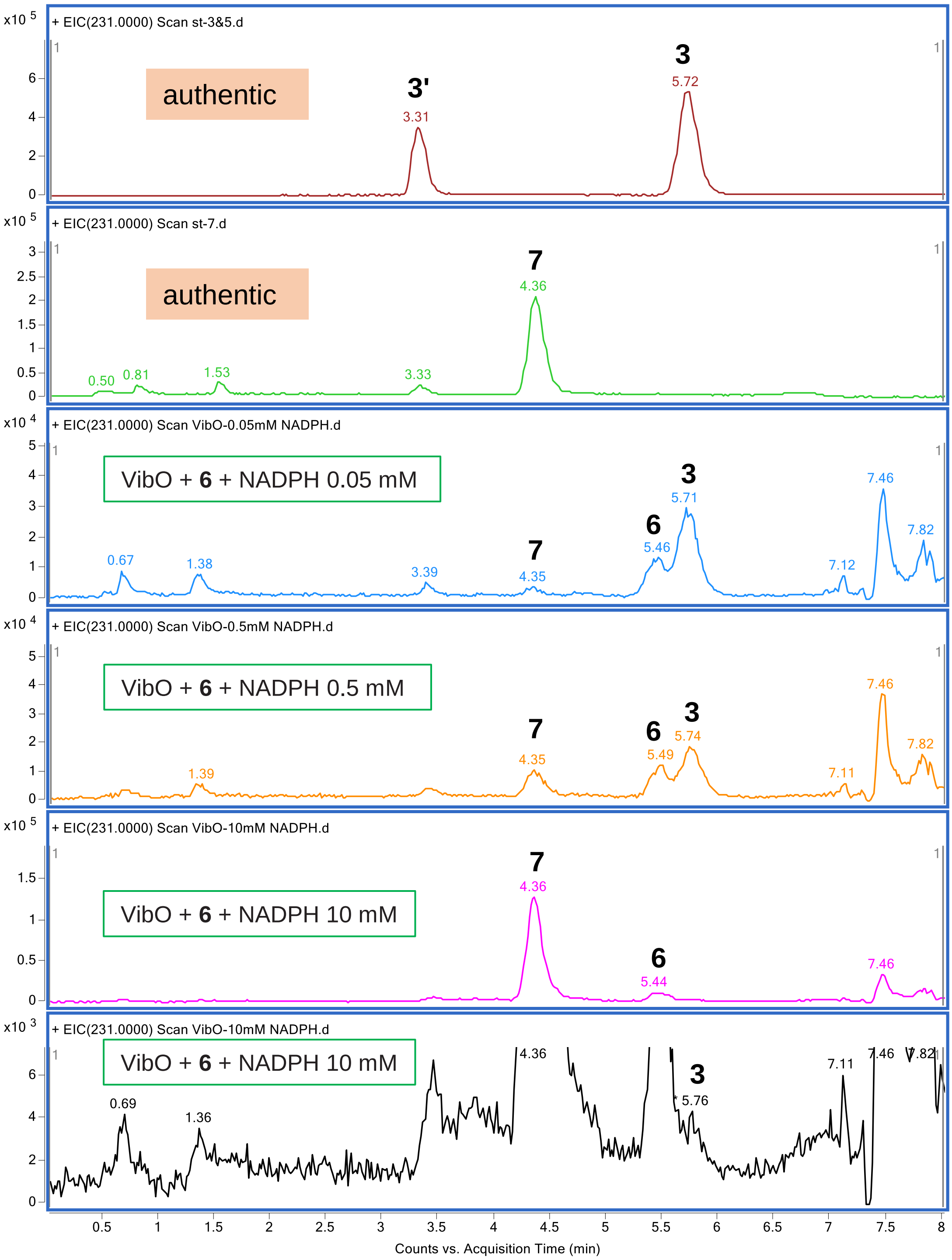

Supplementary Fig. 11. LC-MS data for NADPH dependence. The chromatographic separation was the same as described in Supplementary Fig. 4.

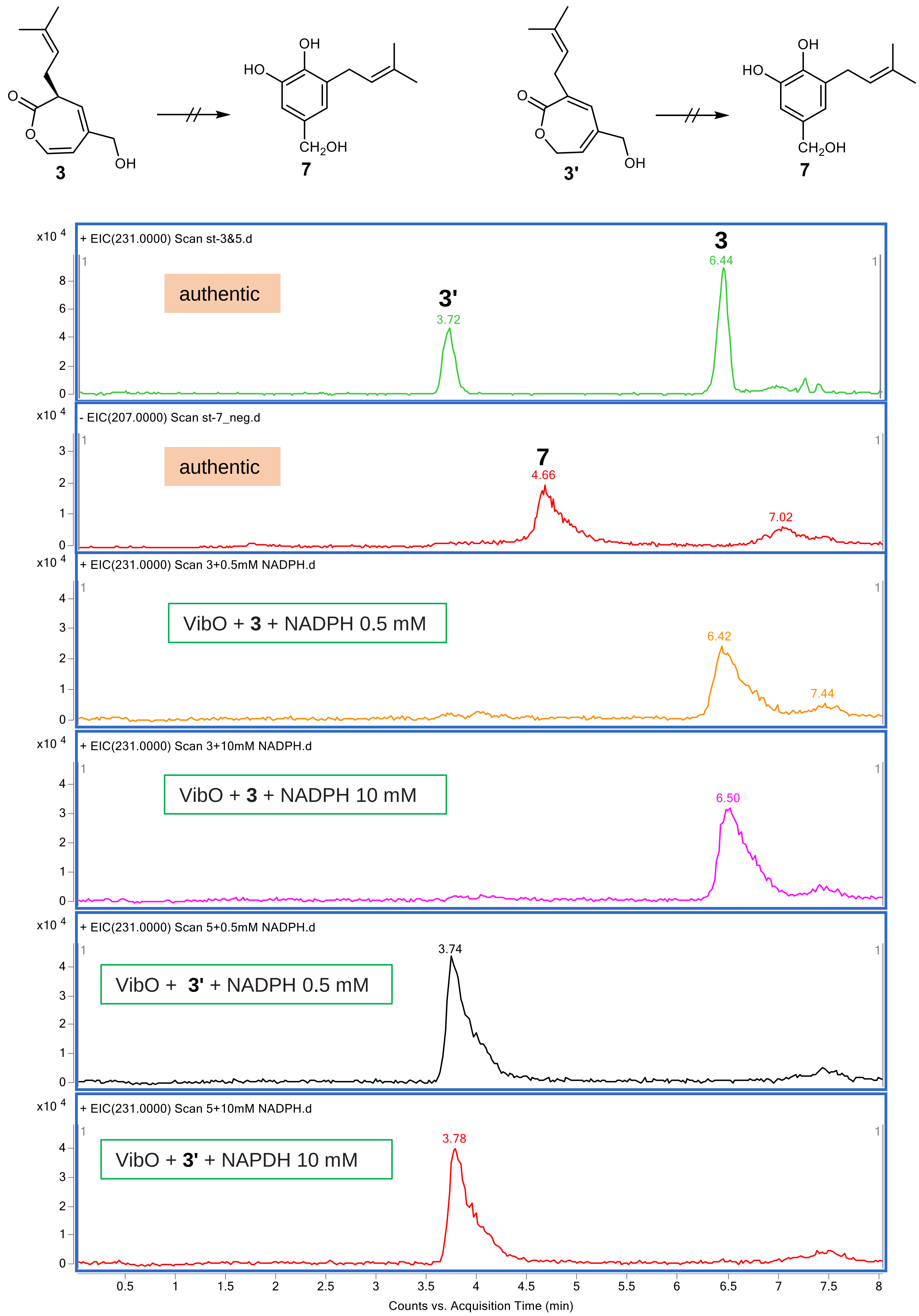

Supplementary Fig. 12. LC-MS analysis for incubation with 3 **or** 3′ **as substrate.** The reaction mixtures were kept at 28 °C for 2 hours. The chromatographic separation was the same as described in Supplementary Fig. 4.

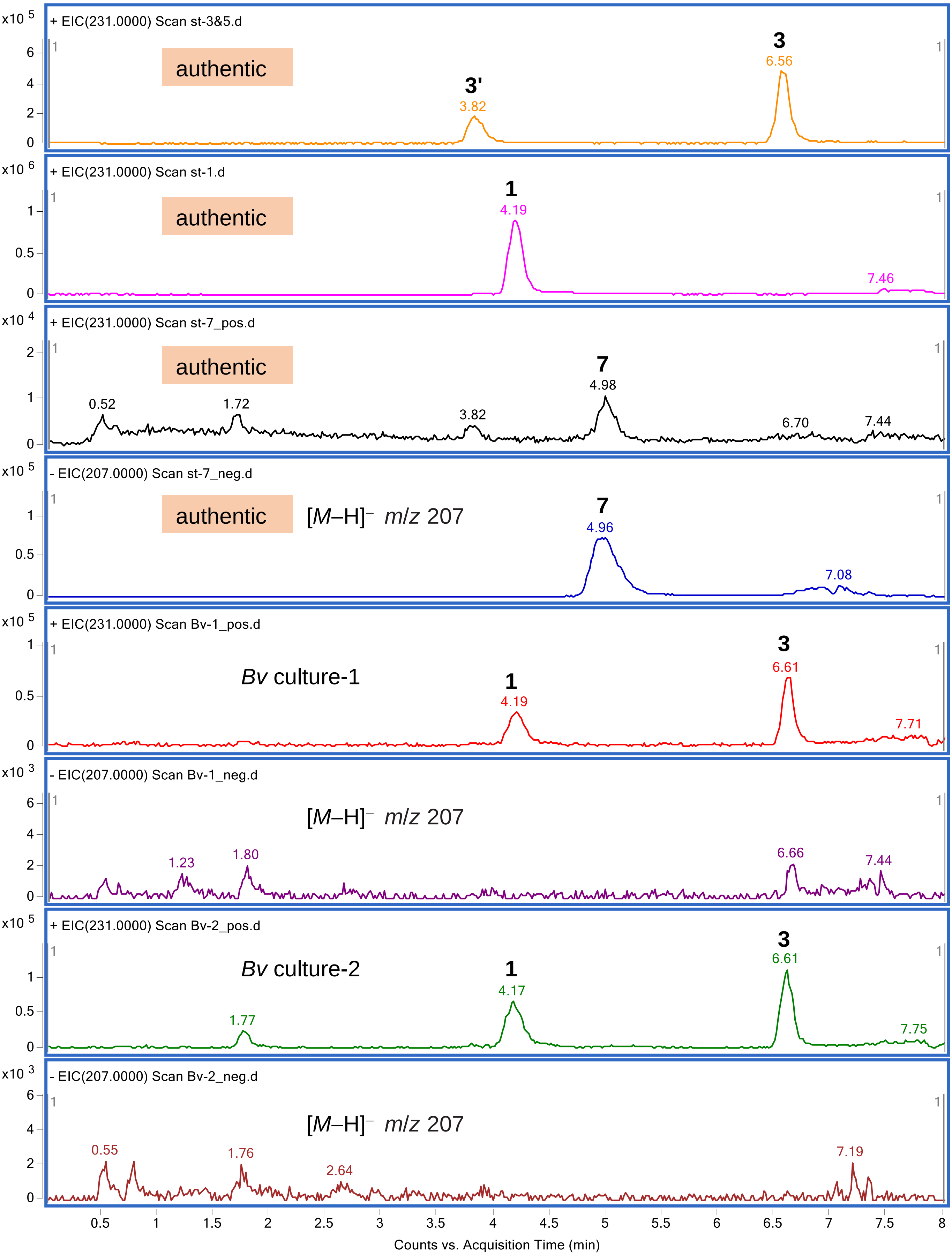

Supplementary Fig. 13. No trace of the compound 7 **was observed in the *B. vibrans* fungal culture broth**. The chromatographic separation was the same as described in Supplementary Fig. 4.

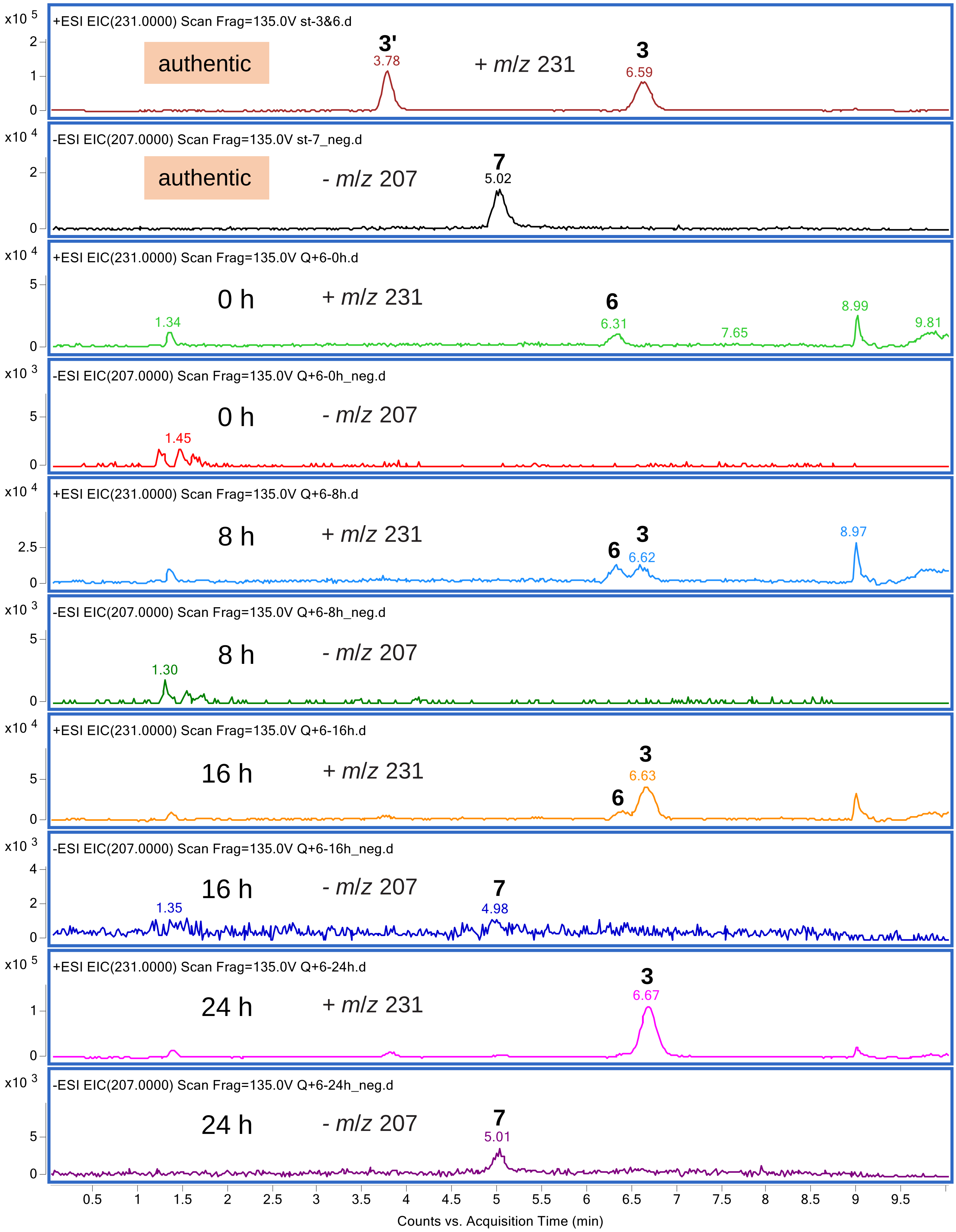

Supplementary Fig. 14. Time-course analysis of 3 **and** 7 **produced in *E. coli* whole-cell transformation**. After the *E. coli* cells expressing VibO were induced with IPTG 0.1 mM for 2 h at 25 °C, 0.5 mM **6** was added to the culture, immediately taking 0.2 mL of broth as control (0 h). The bioconversion was kept at 25 °C and detected at 8 h, 16 h, and 24 h. Samples (0.2 mL broth) were extracted with equal volume of ethyl acetate three times. The dried extract was dissolved in 50 μL of methanol and analyzed by LC-MS. The chromatographic separation was the same as described in Supplementary Fig. 9. The EIC trace of +*m/z* 231 covers both of **3** $[M+Na]^+$ and **6** $[M+K]^+$; the main ion of **6** is $[M+Na]^+$ *m/z* 215 (Supplementary Fig. 9).

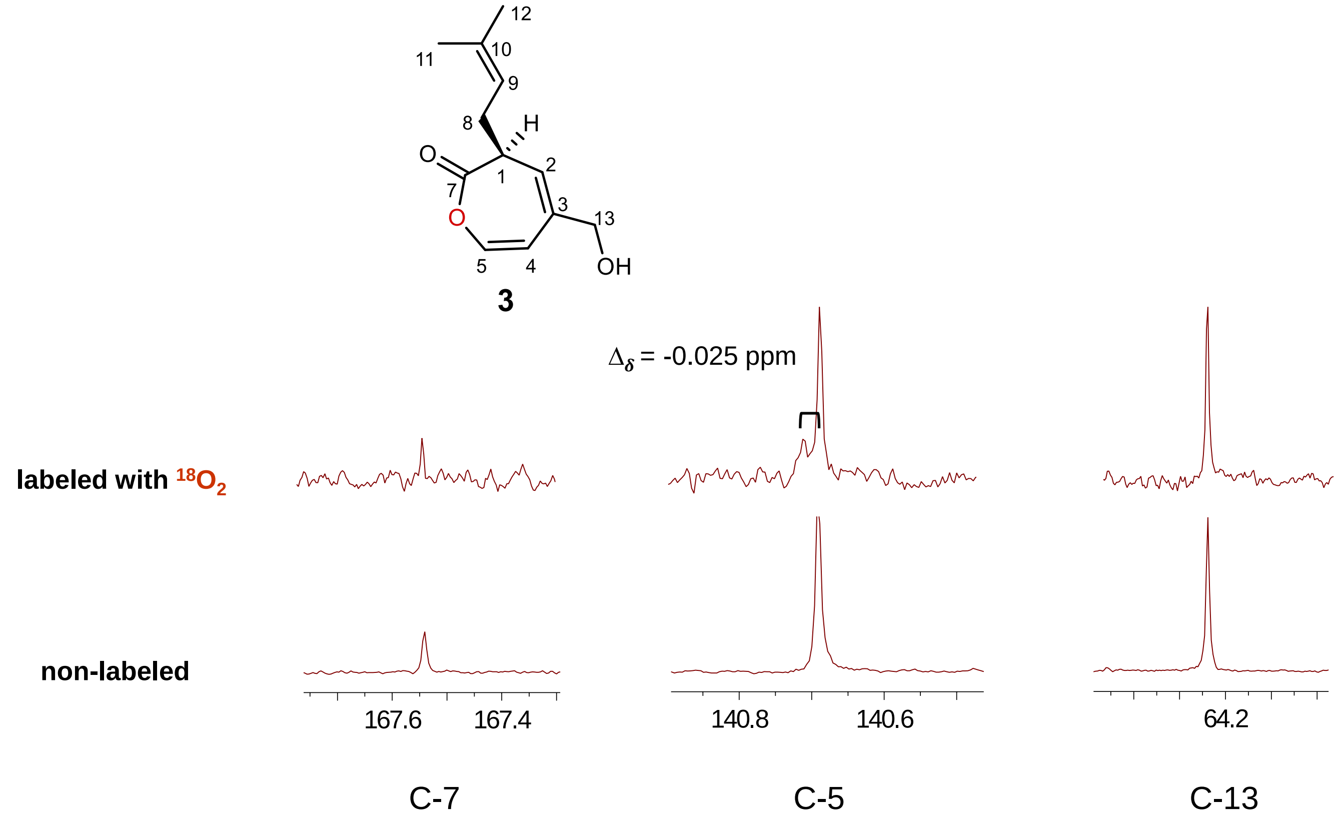

Supplementary Fig. 15. Partial ^{13}C-NMR spectra of 3 **labeled with $^{18}O_2$.** The reaction was carried out in a round bottomed flask containing 100 mL crude enzyme. After purge with nitrogen for 30 min to thoroughly remove the atmospheric O_2, $^{18}O_2$ (98% labeled) was introduced into the flask from the compressed gas cylinder via a syringe needle. Then, 20 mg **6** was added to the flask. After incubation at 28°C for 2 h, approximately 1.7 mg of **3** labeled with $^{18}O_2$ was extracted and purified from the reaction mixture. The original ^{13}C-NMR spectra were provided in Supplementary Fig. 16.

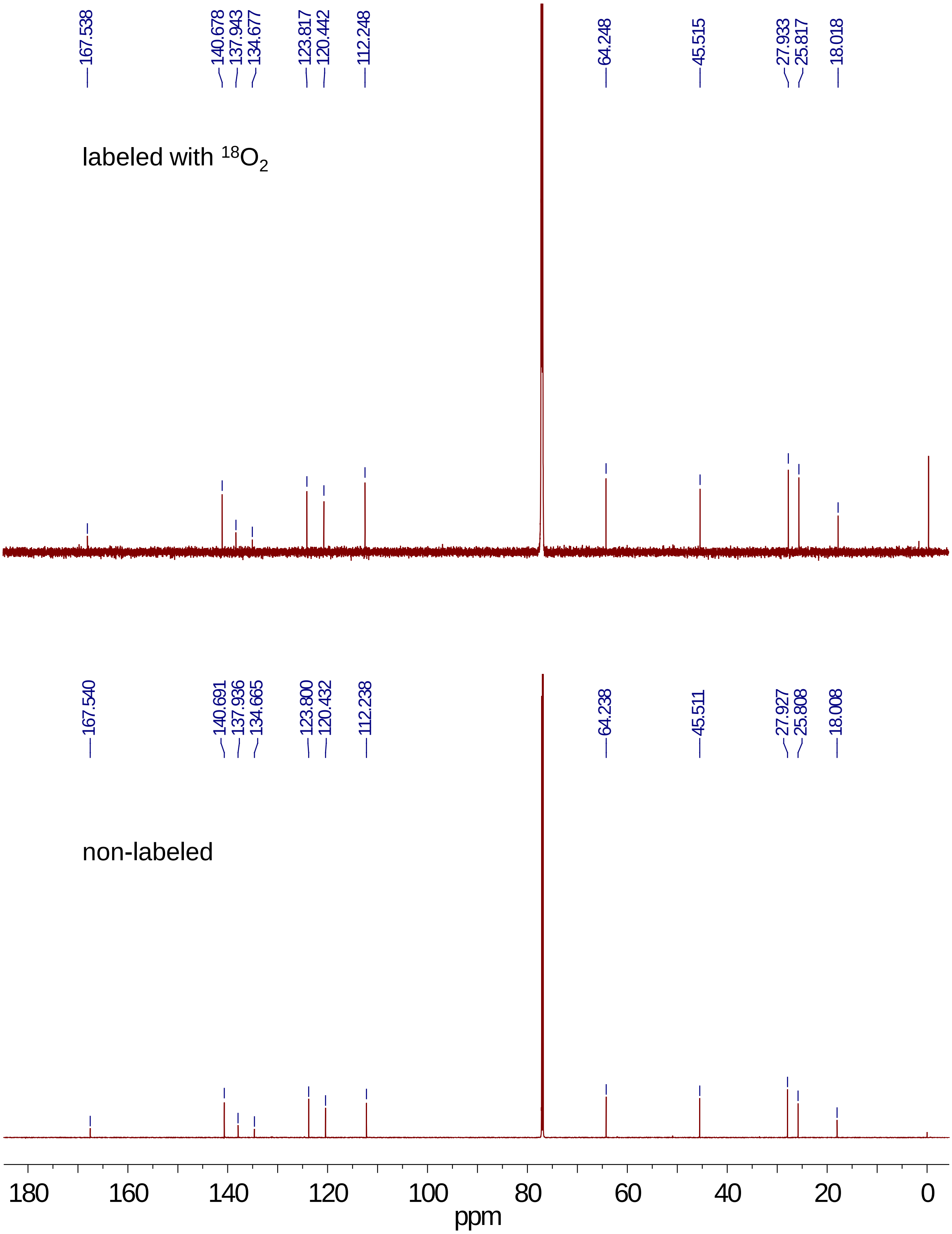

Supplementary Fig. 16. ^{13}**C NMR spectrum (200 MHz,** $CDCl_3$**) of** 3 **labeled with** $^{18}O_2$.

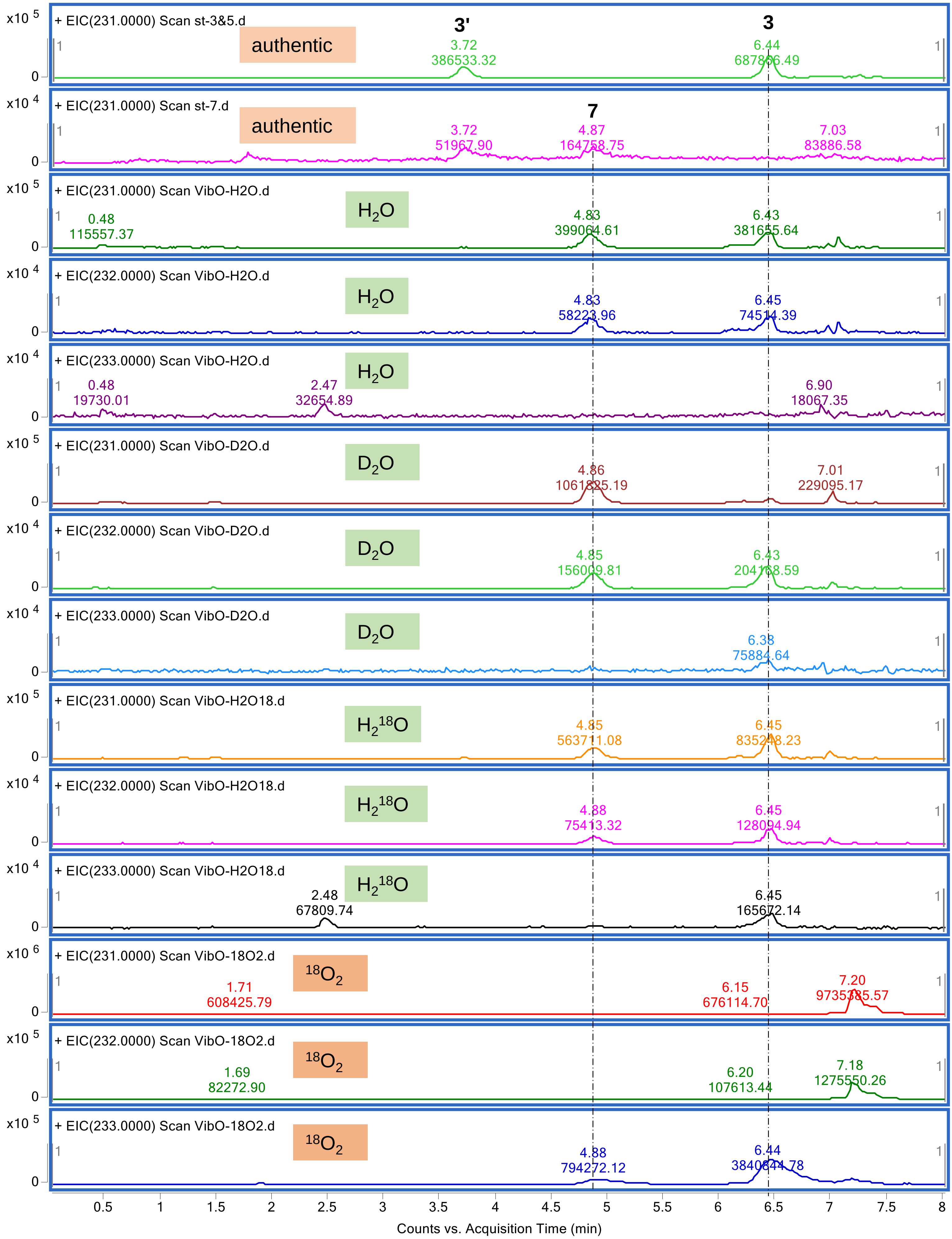

Supplementary Fig. 17. The original LC-MS data for labelling experiments. Reactions of VibO with **6** were conducted under $^{18}O_2$/ H_2O, O_2 / $H_2^{18}O$, O_2 / D_2O, and O_2 / H_2O systems. The chromatographic separation was the same as described in Supplementary Fig. 4.

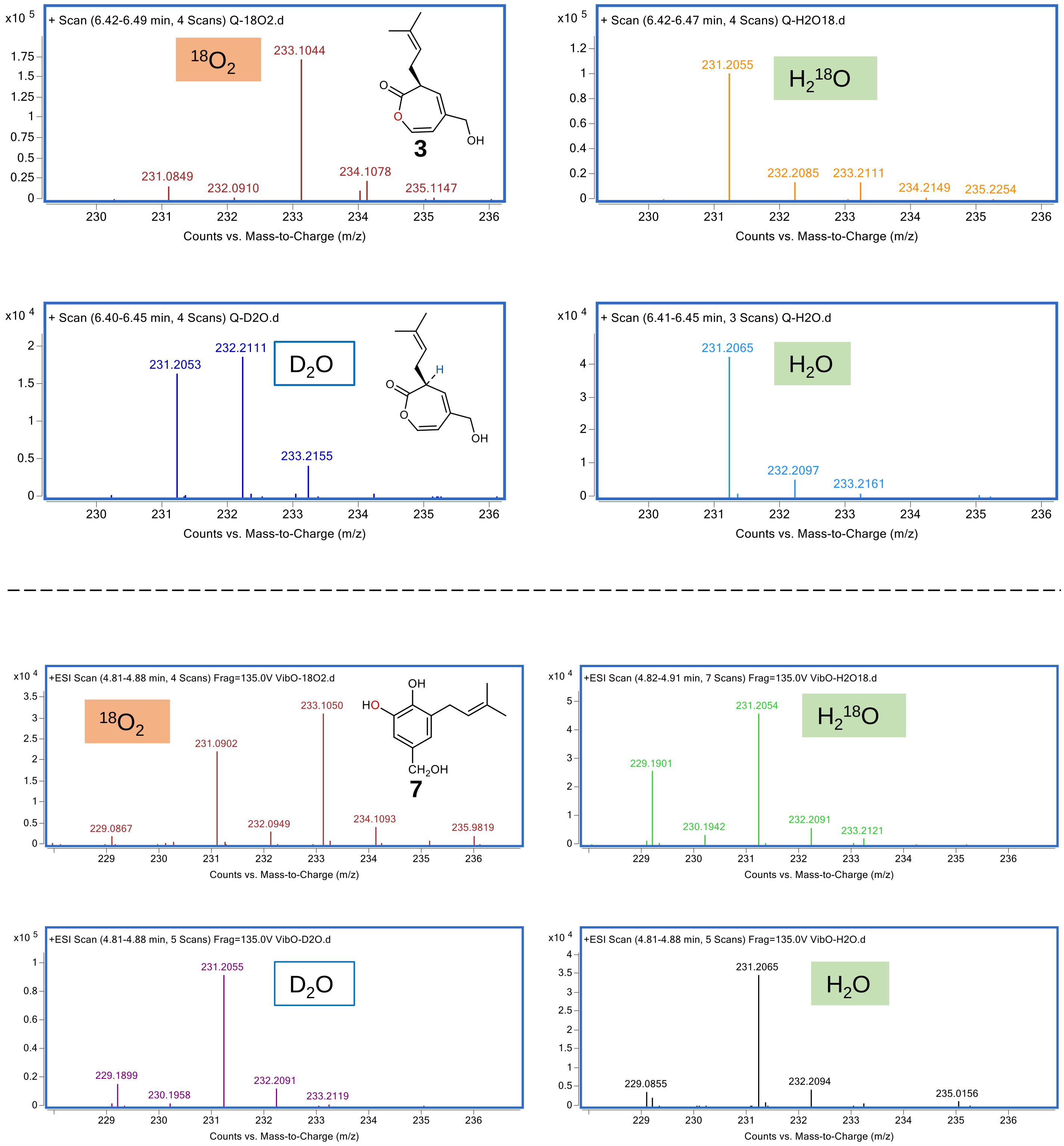

Supplementary Fig. 18. The original mass spectra data for 3 and 7 in Supplementary Fig. 17.

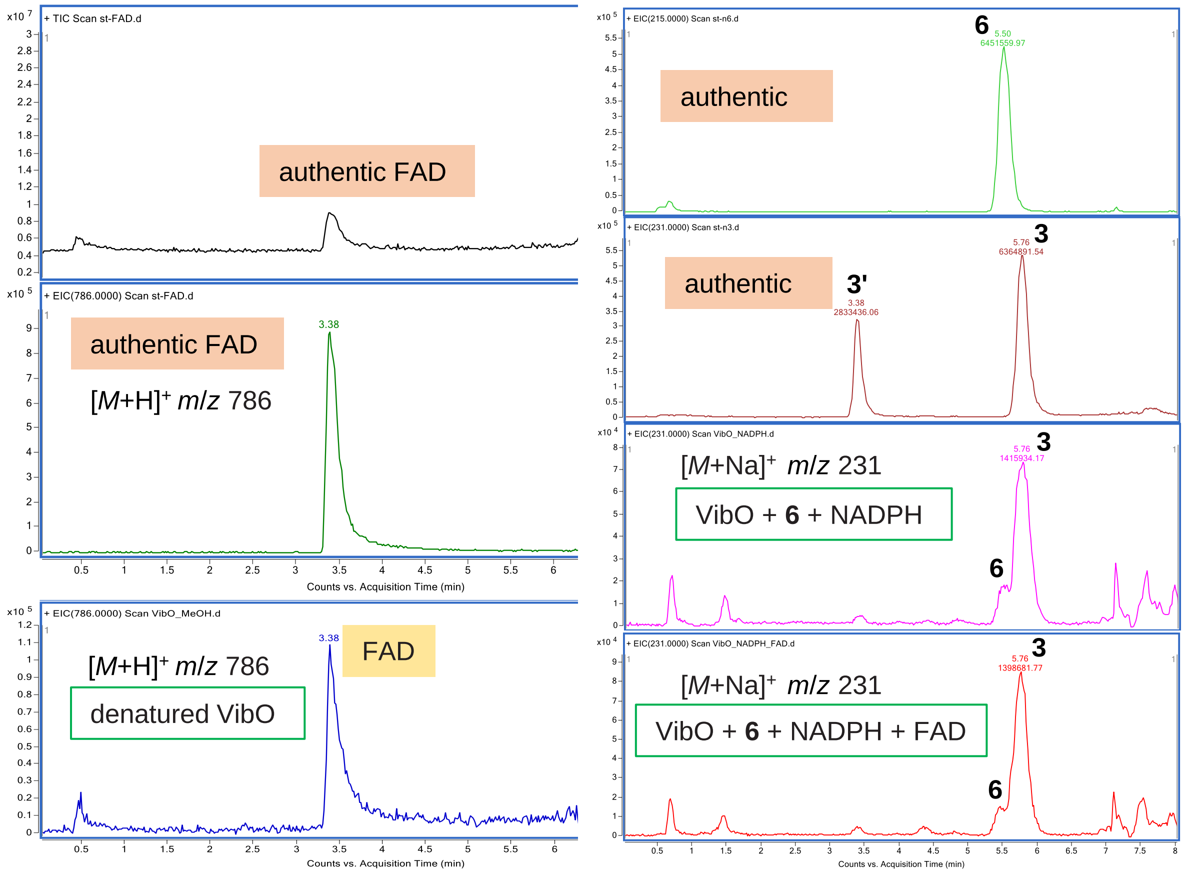

Supplementary Fig. 19. LC-MS analyses of FAD embedded in the VibO protein. (left) The authentic FAD and methanol-denatured VibO; chromatographic separation was performed with elution of 5%-35% B over 4.9 min and 100% B over the next 3.1 min where A was 0.1% formic acid and B was acetonitrile; the mass spectrometer was run in positive ionization mode. (right) Showing the peak areas of product **3** in reactions of VibO with or without the exogenous FAD; the chromatographic separation was the same as described in Supplementary Fig. 4.

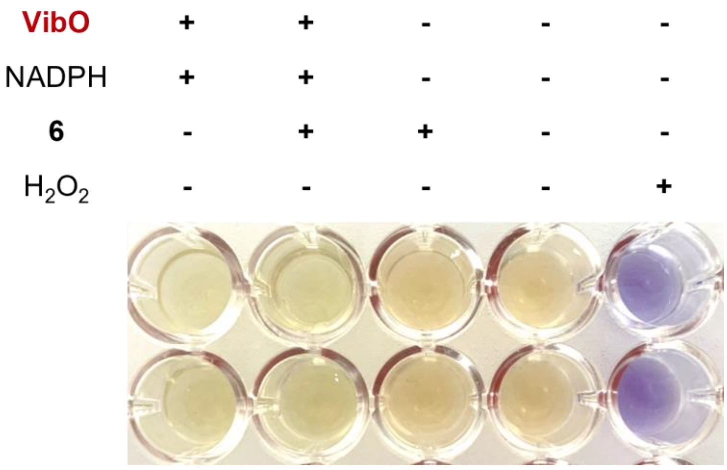

Supplementary Fig. 20. No detectable hydrogen peroxide in the VibO reaction. Each well was added with (+) or without (-) the purified VibO (16.7 μM), NADPH (0.1 mM), the substrate **6** (0.5 mM), using 50 μL of 50 mM sodium phosphate buffer pH 7.5 as a negative control (all "-") and hydrogen peroxide (H_2O_2, 1 μM) as a positive control. After incubating at 28 °C for 2 hours, mixtures were assayed using Hydrogen Peroxide Assay Kit. The purple color indicated the existence of H_2O_2. The experiments were repeated twice independently with similar results.

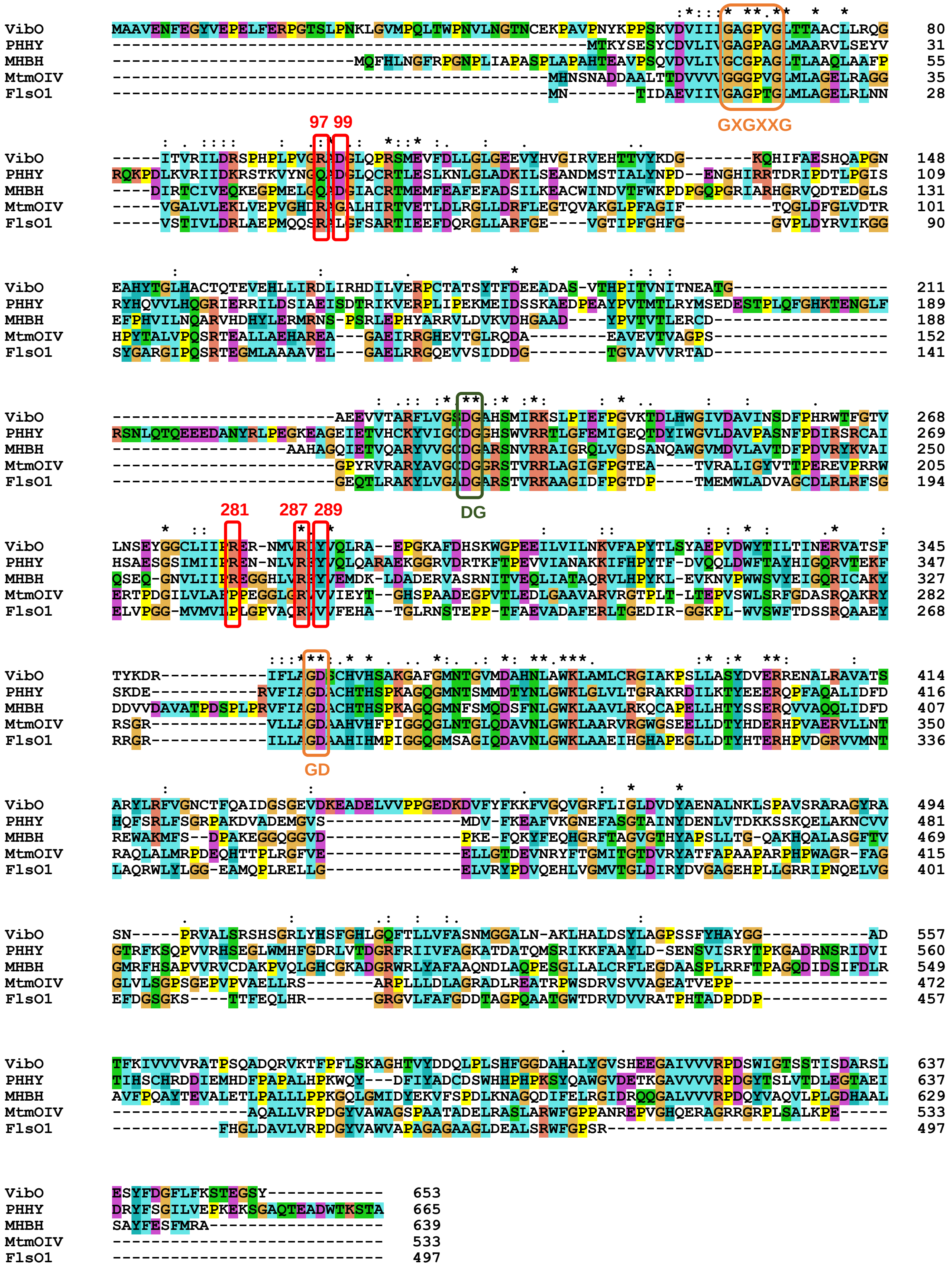

Supplementary Fig. 21. Sequence alignment of VibO with PHHY, MHBH, MtmOIV and FlsO1. Highly conserved sequences for flavin monooxygenases are found in VibO, including the motifs of GXGXXG and GD that are involved in FAD binding, as well as the DG fingerprint that is important for binding NAD(P)H. VibO residues of Arg97, Asp99, Arg281, Arg287 and Tyr289 were mutated in this study. "*" indicates a position with a fully conserved residue, while ":" and "." indicate different degrees of conservativeness. The sequence alignment was generated using the ClustalX2.1.

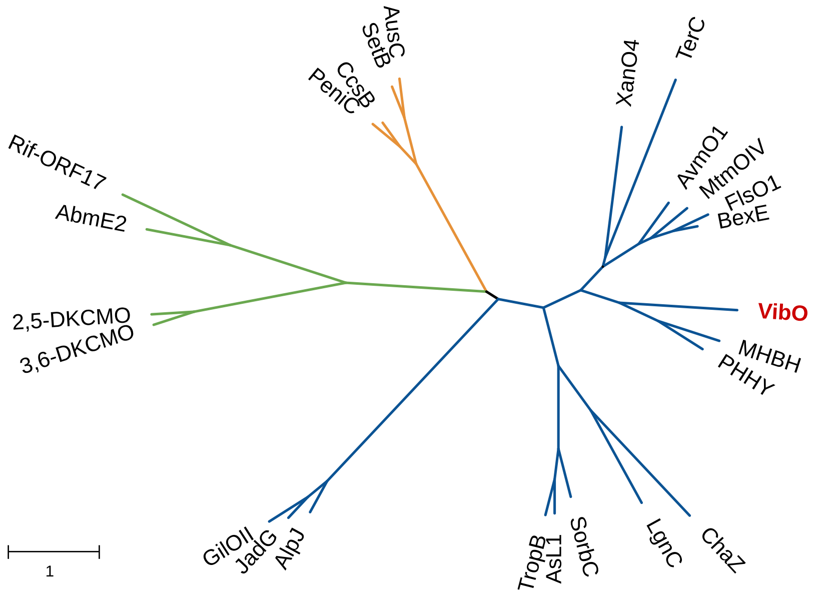

Supplementary Fig. 22. Phylogenetic analysis of VibO. Group A flavoprotein monooxygenases (type O BVMOs and aromatic hydroxylases) are in blue; Group B flavoprotein monooxygenases (type I BVMOs) are in orange; Group C flavoprotein monooxygenases (type II BVMOs) are in green. The tree was constructed by FastTree (2.1.10) with Maximum likelihood method. The amino acid sequences are as follows: VibO (*Boreostereum vibrans*, PDB: 7YJ0_A); PHHY (*Trichosporon cutaneum*, PDB: 1PN0_A); MHBH (*Comamonas testosteroni*, PDB: 2DKI_A); MtmOIV (*Streptomyces argillaceus*, PDB: 4K5R_A);FlsO1 (*Micromonospora rosaria*, PDB: 7VWP_A); BexE (*Amycolatopsis orientalis subsp*. *Vinearia*, PDB: 4X4J_A); AvmO1 (*Streptomyces* sp. TP-A0867, GenBank: QSV12647.1); XanO4 (*Streptomyces flavogriseus*, GenBank: ADE22300.1); TerC (*Aspergillus terreus*, UniProtKB/Swiss-Prot: Q0D1P1.1); ChaZ (*Streptomyces chartreusis*, GenBank: AXS67818.1); LgnC (*Streptomyces* sp. MA37, GenBank: AIZ66878.1); TropB (*Talaromyces stipitatus*, GenBank: DAA64700.1); AsL1 (*Sarocladium schorii*, UniProtKB/Swiss-Prot: A0A2U8U2L6.1); SorbC (*Penicillium chrysogenum*, UniProtKB/Swiss-Prot: B6HN76.1); AlpJ (*Streptomyces ambofaciens*, PDB: 5F9P _A); JadG (*Streptomyces venezuelae*, GenBank: AAV52247.1); GilOII (*Streptomyces griseoflavus*, GenBank: AAP69583.1); AusC (*Aspergillus nidulans*, UniProtKB/Swiss-Prot: C8VE79.1); SetB (*Aspergillus duricaulis* CBS 481.65, JGI: Protein ID 270787); CcsB (*Aspergillus clavatus*, GenBank: AIE17460.1); PeniC (*Penicillium griseofulvum*, GenBank: QDO73504.1); Rif-ORF17 (*Amycolatopsis mediterranei*, NCBI: WP_013222574.1); 2,5-DKCMO (*Pseudomonas putida*, GenBank: BAN13304.1); 3,6-DKCMO (*Pseudomonas putida*, PDB: 5AEC _A); AbmE2 (*Streptomyces koyangensis*, GenBank: AVI57411.1).

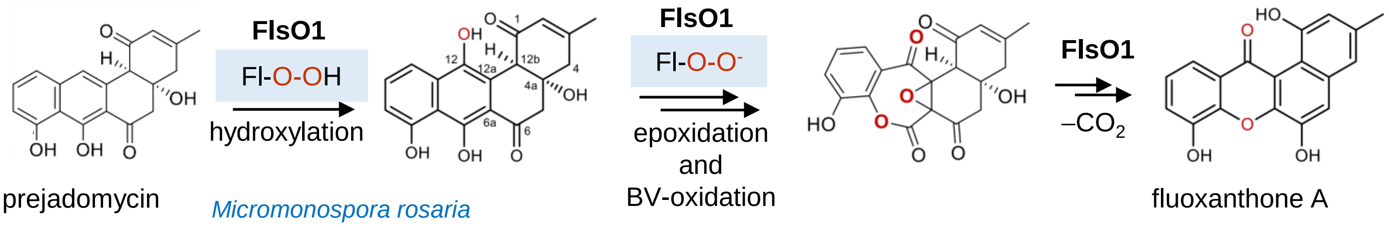

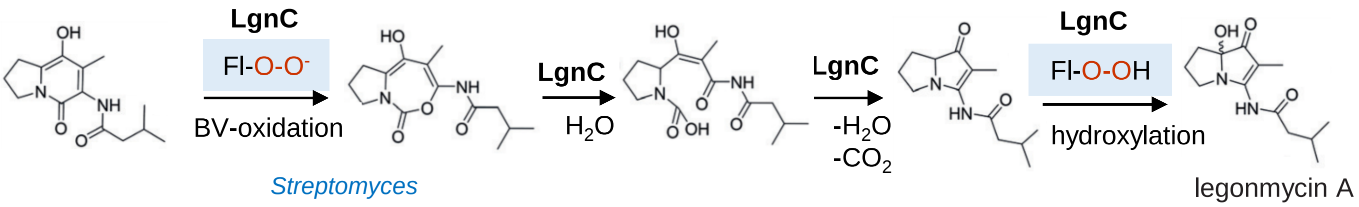

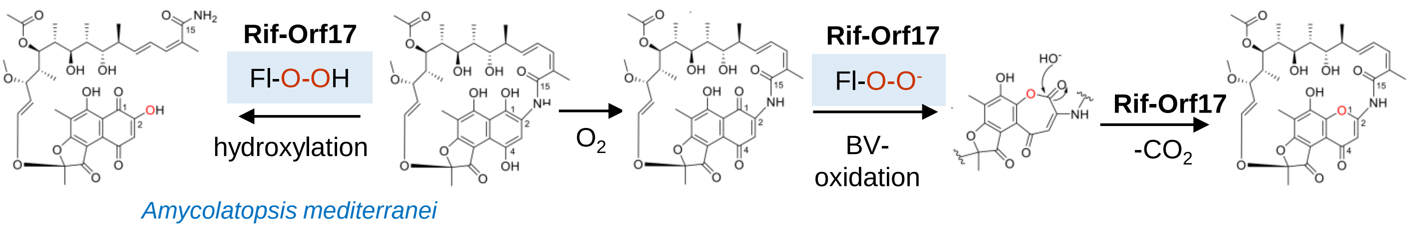

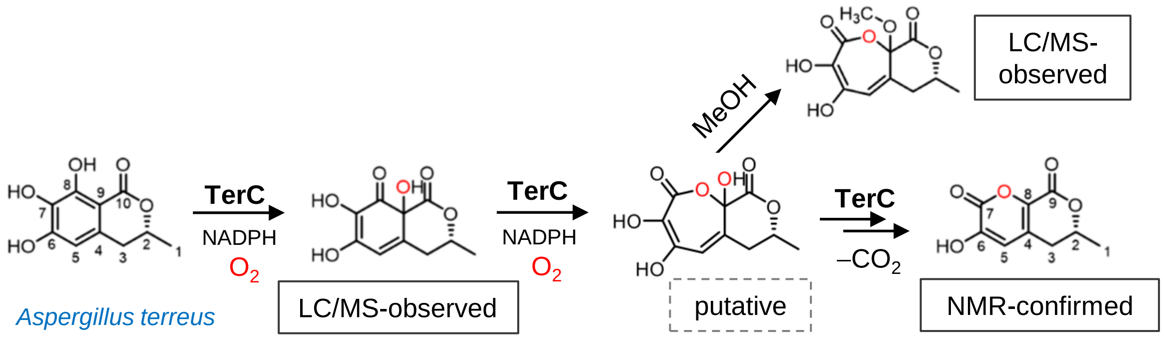

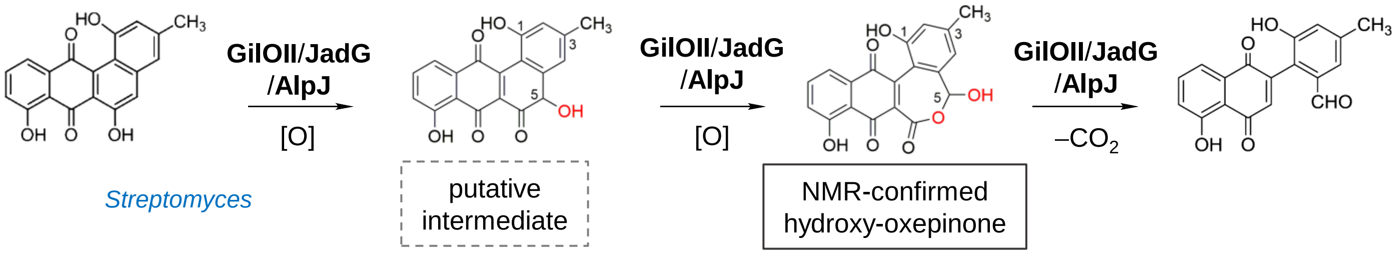

Continued on next page...

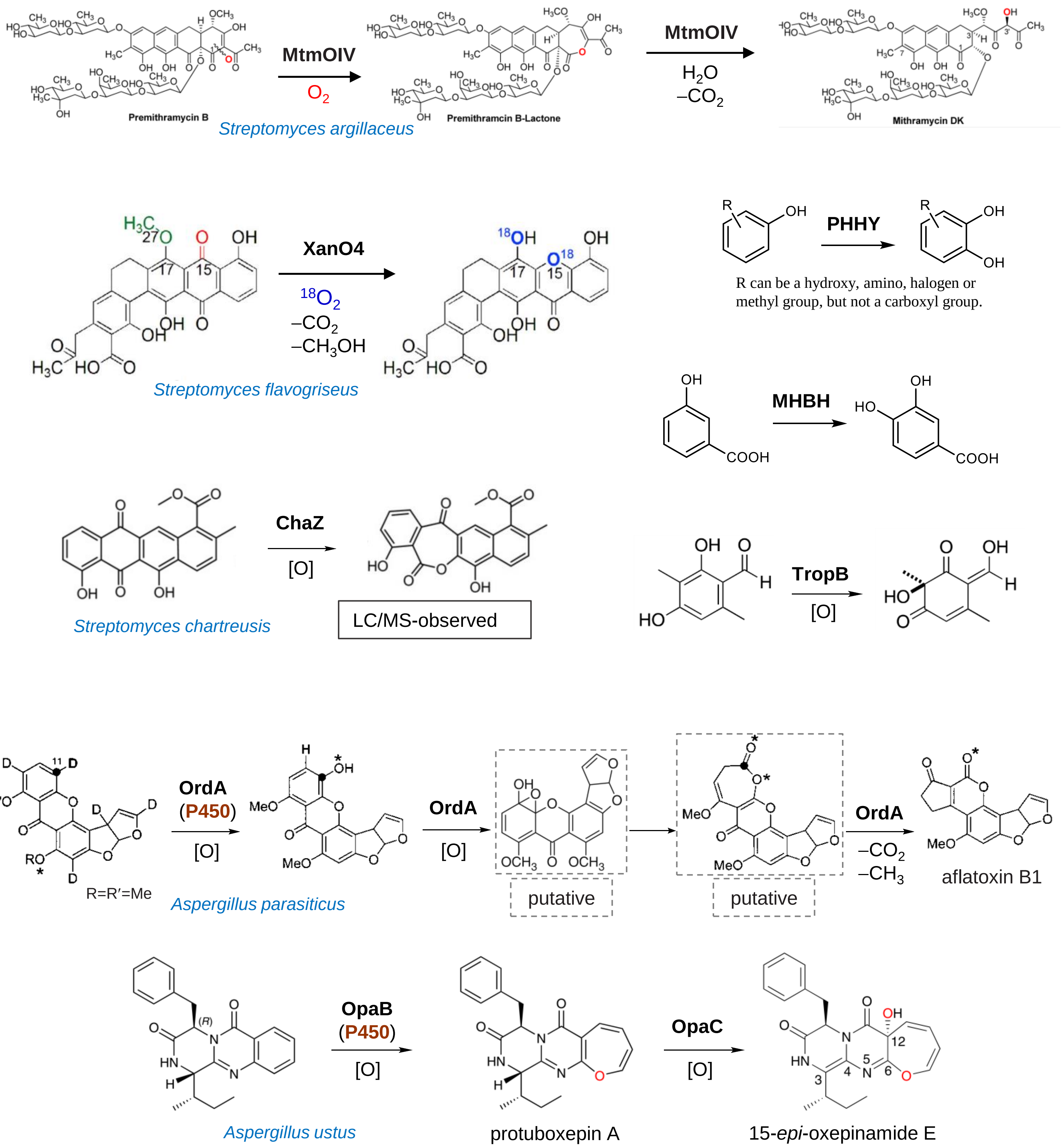

Supplementary Fig. 23. BV-oxidation or hydroxylation catalyzed by some flavoenzymes and P450s. Both Fl-O-O⁻ and Fl-O-OH have been proposed in mechanisms for FlsO1, LgnC, and Rif-Orf17.

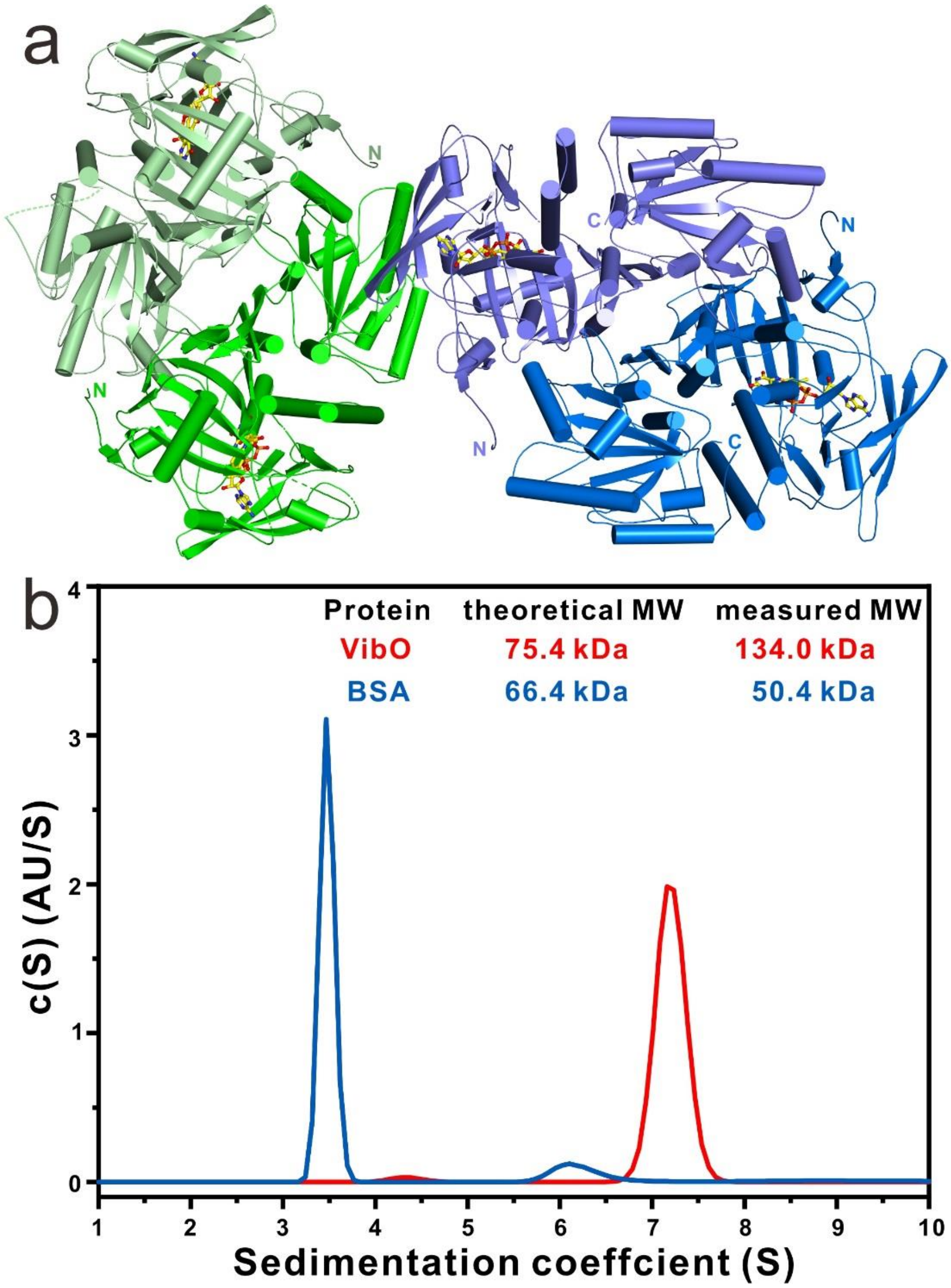

Supplementary Fig. 24. VibO forms a stable dimer. (**a**) The ribbon-stick representation showing the four VibO molecules that form two stable dimers in an asymmetric unit. (**b**) The sedimentation velocity data of VibO and BSA proteins showing that VibO forms a stable dimer in solution. In this drawing, BSA is included as a standard control sample, and "MW" stands for molecular weight. Source data are provided as a Source Data file.

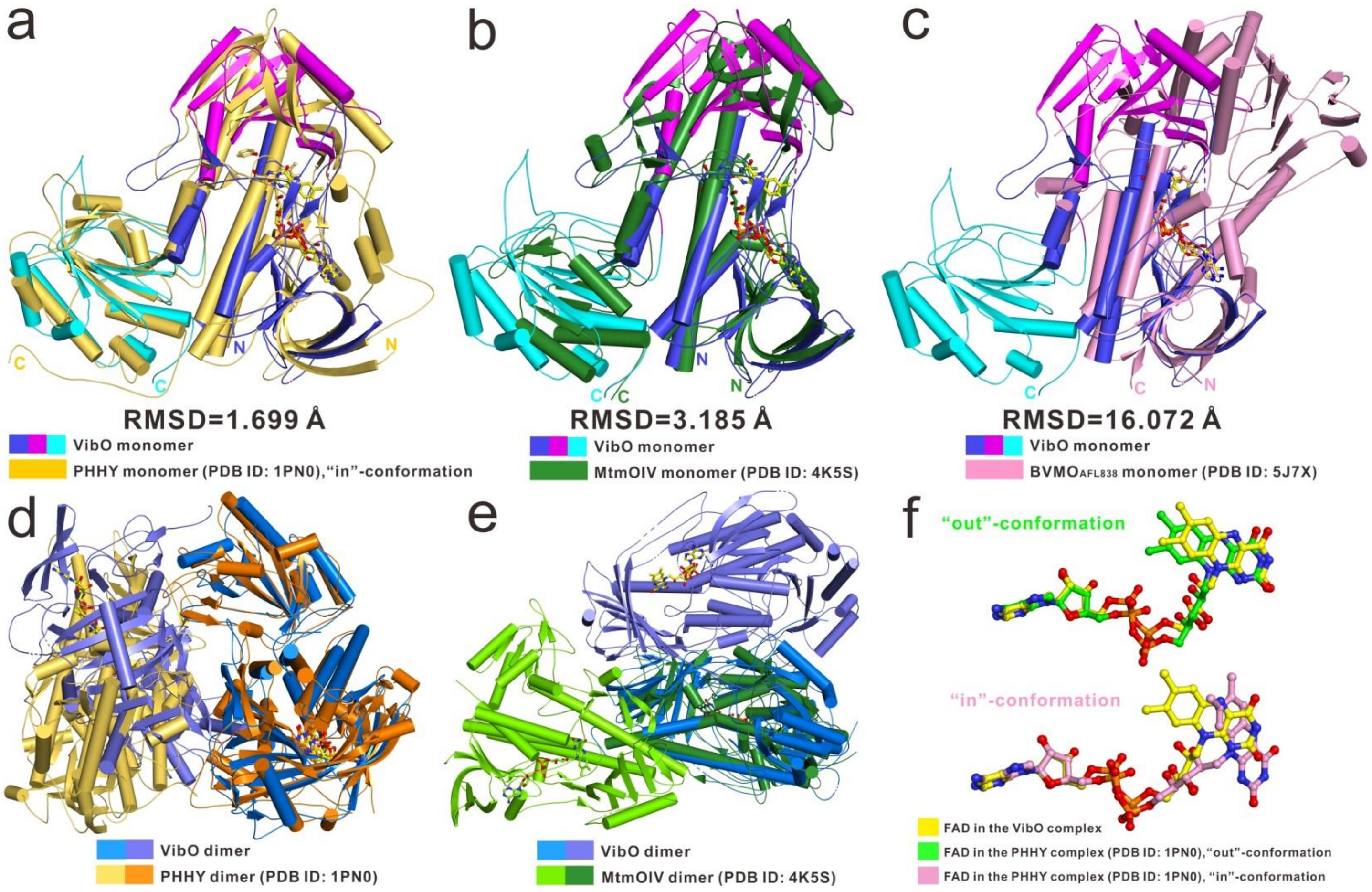

Supplementary Fig. 25. Structural comparison analyses of VibO and other relevant flavin-dependent monooxygenases. (**a** -**c**) The combined ribbon and stick representation showing the overall structure comparison of the monomeric VibO and the monomeric PHHY in complex with the co-factor FAD that adopts an "in"-conformation (yelloworange, PDB ID: 1PN0) (**a**), the monomeric MtmOIV (forest green, PDB ID: 4K5S) (**b**), or the monomeric $BVMO_{AFL838}$ (pink, PDB ID: 5J7X) (**c**). (**d** and **e**) Structural comparison of the dimeric VibO (colored by marine and slate) with dimeric PHHY (colored by orange and yelloworange, PDB ID: 1PN0) (**d**), or dimeric MtmOIV (colored by forest green and chartreuse, PDB ID: 4K5S) (**e**). (**f**) Structural comparisons of the conformation of FAD in the VibO complex (yellow) and that of FAD found in the PHHY complex (PDB ID: 1PN0). Notably, the FAD colored in green and pink represent the "out"-conformation and "in"-conformation found in the PHHY complex, respectively.

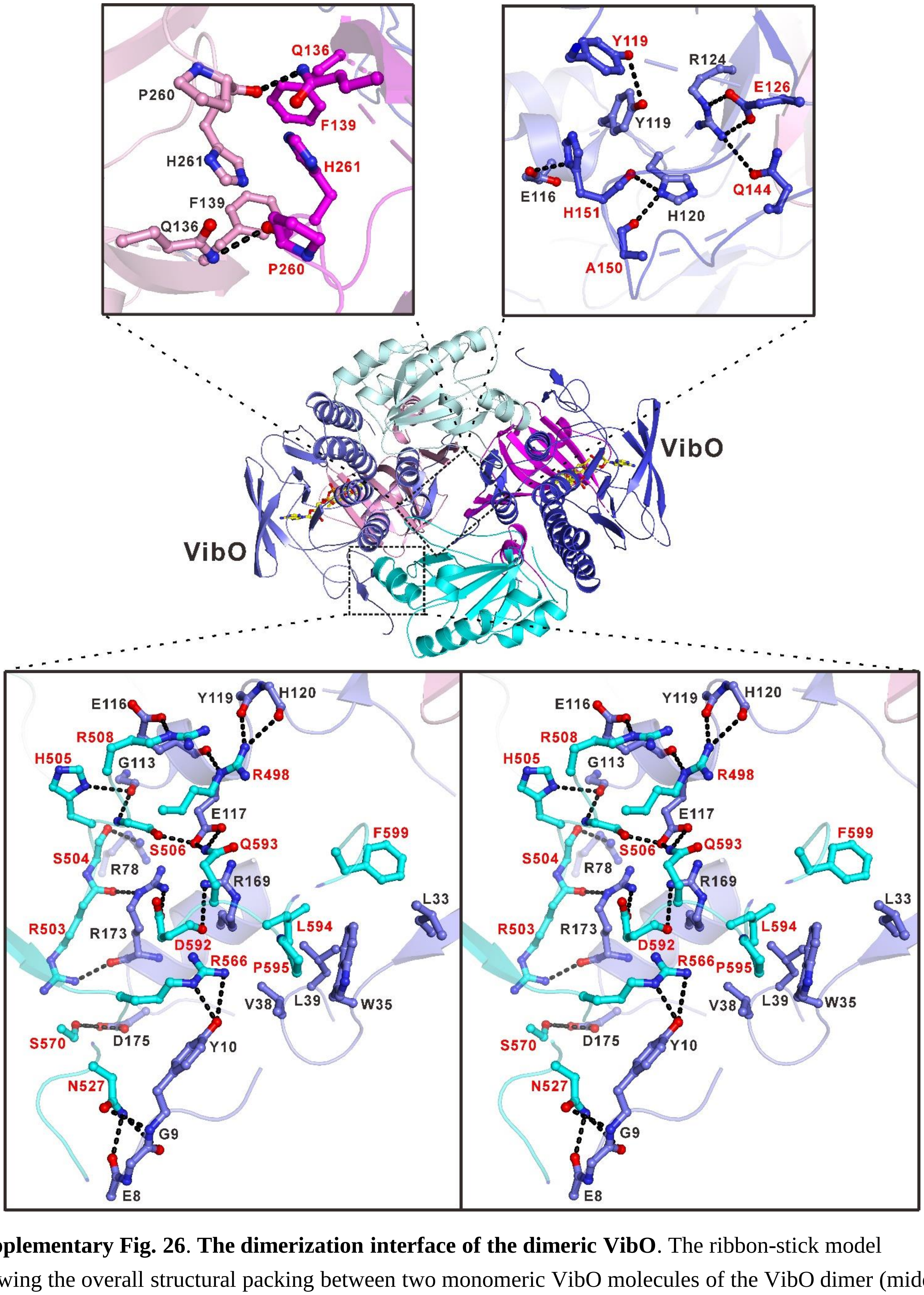

Supplementary Fig. 26. **The dimerization interface of the dimeric VibO**. The ribbon-stick model showing the overall structural packing between two monomeric VibO molecules of the VibO dimer (middle panel). The enlarged view of ribbon-stick representation showing the three different binding interfaces between two monomeric VibO: the middle domain/middle domain interface (left in the top panel), the FAD-binding domain/FAD-binding domain interface (right in the top panel), and the FAD-binding domain/C-terminal domain interface (bottom panel). The hydrogen bonds and salt bridges involved in the interactions are shown as dotted lines.

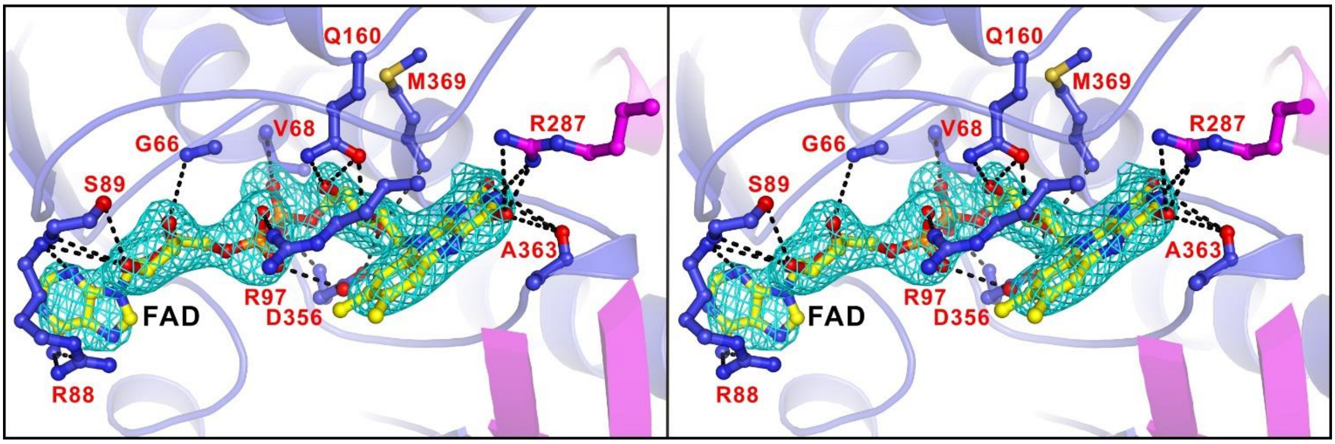

Supplementary Fig. 27. **The molecular interface of VibO and FAD interaction**. A stereo view of the ribbon-stick-ball model showing the detailed binding interface between VibO and the cofactor FAD in the VibO/FAD complex. The hydrogen bonds and salt bridges involved in the interactions are shown as dotted lines. The F_O-F_C map is shown as a cyan mesh, and is calculated from the final PDB file by omitting the FAD molecule and contoured at 2.5σ.

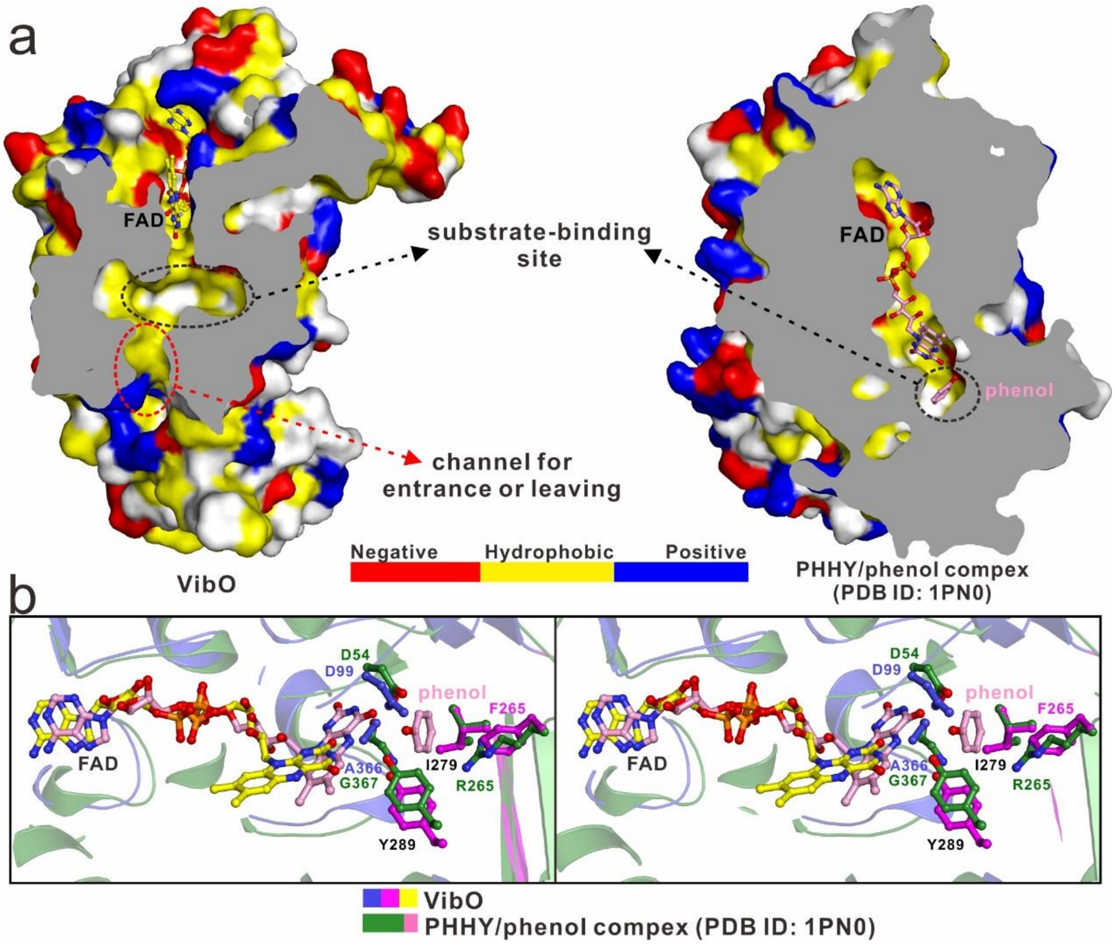

Supplementary Fig. 28. **Structural analyses of the substrate-binding pocket of VibO**. (**a**) Surface representations showing the substrate-binding pocket as well as the proposed substrate entrance or product leaving channel of VibO (left), and the substrate-binding pocket observed in the PHHY/phenol complex (PDB ID: 1PN0) (right), in which the bound FAD adopts an "in"-conformation. In the surface representations, the hydrophobic residues are shown in yellow, the positively charged residues are shown in blue, the negatively charged residues are shown in red, and the uncharged polar residues are shown in gray. (**b**) Stereo view of the ribbon-stick-ball representation showing the comparison of the key residues in the active sites of VibO and PHHY.

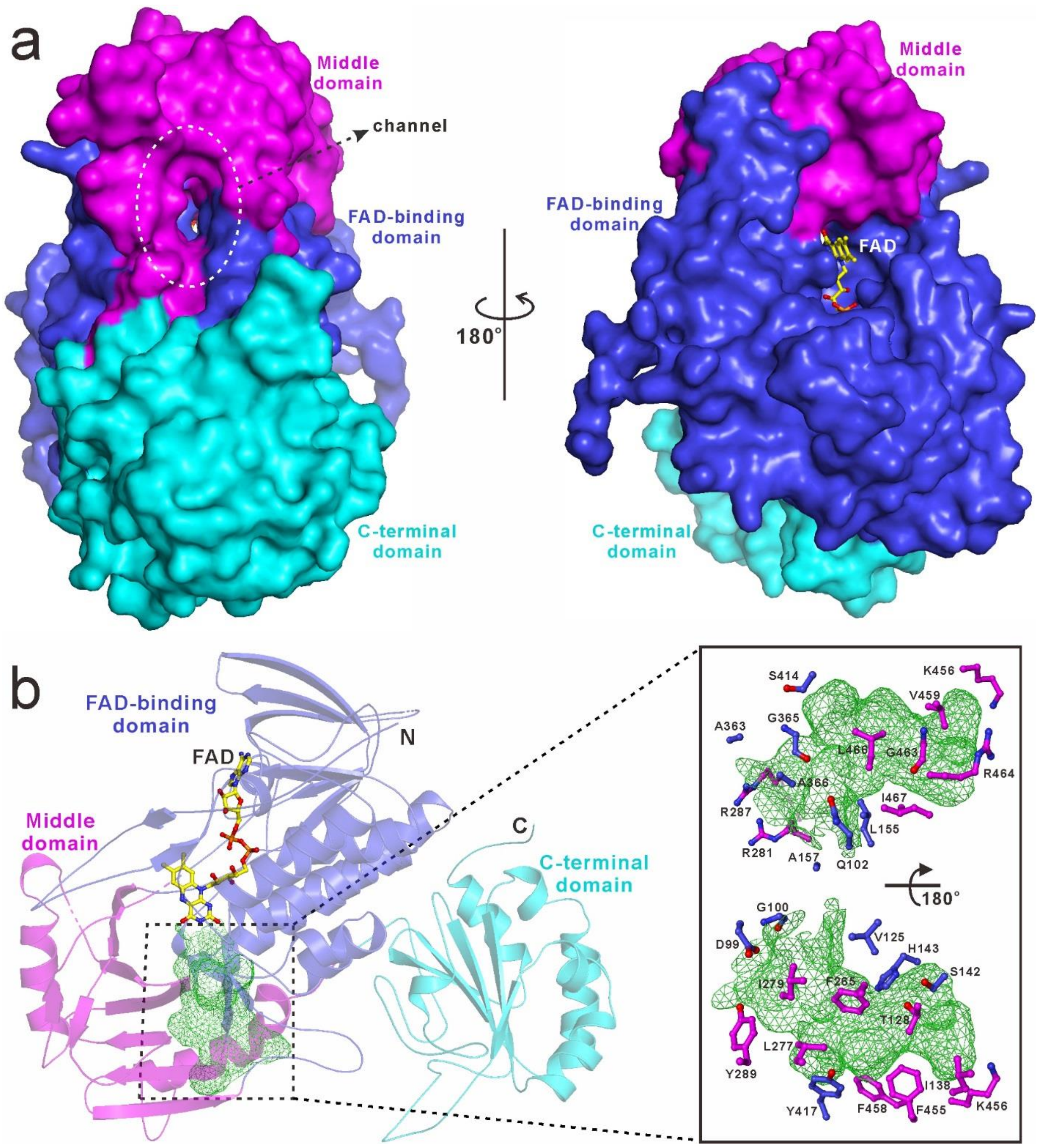

Supplementary Fig 29. **Structural analyses of the unique channel proposed for the substrate entrance or product leaving of VibO.** (**a**) Combination of the surface representation (for the monomeric VibO) and the stick-ball model (for the bound FAD) showing a unique channel formed between the FAD-binding domain and the middle domain of VibO is located at the opposite side of the FAD-binding site. (**b**) Combined cavity surface and ribbon-stick-ball representation showing the substrate-binding pocket and the unique channel of VibO (shown in green wireframe mode), which are located between the middle domain and the FAD-binding domain (left), and the hydrophobic and polar residues for the assembly of this active site of VibO (right).

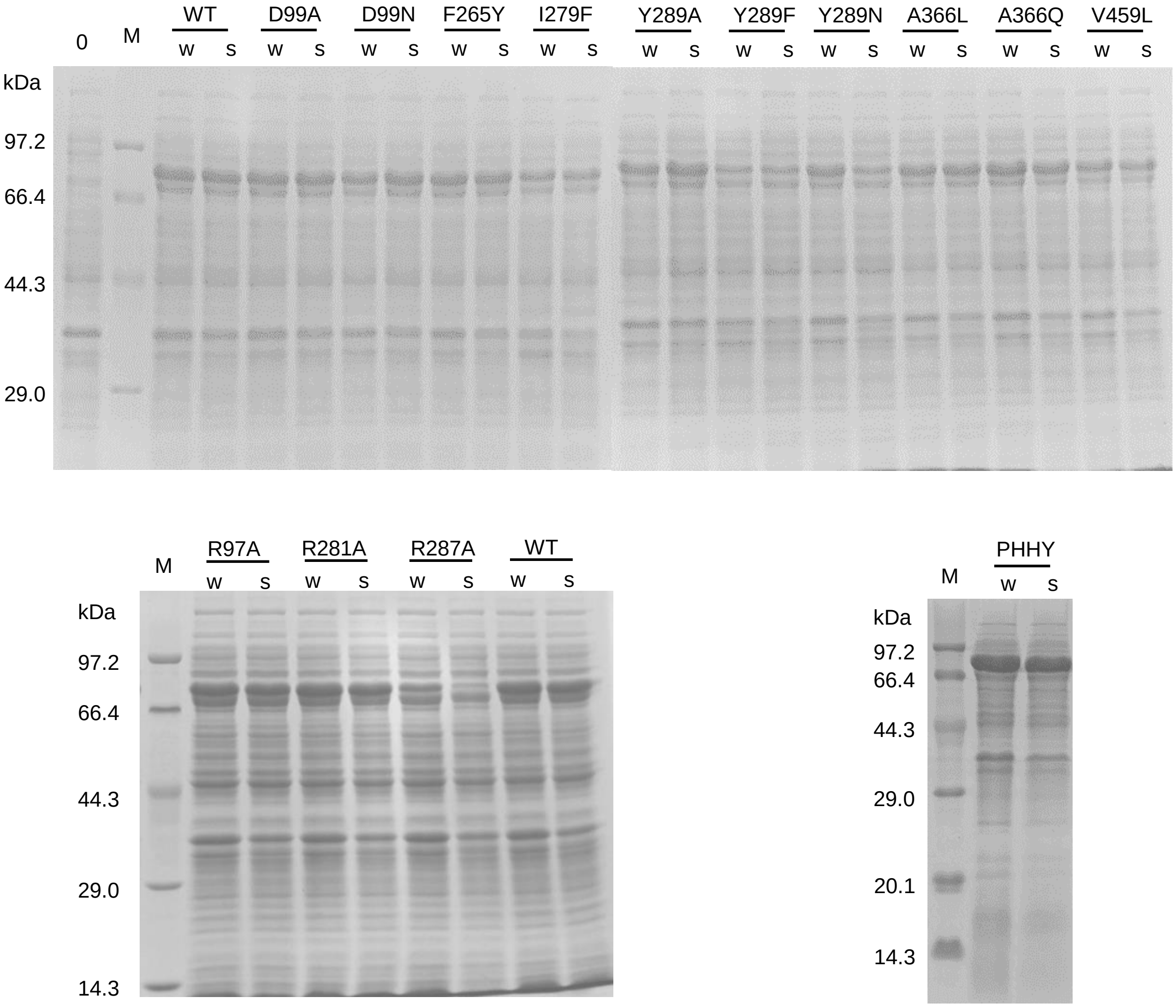

Supplementary Fig. 30. The SDS-PAGE analysis of VibO wild-type (WT) and variants as well as PHHY (phenol hydroxylase). M, protein size marker; 0, empty vector as blank; w, whole proteins; s, soluble proteins. The experiments were repeated twice independently with similar results and the original photographs were supplied at the end of this file.

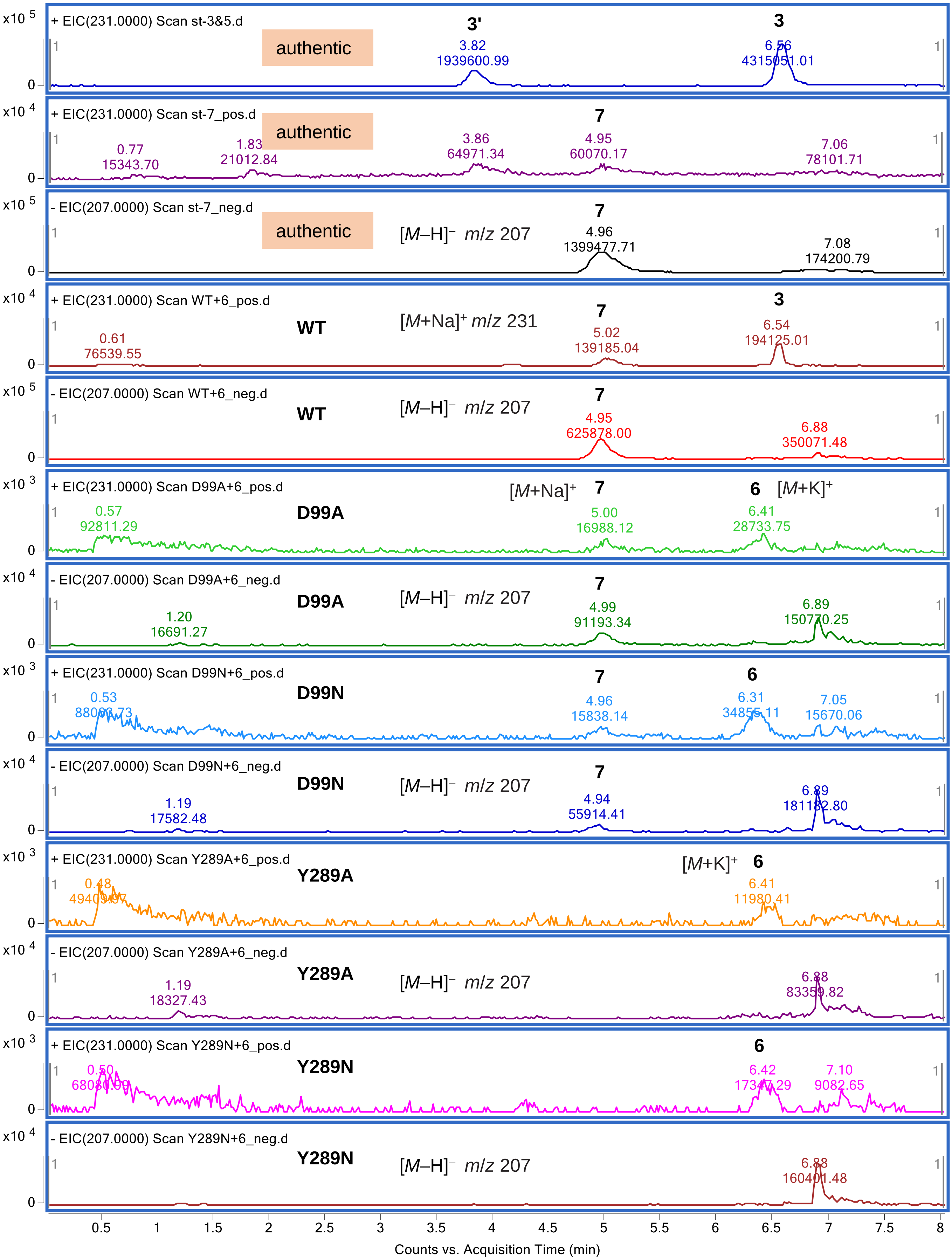

Supplementary Fig. 31. **LC-MS analyses of wild-type (WT) and mutants of VibO**. The chromatographic separation was the same as described in Supplementary Fig. 4.

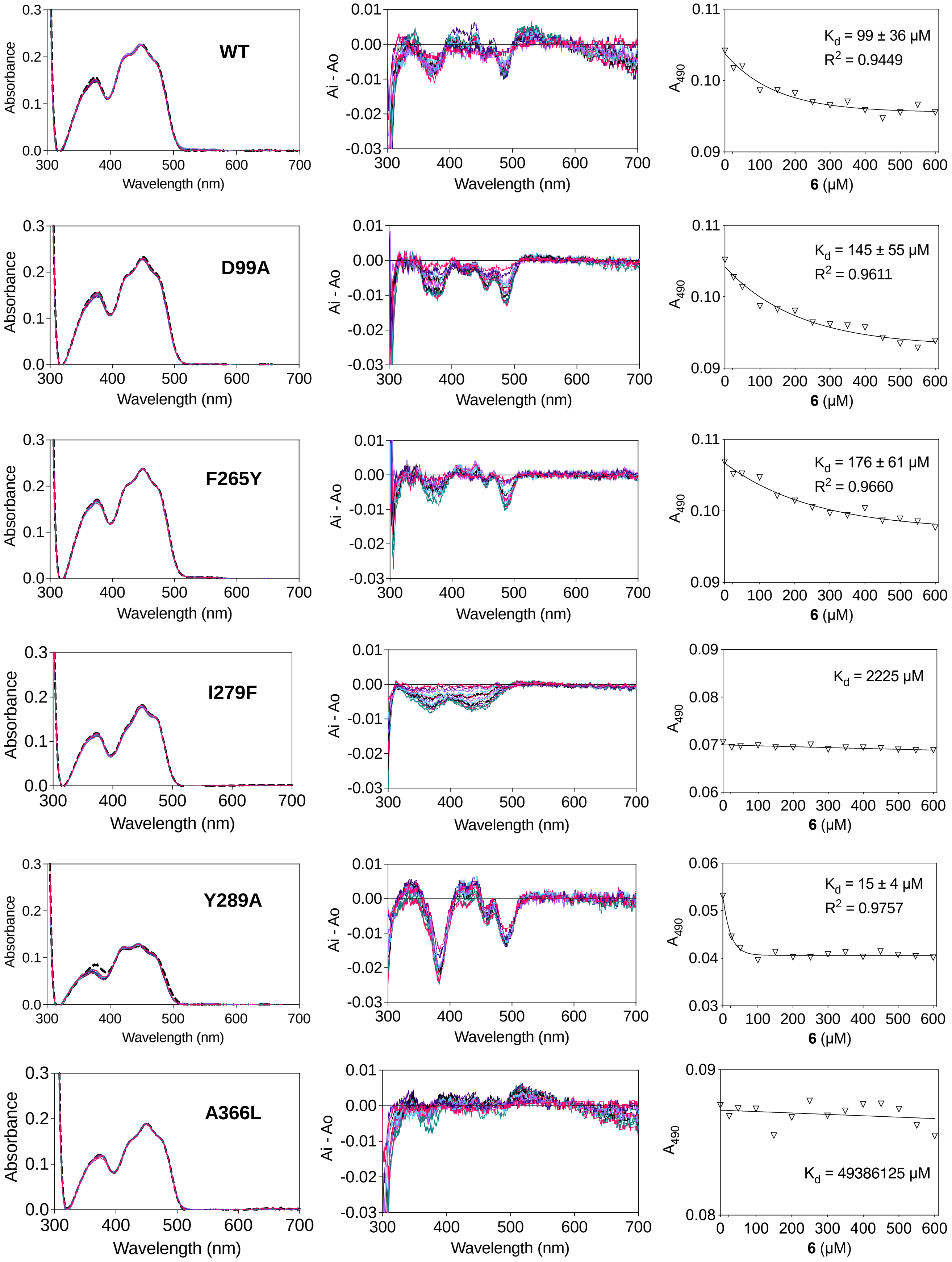

Supplementary Fig. 32. Substrate binding titrations with VibO or its variants. The substrate-free VibO (or its variant) is shown with a black dashed line. Thin colored solid lines show spectra collected after additions of substrate **6** up to 0.6 mM All spectral measurements were carried out in a cuvette containing 34 μM enzyme on a NanoDrop™ One UV-Vis Spectrophotometer (Thermo Scientific) and provided in Source Data file. The scan range was 300-700 nm in 0.5 nm increments. Turbidity correction was performed using the Rayleigh scattering equation and the baseline correction wavelength was set at 580 nm. At each stage of the titration, a difference spectrum (Ai – Ao) was generated by subtracting the substrate-free spectrum. Substrate-induced absorbance difference against the relevant concentration of **6** was collected at 490 nm for K_d.

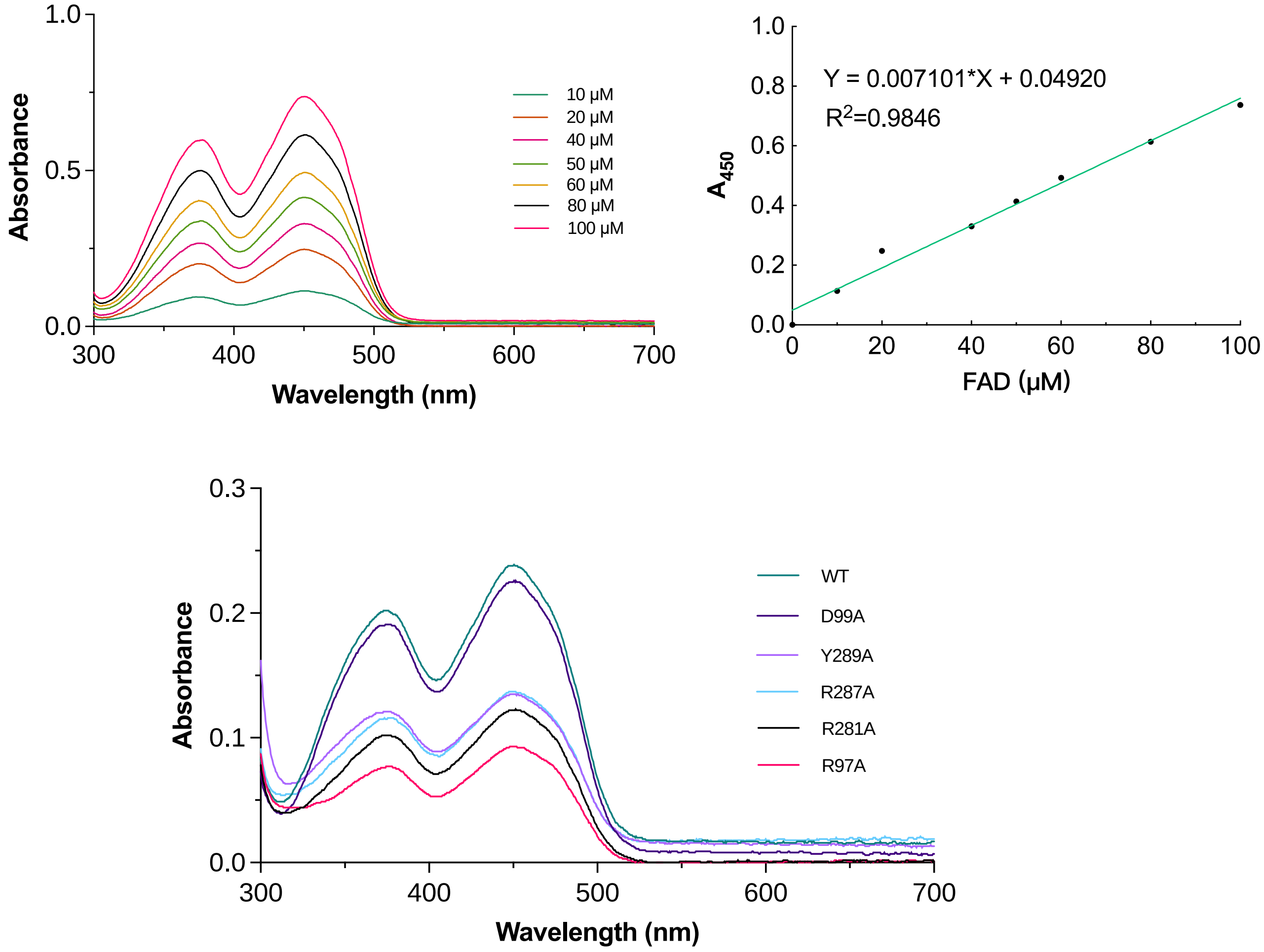

Supplementary Fig. 33. Determination of FAD binding. 34 µM of each protein were prepared in 50 mM phosphate buffer pH 7.5 and boiled at 100°C for 10 minutes. Supernatant obtained by centrifugation. All FAD binding measurements were carried out on a NanoDrop™ One UV-Vis Spectrophotometer (Thermo Scientific) and provided in Source Data file. The scan range was 300-700 nm in 0.5 nm increments. The absorbance of the denatured protein at 450 nm determines the FAD concentration in each protein sample. Compared with the wild type, the FAD binding for each mutant was 93% for D99A, 45% for Y289A, 47% for R287A, 38% for R281A, and 23% for R97A.

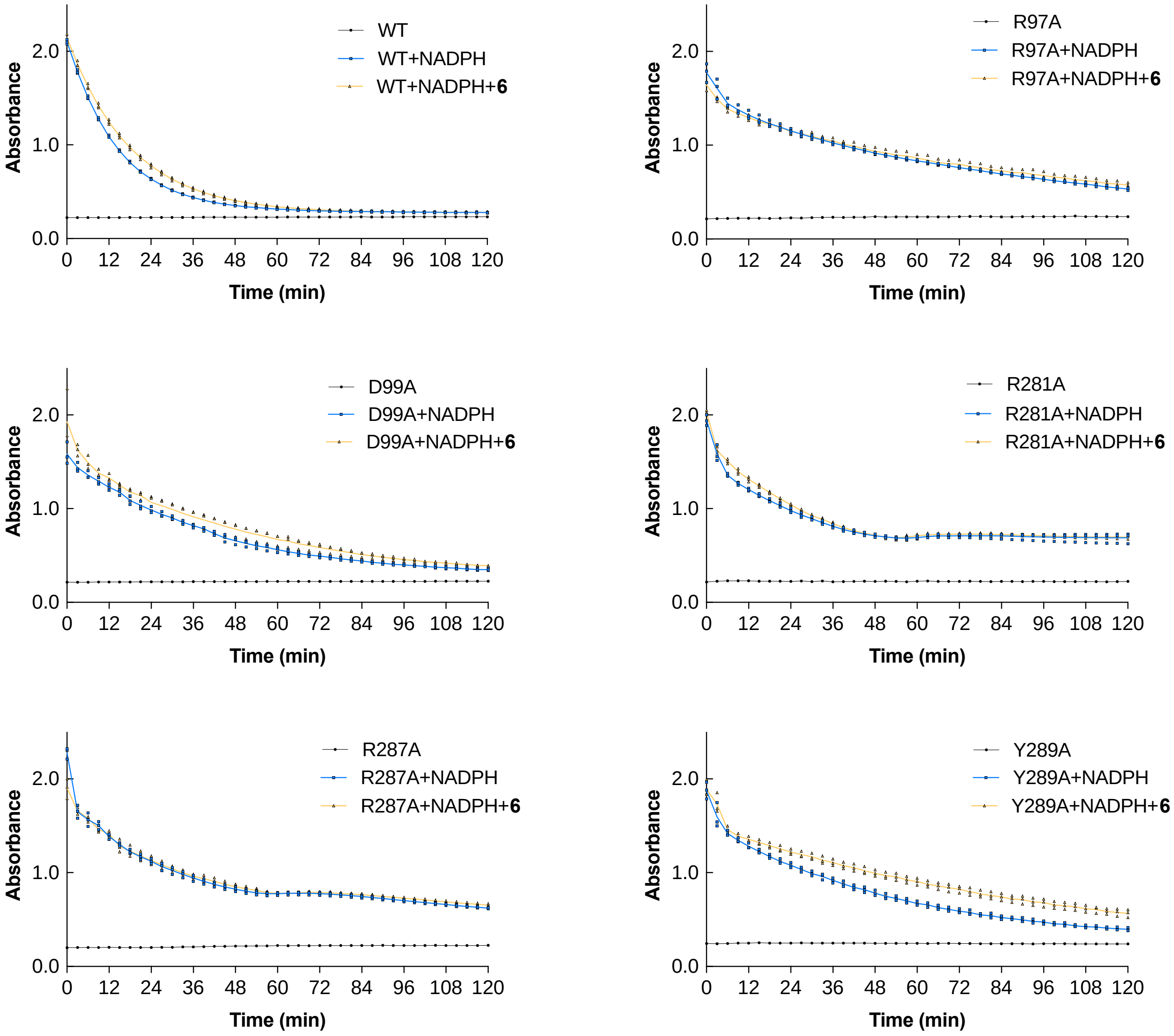

Supplementary Fig. 34. Reduction of VibO or variants by NADPH. Reaction of 17 μM enzyme and 1 mM NADPH in the absence or presence of **6** (0.5 mM) was carried out in a 96-well plate. All spectral measurements were performed on a BioTek Cytation 1 cell imaging multimode reader at 28 °C and provided in Source Data file. Depletion of NADPH was measured at 340 nm for 120 min.

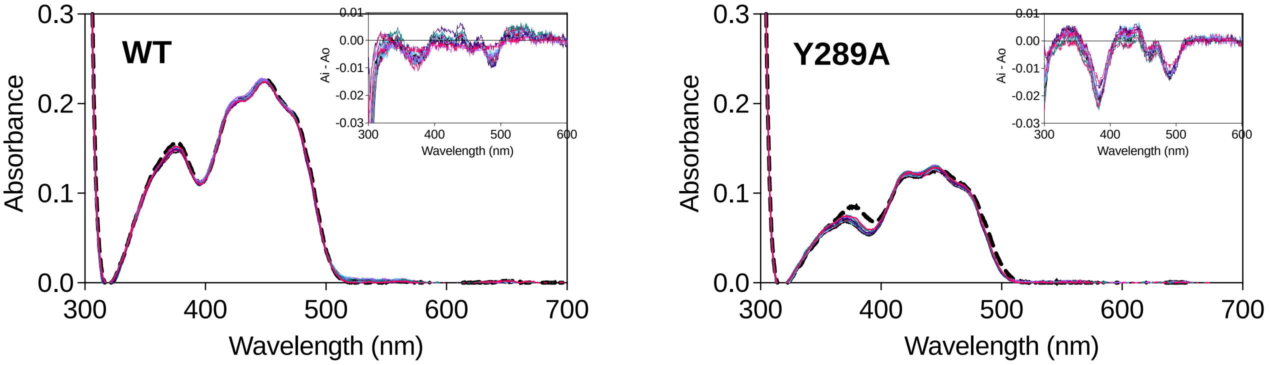

Supplementary Fig. 35. The shift in the absorption spectra of VibO. The inset shows the difference spectra. In the absence of substrate **6** (dash line), the shoulder peak at 430 nm was more pronounced in Y289A (**b**) than the wild-type VibO (**a**). With the titration of substrate **6**, the differential absorption of Y289A was also significantly different from that of the wild type. These seem to hint about a minor change of the microenvironment around the FAD, which might result in a somewhat positioning perturbation on FAD, and thereby cause Y289A′s inability to convert **6** into either **3** or **7**.

As shown in Figure 4A of the literature[4] about phenylacetone monooxygenase from *Thermobifida fusca*, binding of $NADP^+$ causes the absorbance peak shift from 383 nm to 366 nm and the peak at 440 nm to develop a more prominent absorbance shoulder at 480 nm. The maximum difference in absorbance is at 387 nm. The observed spectral and kinetic data suggest that the kinetic event reflects a conformational change in which the direct environment of the flavin cofactor is affected.

For acetone monooxygenase from *Gordonia* sp. strain TY-5 (see Figure 3a,b in the literature)[5], there is also a blue shift in the absorbance peak at 380 and the peak at 440 nm shifts to 450 nm with a sizable shoulder at 430 nm. The maximum difference in absorbance is at 467 nm. The distinct spectral shifts are likely due to minor structural variations in the active site induced by the binding of $NADP^+$.

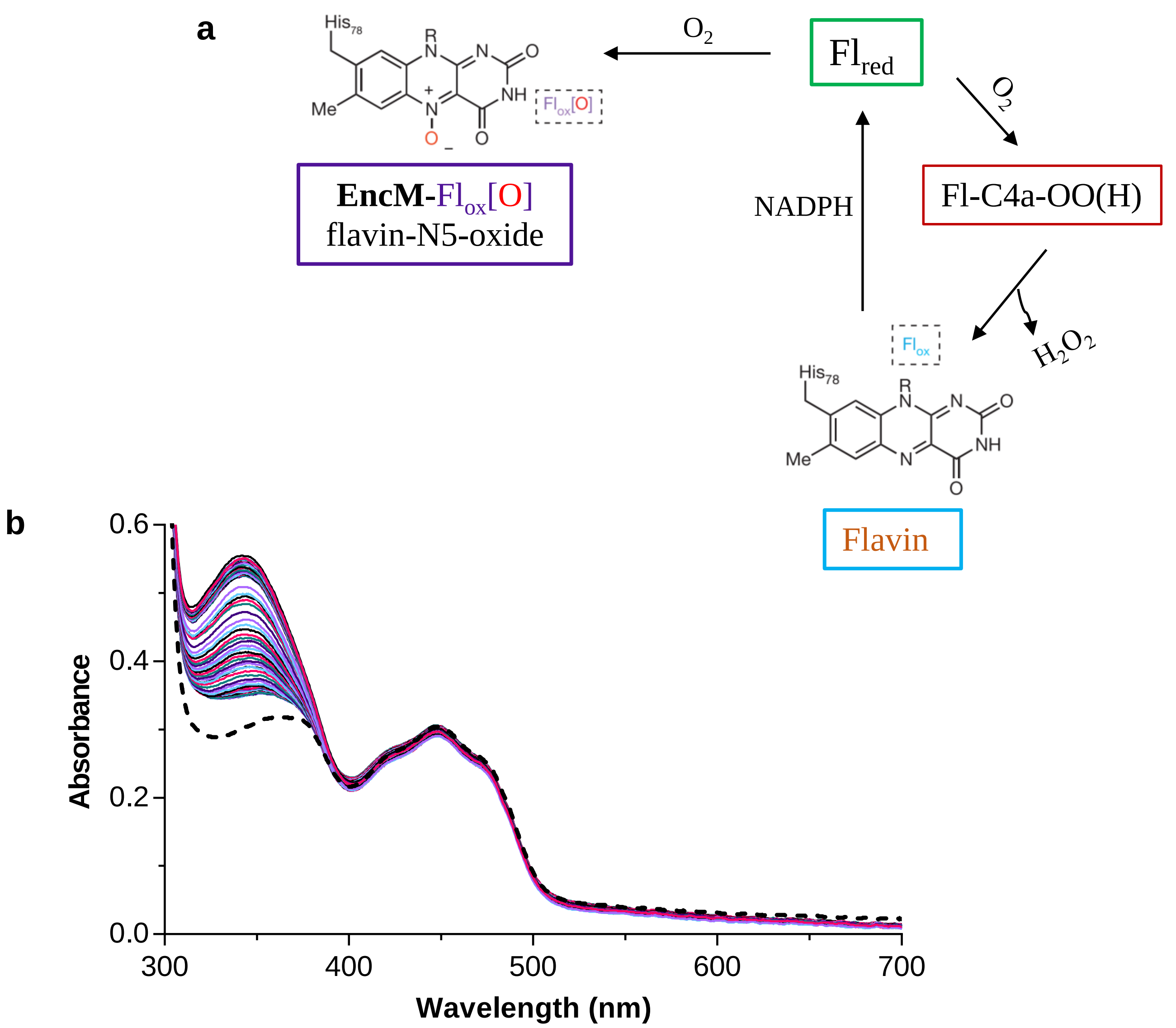

Supplementary Fig. 36. a Spectral features of flavin species in EncM as shown in Figure 3c of the literature[6] where molar absorption coefficients were ε_{460} = 9,600 M^{-1} cm^{-1} for EncM-Fl_{ox}[O] (Fl_{ox}[O] as isolated, catalytically active) and ε_{450} = 11,900 M^{-1} cm^{-1} for EncM-Fl_{ox} (Fl_{ox}, after multiple substrate turnovers, catalytically inactive). **b** The time-course spectral analysis of VibO with NADPH. Reaction of VibO (34 µM) with NADPH (50 µM) was carried out in a cuvette. The spectra were collected immediately after addition of NADPH on a NanoDrop™ One UV-Vis Spectrophotometer (Thermo Scientific) up to 643.49 seconds and provided in Source Data file. The scan range was from 300 to 700 nm in 0.5 nm increments. The black dash indicates the spectrum at 0 second.

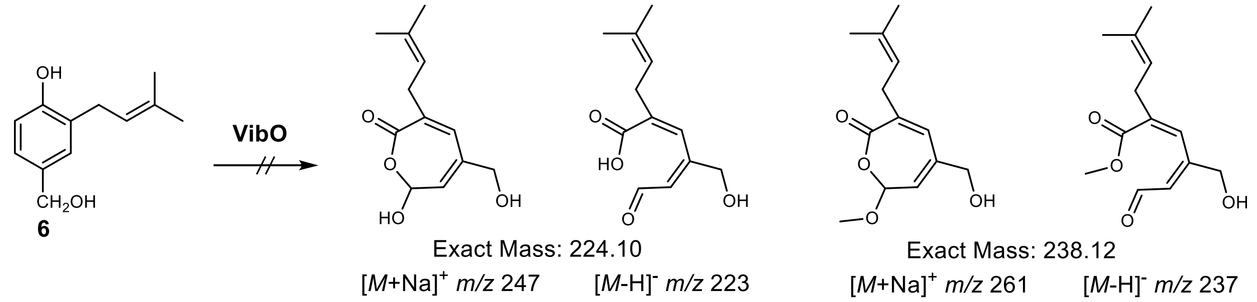

Supplementary Fig. 37. No detectable hydroxy-oxepinones in the VibO-catalyzed reactions. Time-course reactions of VibO (17 μM) incubating with NADPH (0.1 mM, 1 mM, 5 mM) and **6** (0.5 mM) at 28 °C for a period of time (10 min, 30 min, 1 h, 2 h), each was quenched with methanol and analyzed by LC-MS, using the boiled inactive VibO as control. In all the reactions, however, none of the putative hydroxy-oxepinones/methoxy derivatives could be detected. The chromatographic separation was the same as described in Supplementary Fig. 9. The EIC trace of +*m*/*z* 231 covers $[M+Na]^+$ (**3**, **7**) and $[M+K]^+$ (**6**); the main ion of **6** is $[M+Na]^+$ *m*/*z* 215 (Supplementary Fig. 9). All the original LC-MS data were copied below.

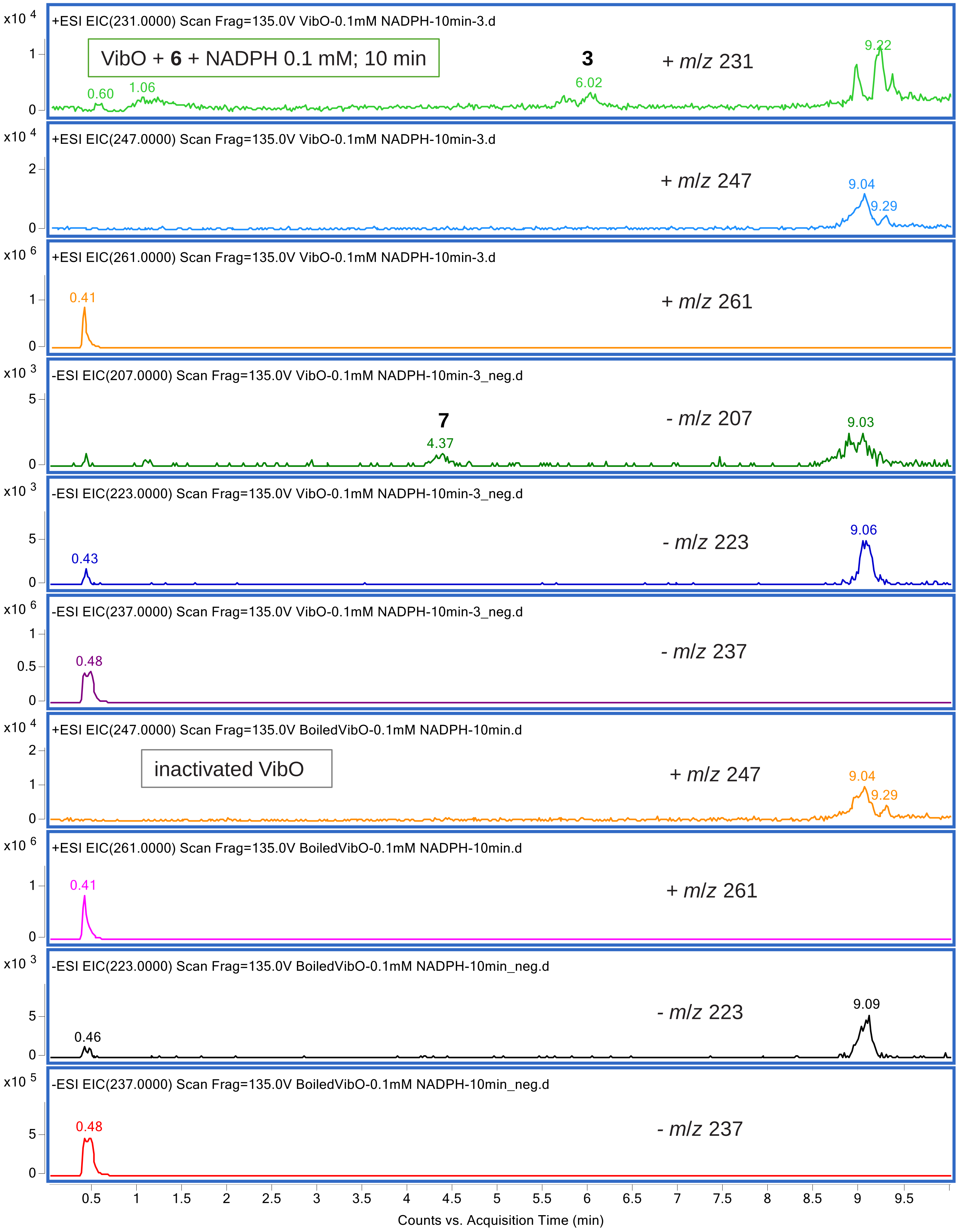

Continued on next page...

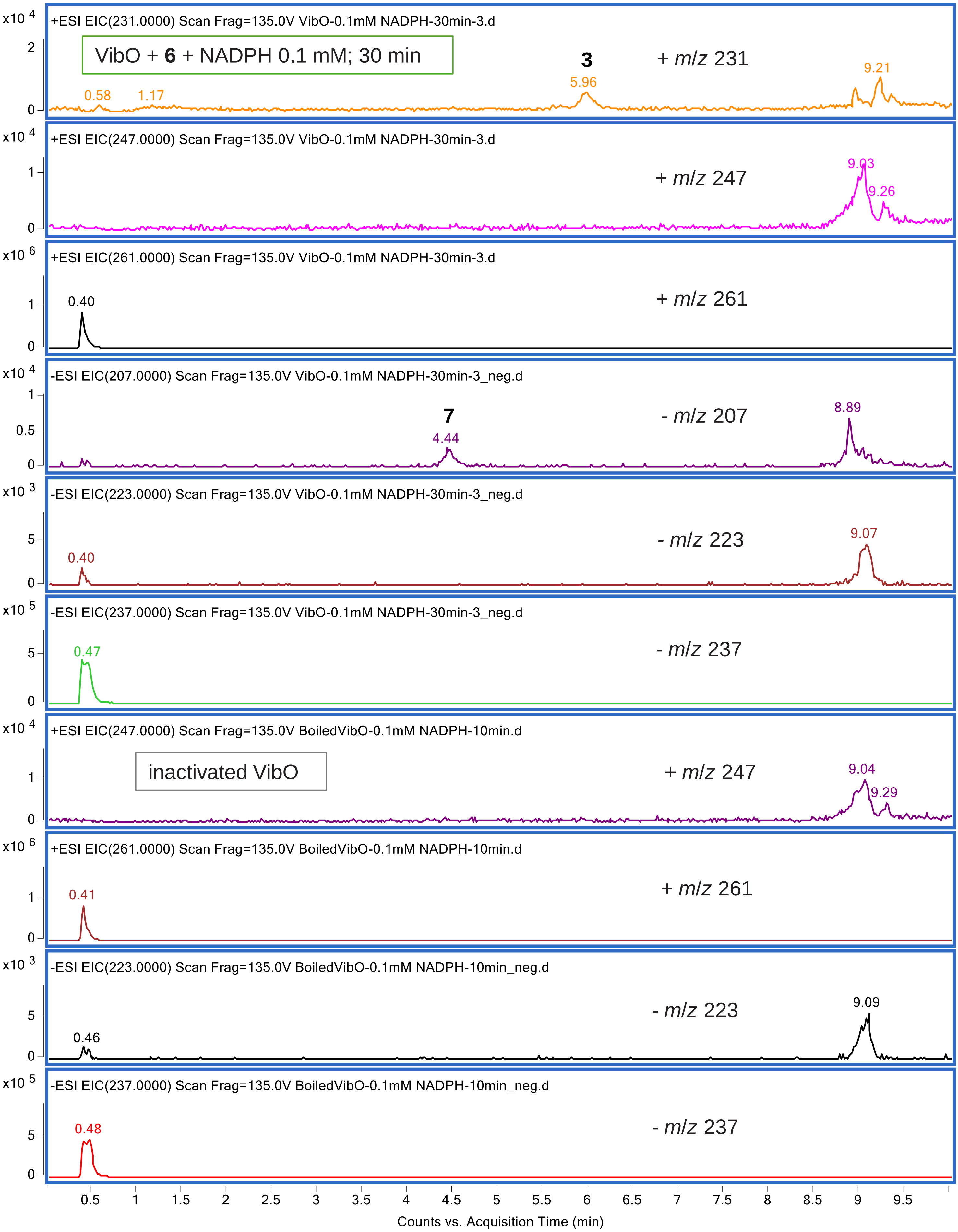

Continued on next page...

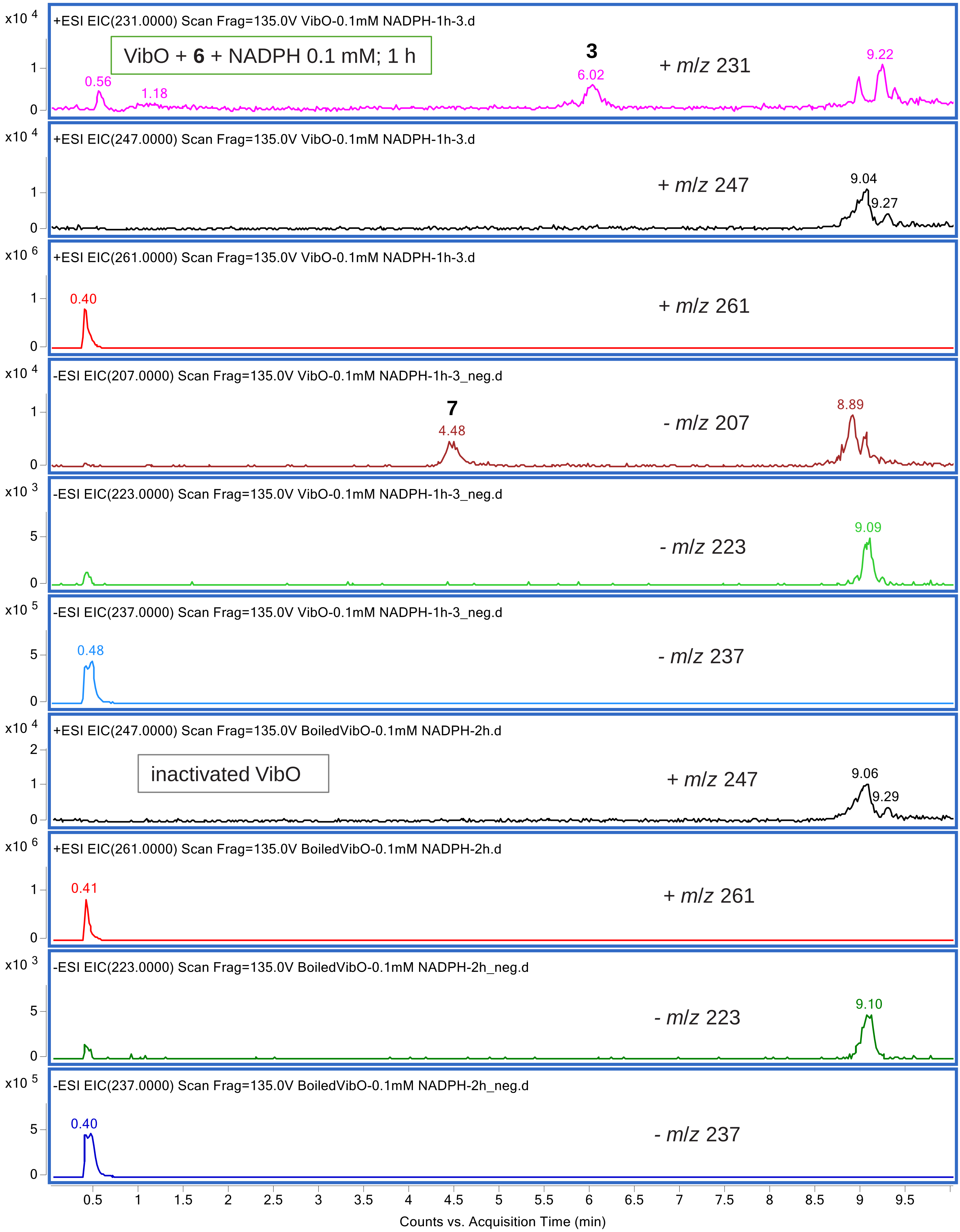

Continued on next page...

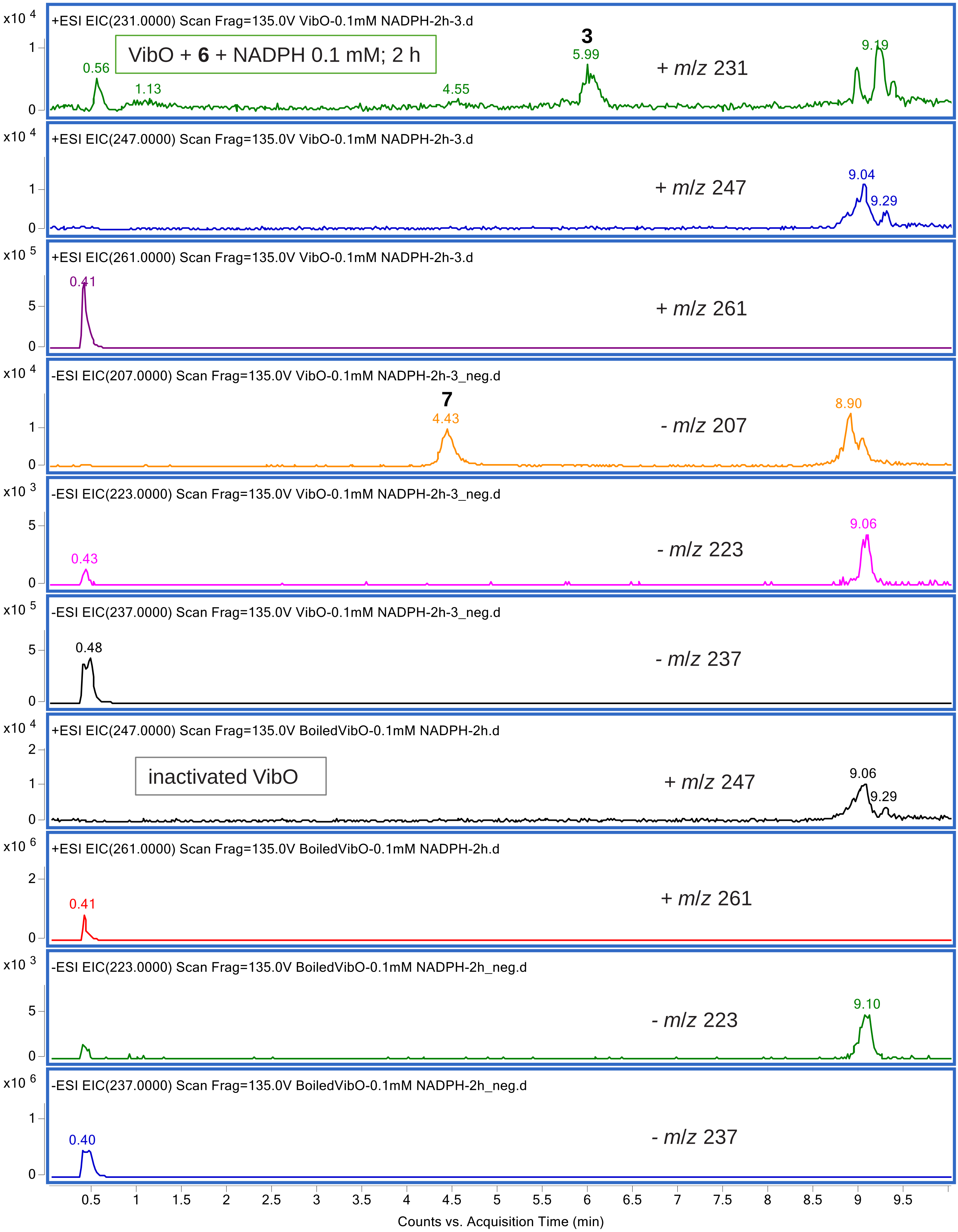

Continued on next page...

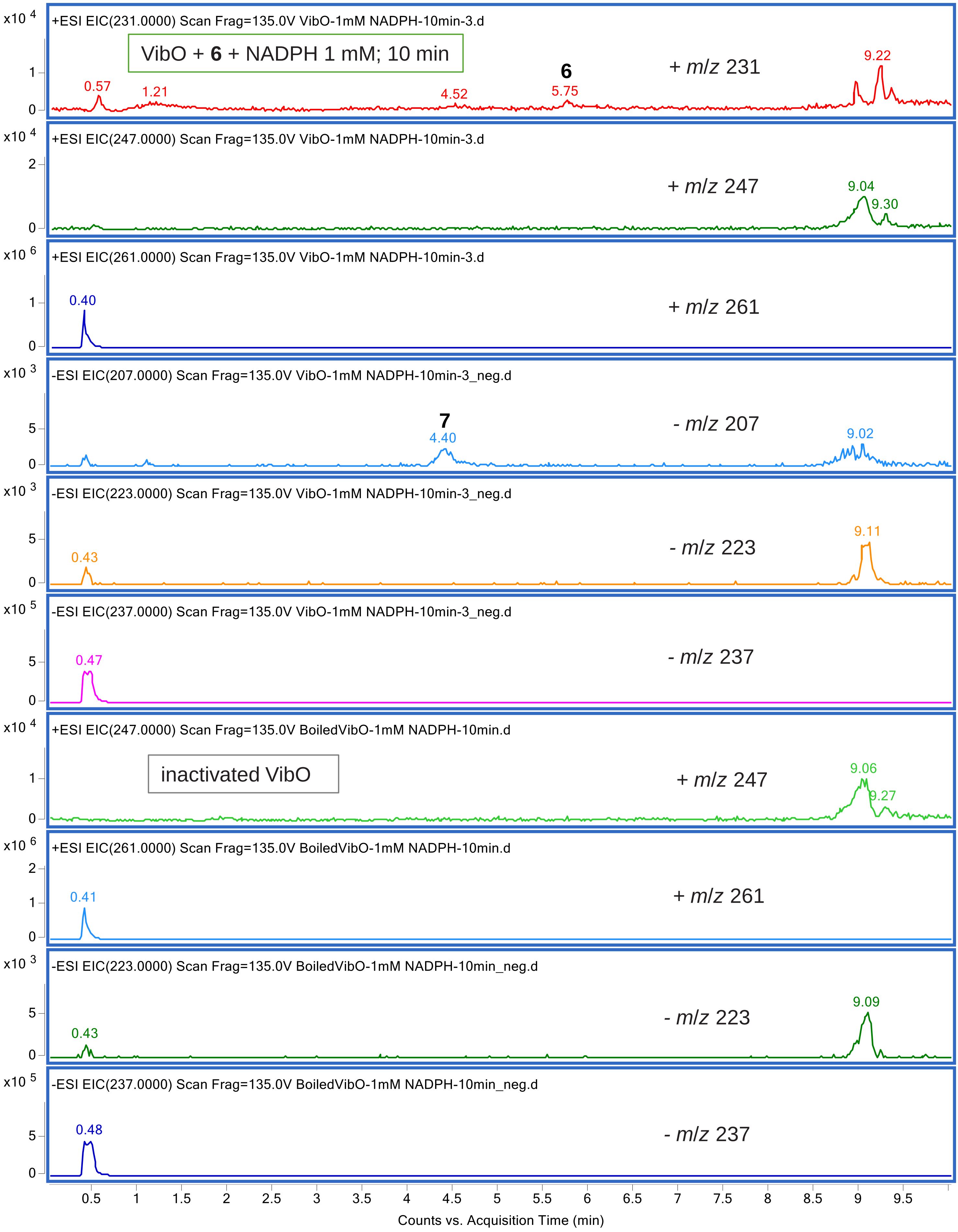

Continued on next page...

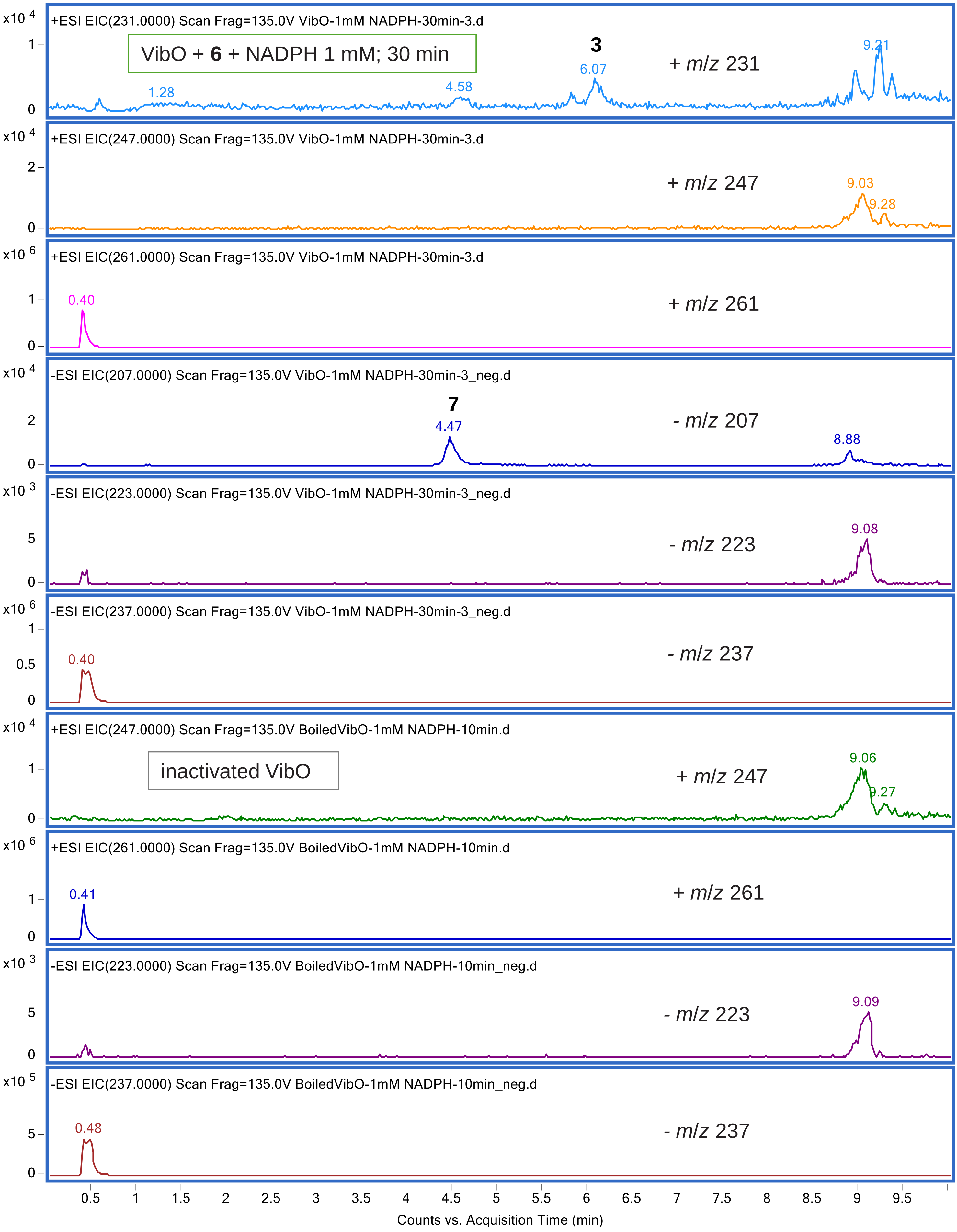

Continued on next page...

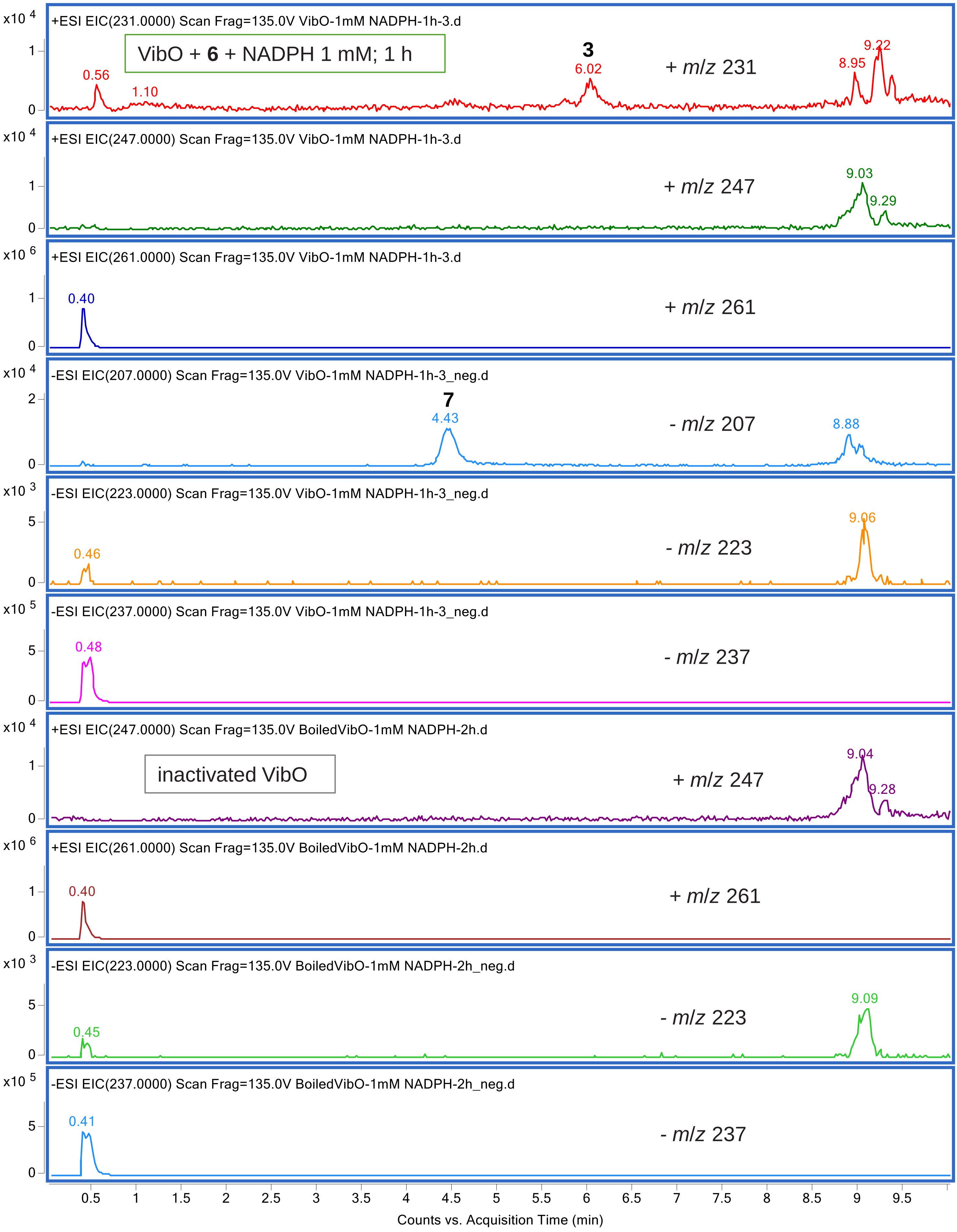

Continued on next page...

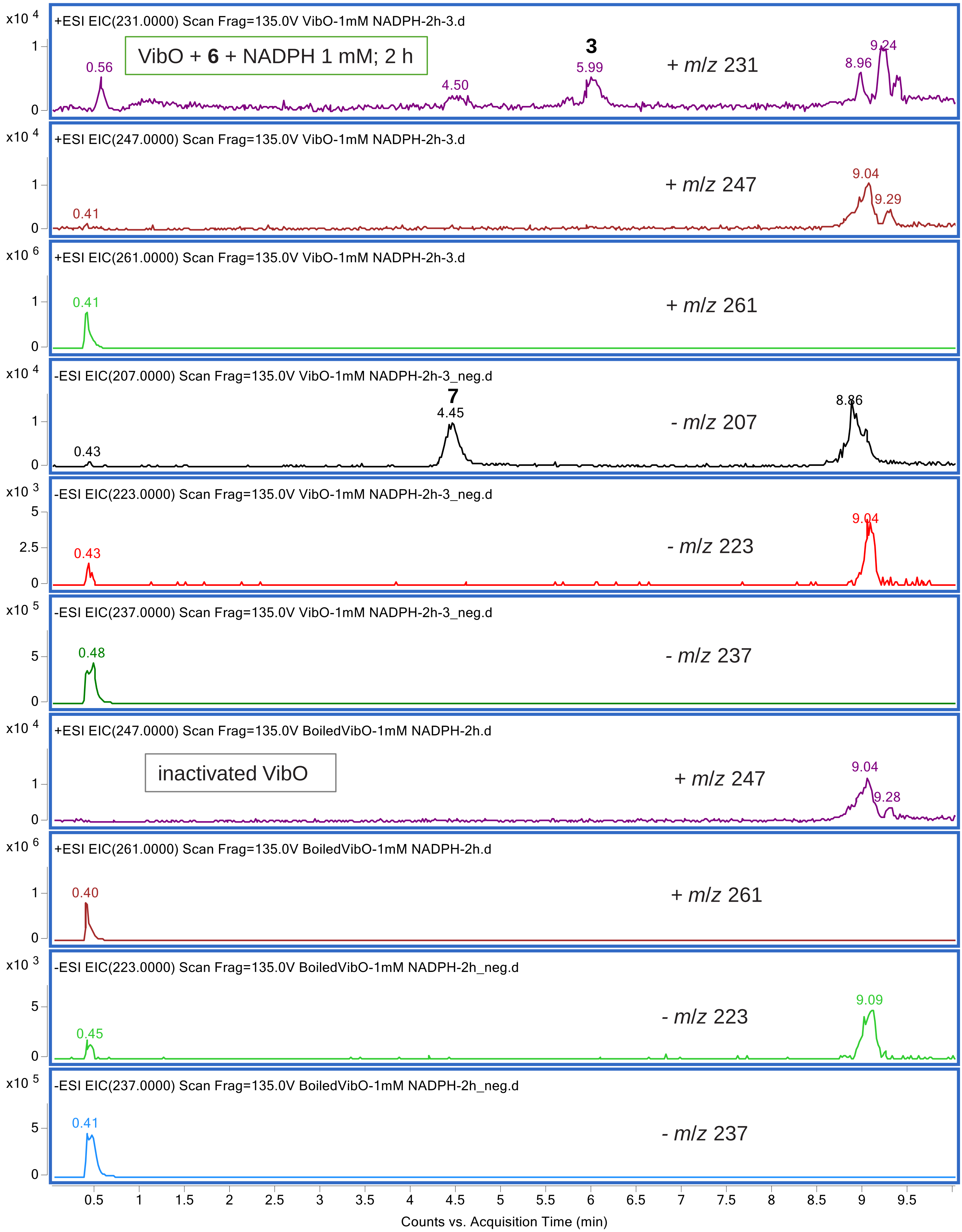

Continued on next page...

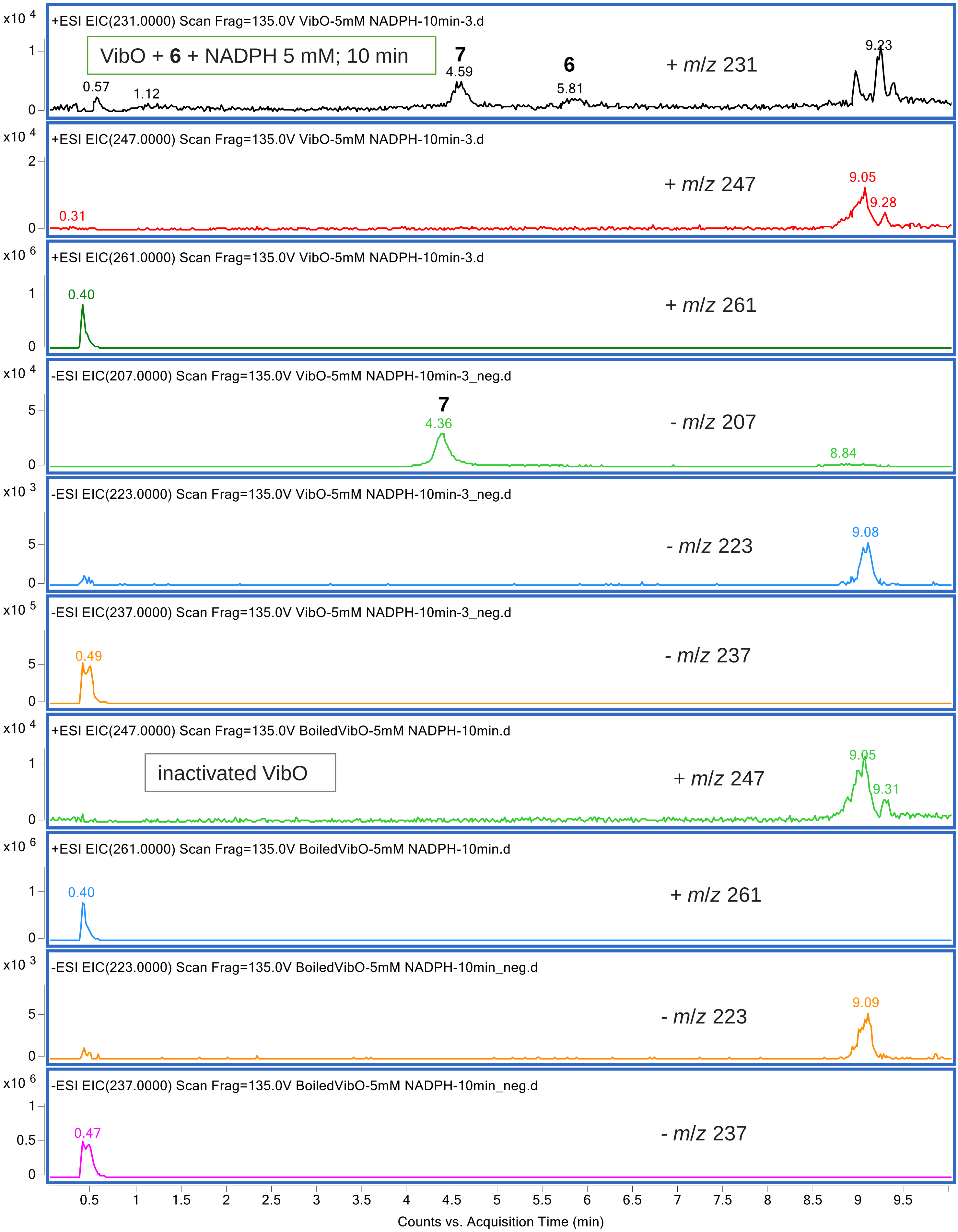

Continued on next page...

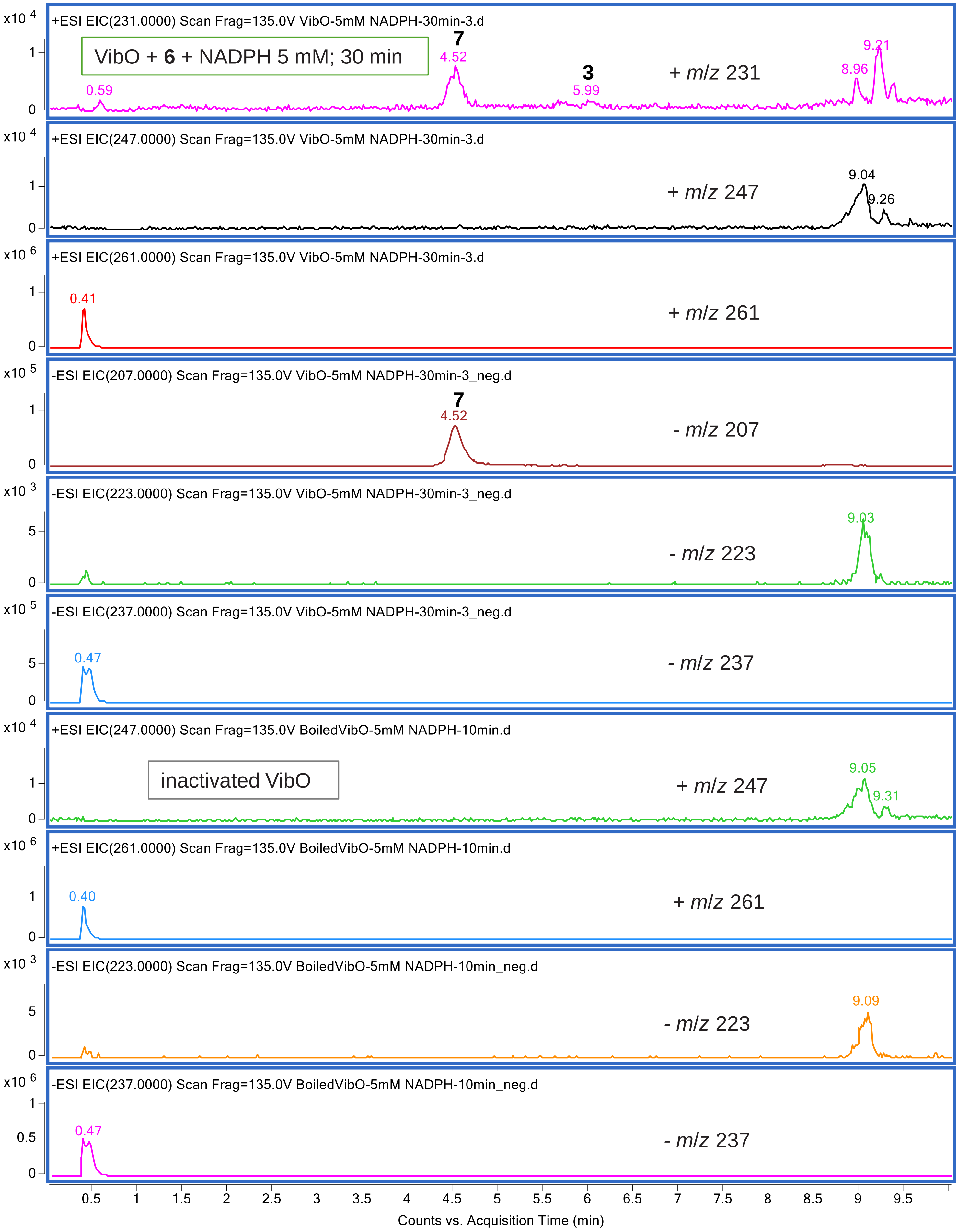

Continued on next page...

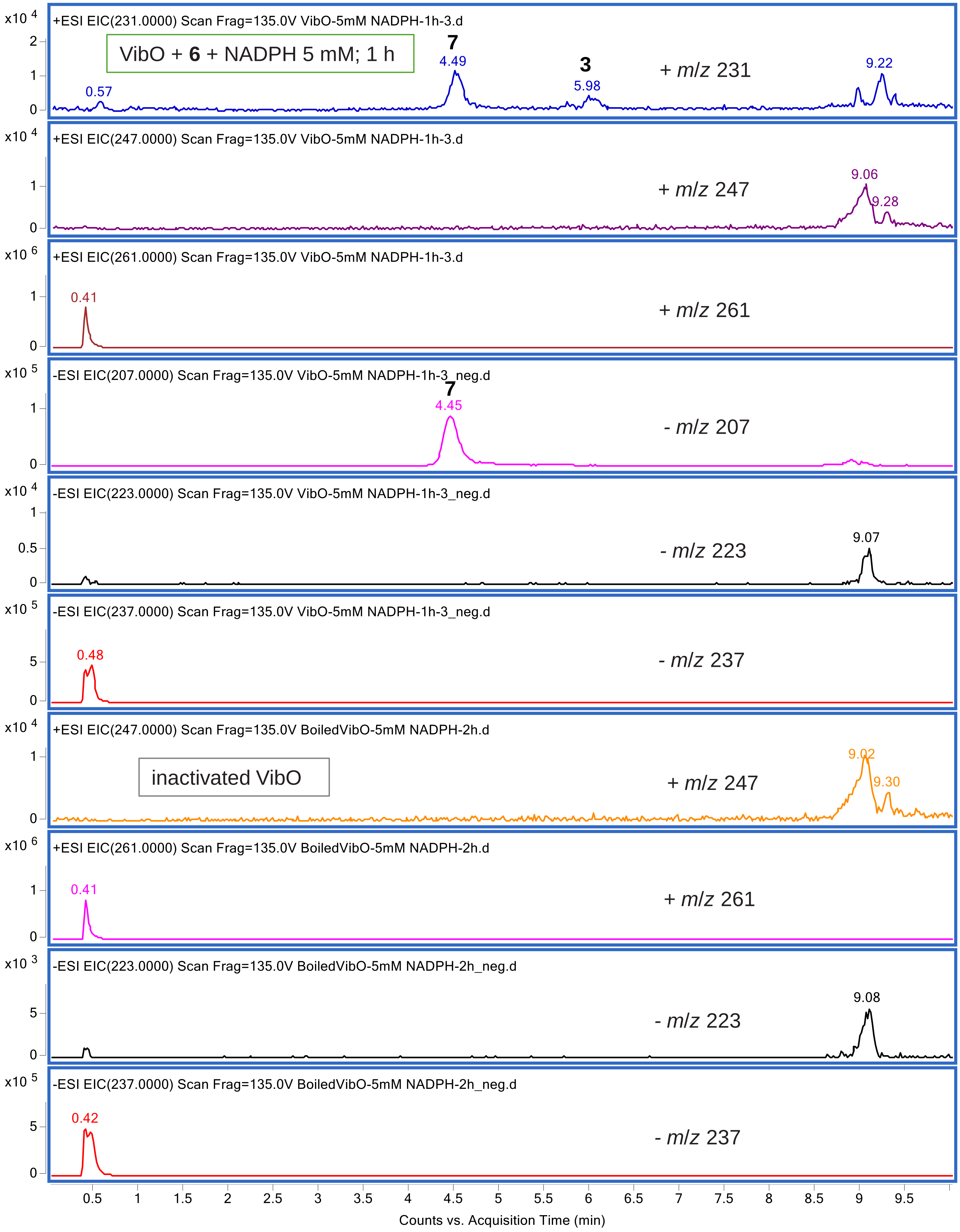

Continued on next page...

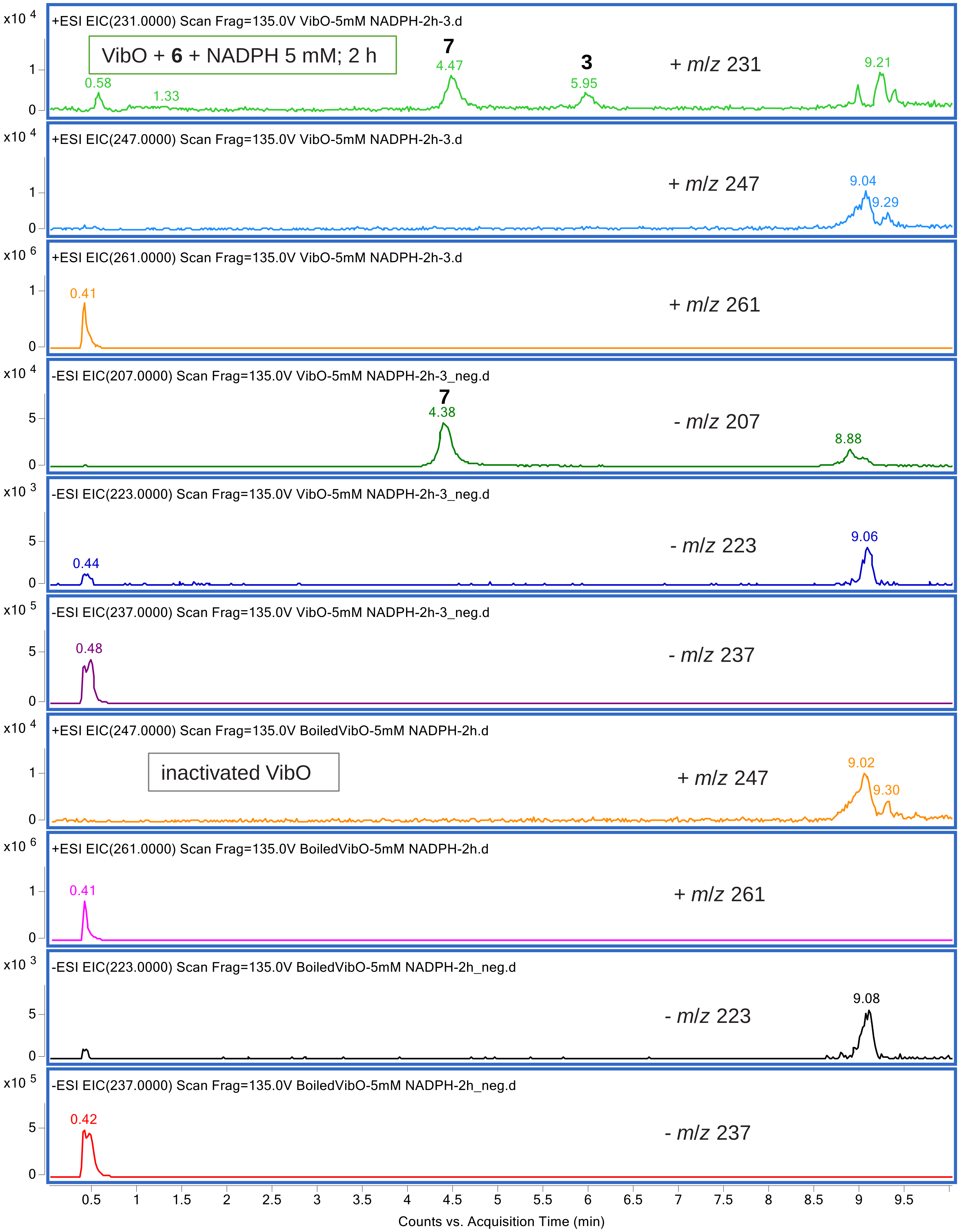
+ESI EIC(231.0000) Scan Frag=135.0V VibO-5mM NADPH-2h-3.d
VibO + 6 + NADPH 5 mM; 2 h
+ m/z 231
+ESI EIC(247.0000) Scan Frag=135.0V VibO-5mM NADPH-2h-3.d
+ m/z 247
+ESI EIC(261.0000) Scan Frag=135.0V VibO-5mM NADPH-2h-3.d
+ m/z 261
-ESI EIC(207.0000) Scan Frag=135.0V VibO-5mM NADPH-2h-3_neg.d
- m/z 207
-ESI EIC(223.0000) Scan Frag=135.0V VibO-5mM NADPH-2h-3_neg.d
- m/z 223
-ESI EIC(237.0000) Scan Frag=135.0V VibO-5mM NADPH-2h-3_neg.d
- m/z 237
+ESI EIC(247.0000) Scan Frag=135.0V BoiledVibO-5mM NADPH-2h.d
inactivated VibO
+ m/z 247
+ESI EIC(261.0000) Scan Frag=135.0V BoiledVibO-5mM NADPH-2h.d
+ m/z 261
-ESI EIC(223.0000) Scan Frag=135.0V BoiledVibO-5mM NADPH-2h_neg.d
- m/z 223
-ESI EIC(237.0000) Scan Frag=135.0V BoiledVibO-5mM NADPH-2h_neg.d
- m/z 237
Counts vs. Acquisition Time (min)

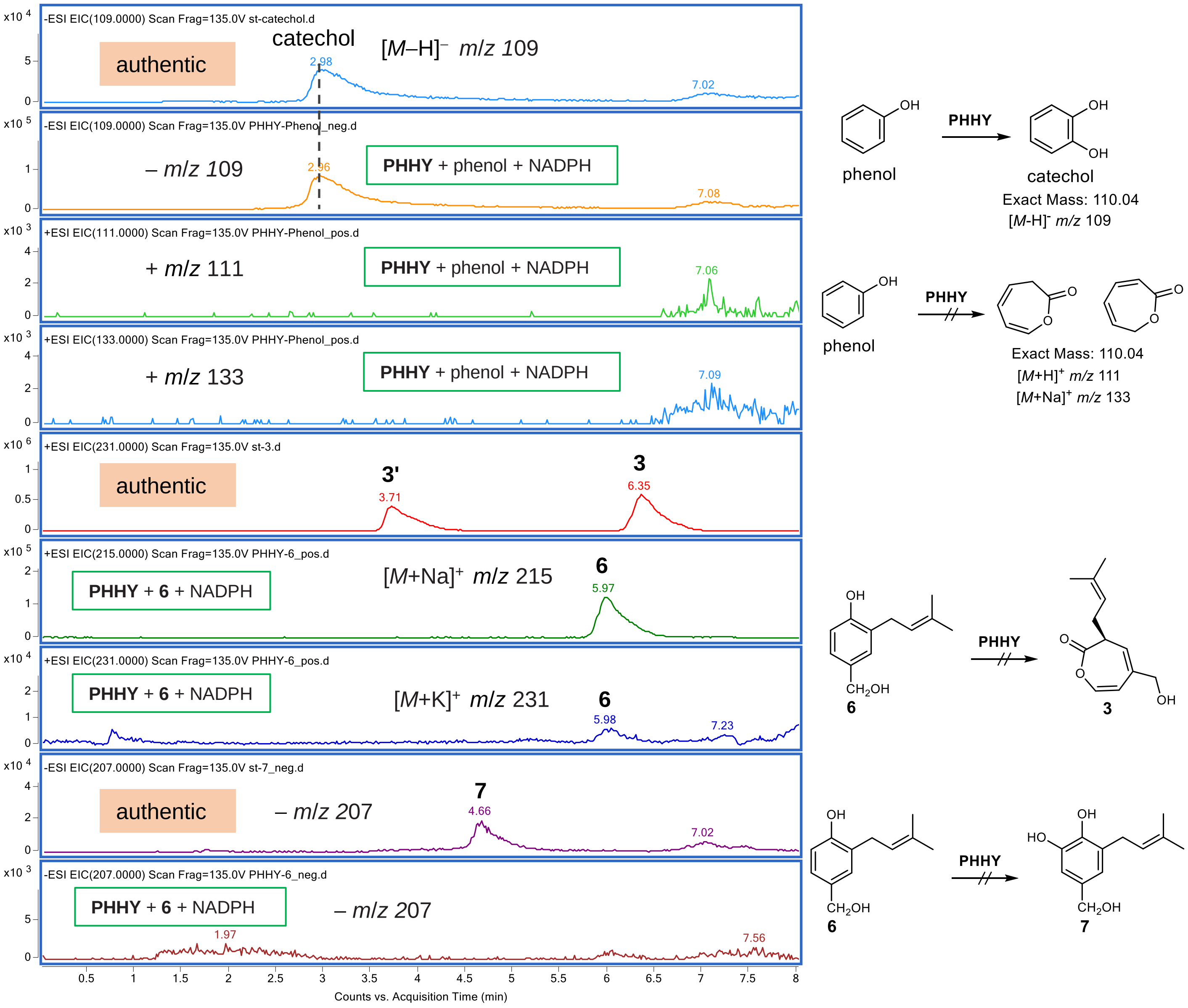

Supplementary Fig. 38. LC-MS analyses of PHHY incubating with 0.5 mM phenol or **6**; the chromatographic separation for catechol was performed with elution of 10% B over 4.9 min and 100% B over the next 3.1 min where A was H_2O and B was methanol. We synthesized the sequence of PHHY (PDB ID: 1PN0), expressed via pET28a(+) in *E. coli* BL21(DE3) and conducted enzyme assays as described for VibO (Supplementary Fig. 30). PHHY was observed to give catechol from phenol, but no detectable **3** or **7** production from **6** as substrate.

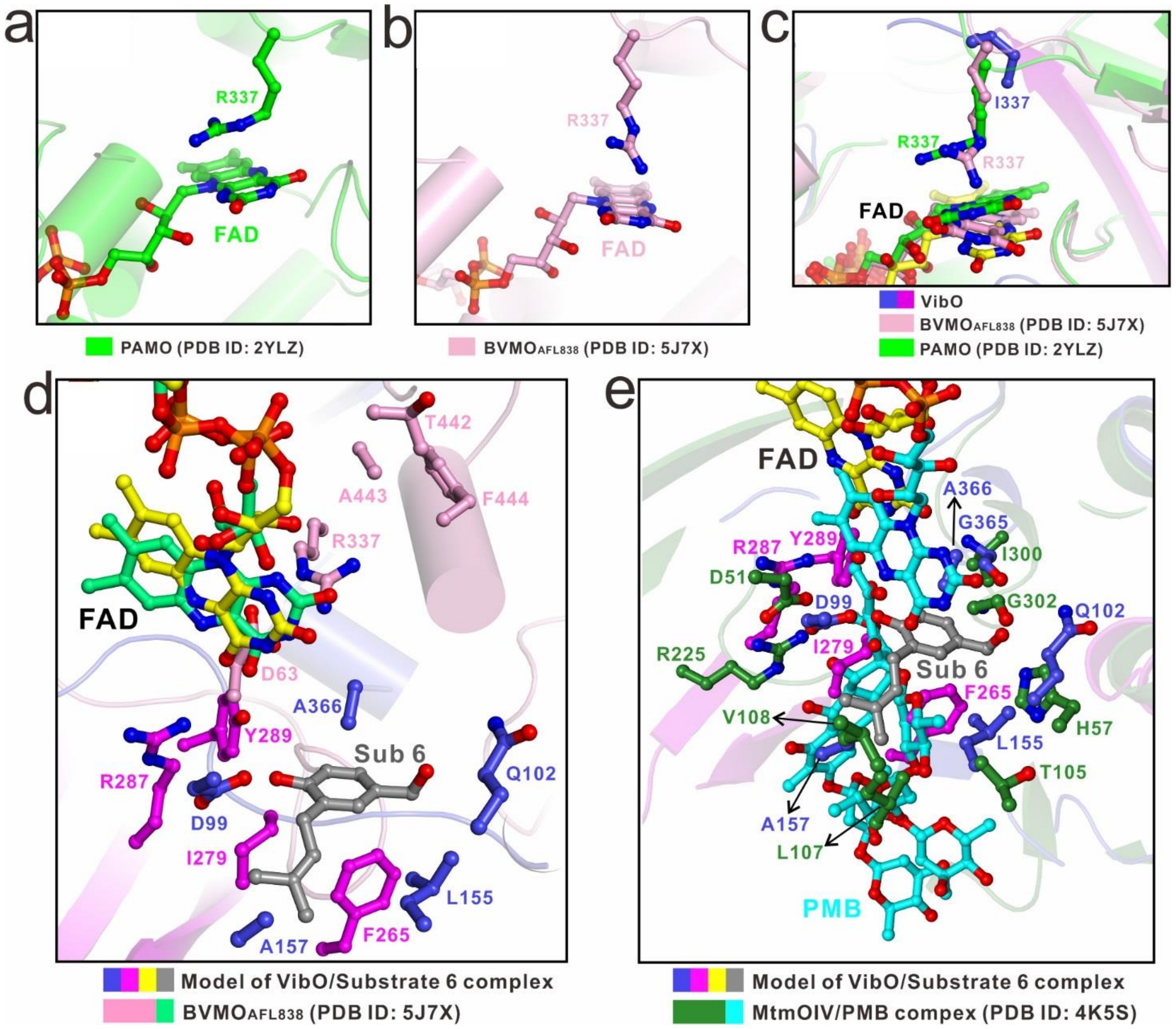

Supplementary Fig 39. Structural comparison analyses of the active site residues of VibO with relevant currently known Baeyer-Villiger oxygenases. (**a** and **b**) The ribbon-stick-ball representation showing the enlarged view of the key peroxyflavin stabilizing arginine residue of type-I BVMO PAMO (**a**), or $BVMO_{AFL838}$ (**b**) above the flavin ring of the co-factor FAD. (**c**) The ribbon-stick-ball representation showing the structural comparison of the key peroxyflavin stabilizing arginine residues of type-I BVMO PAMO and $BVMO_{AFL838}$ with the corresponding I337 residue of VibO. (**d** and **e**) The ribbon-stick-ball representation showing the detailed structural comparison of the key active site residues of VibO with that of $BVMO_{AFL838}$ (PDB ID: 5J7X) (**d**), or MtmOIV (PDB ID: 4K5S) (**e**). These structural analyses demonstrate that the active site residues and substrate-binding specificities of VibO are quite different from currently known fungal and bacterial Baeyer-Villiger oxygenases.

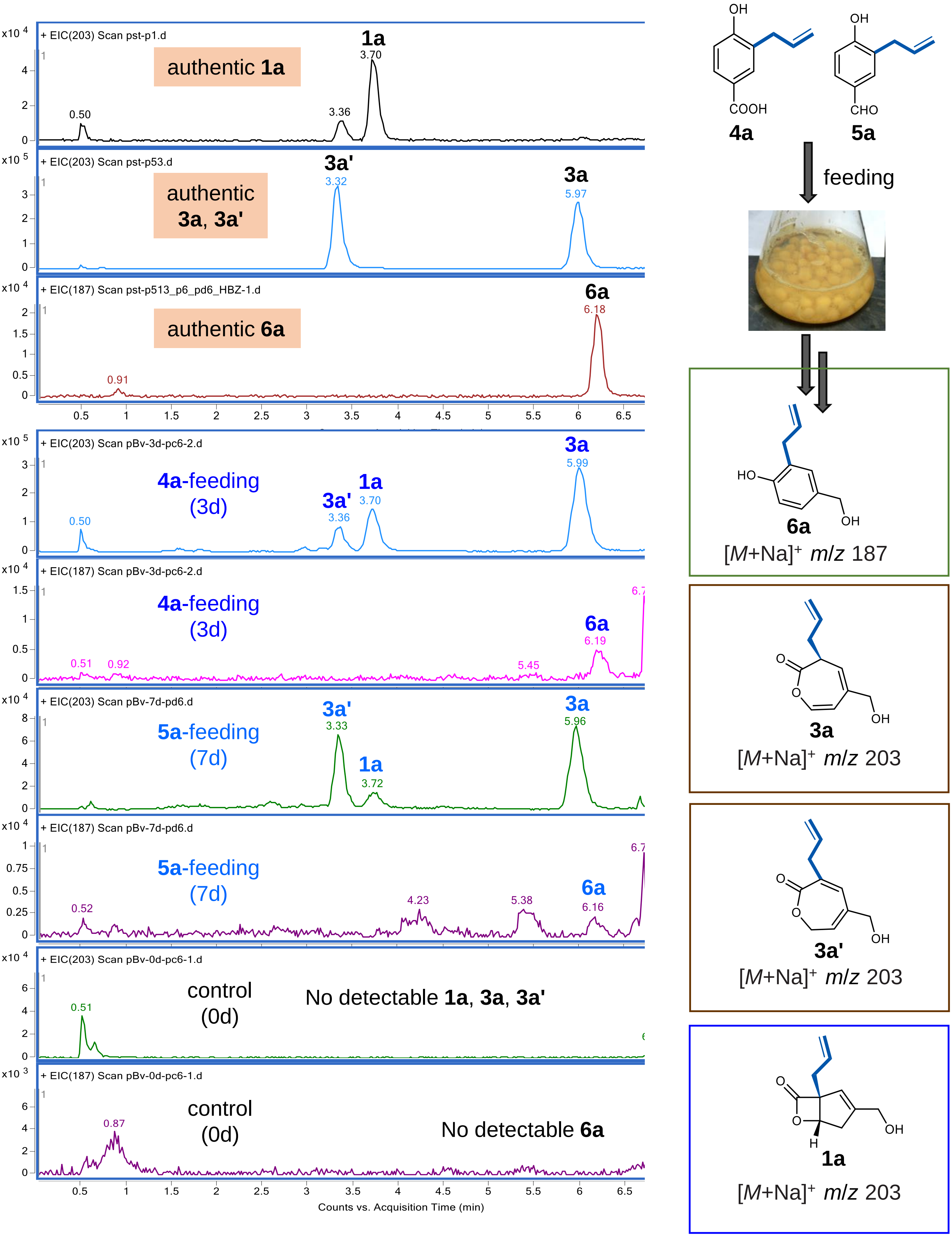

Supplementary Fig. 40. The original LC-MS data for feedings in Figure 6b. Once the accumulation of metabolite **3** in *B. vibrans* culture broth can be observed by LC-MS, the culture was fed with 1 mM of **4a** (or **5a**) and incubated for additional 3~7 days. The broth immediately after feeding was taken as control (0d). Chromatographic separation of **1a**, **3a**, **3a′**, **6a** was the same as described in Supplementary Fig. 1.

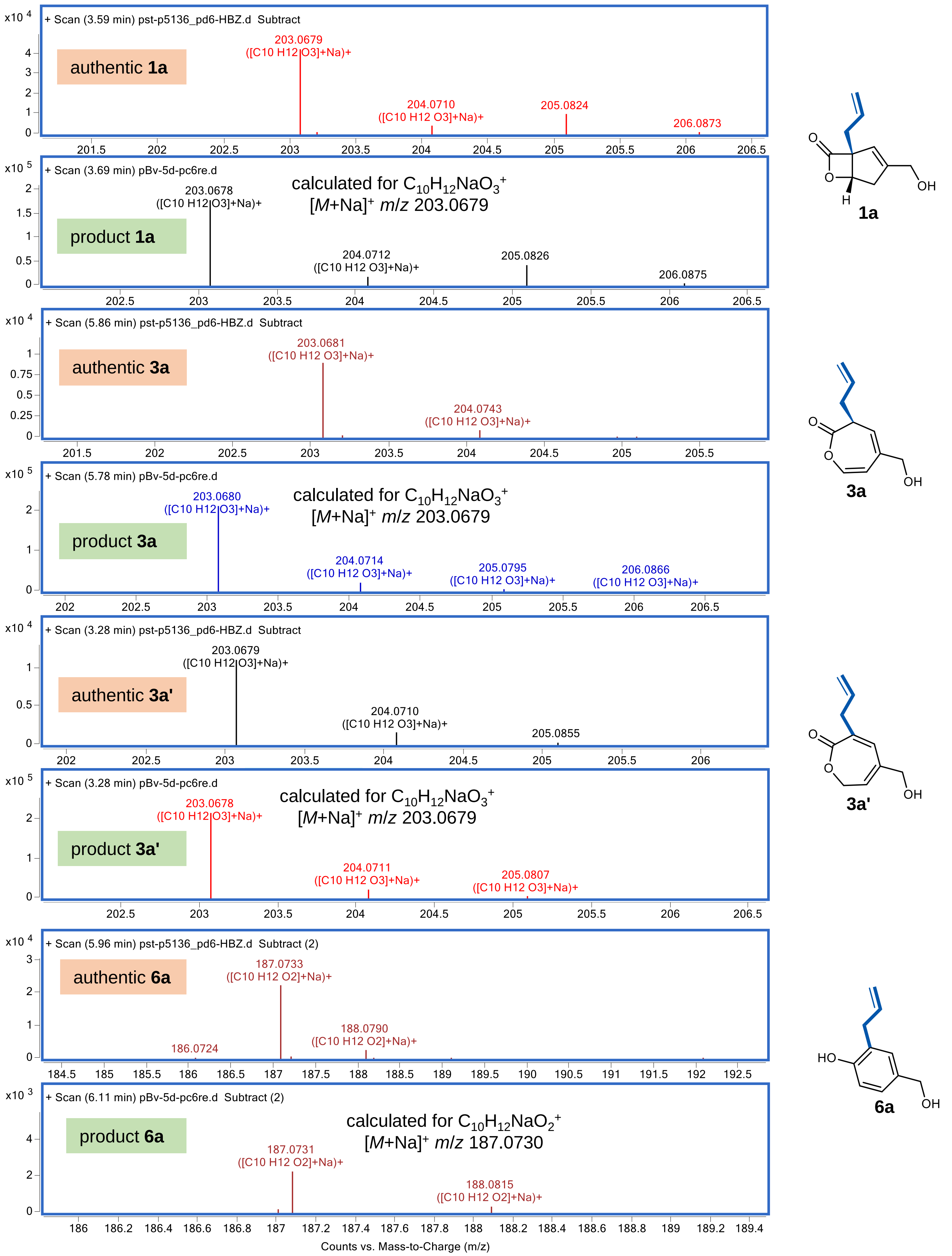

Supplementary Fig. 41. The original HRMS data for Figure 6c.

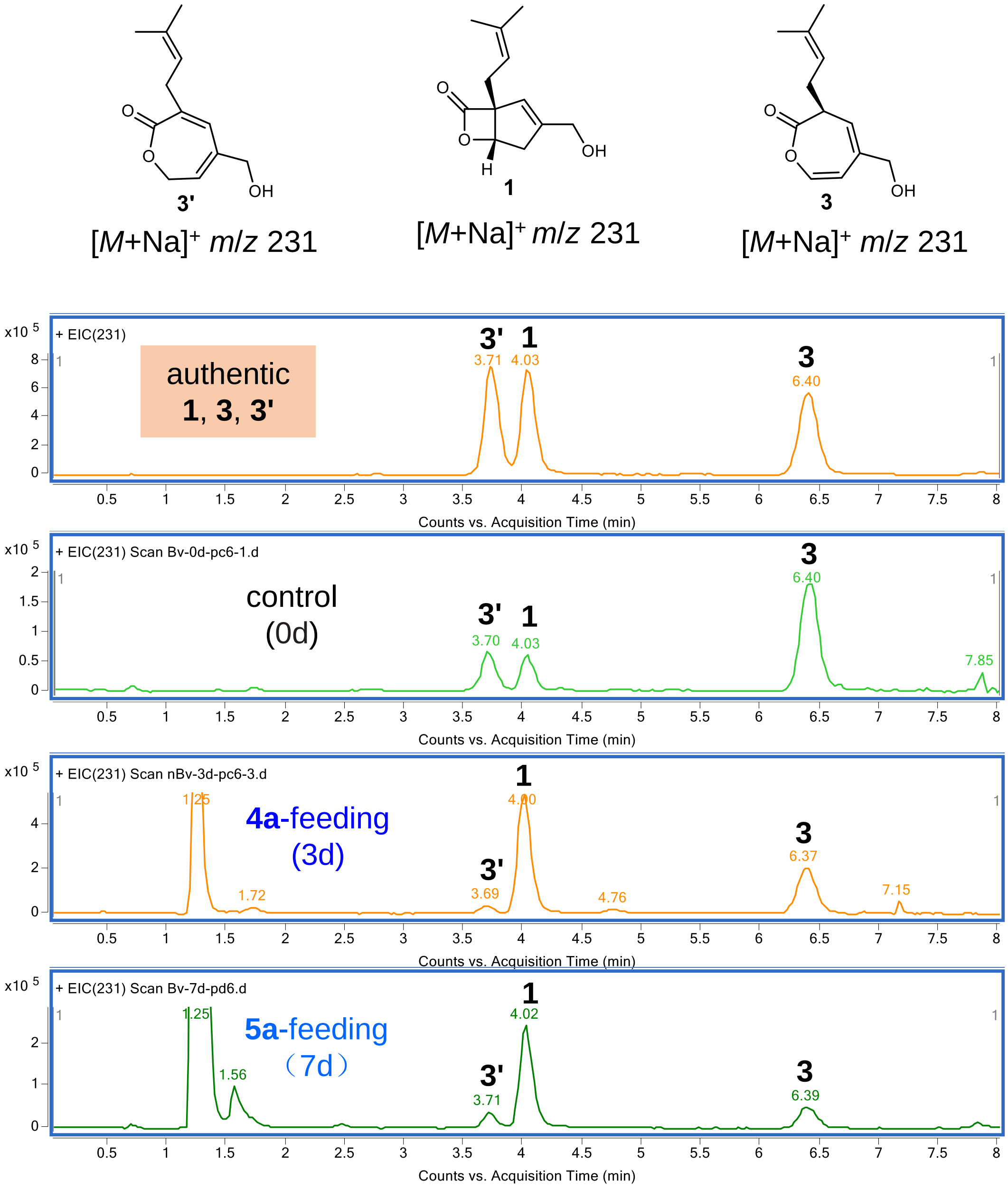

Supplementary Fig. 42. LC-MS analyses of native metabolites 1, 3, **and** 3′ **in feedings**. Chromatographic separation was the same as described in Supplementary Fig. 4.

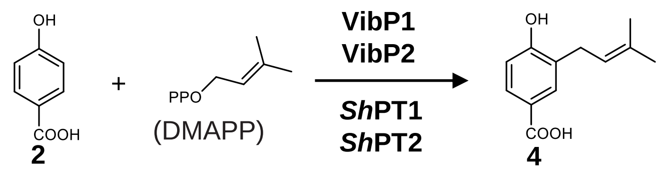

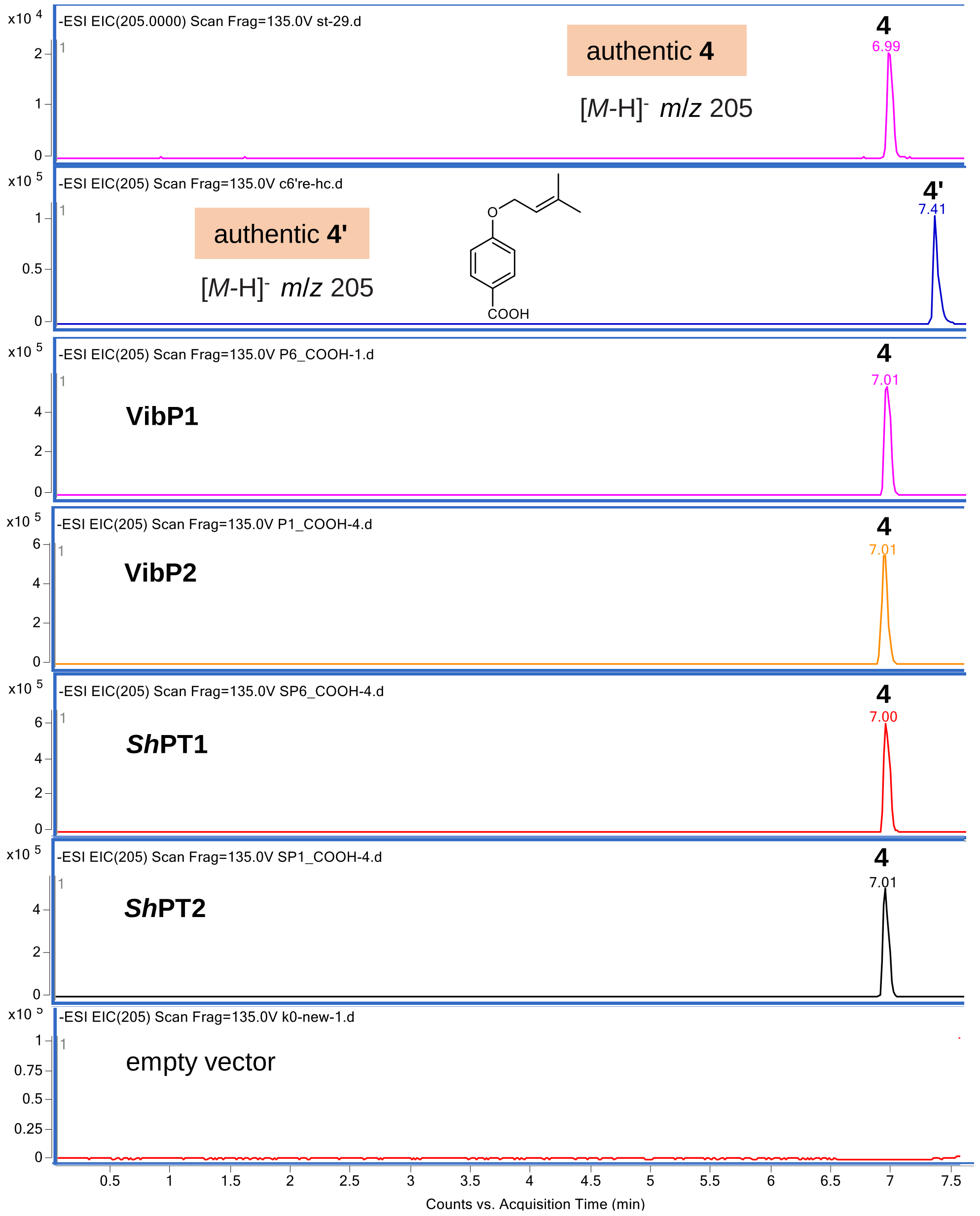

Supplementary Fig. 43. The original LC-MS data for enzymatic production of 4 **from 4-hydroxybenzoate** (2) **in Figure 7a**. The *E. coli* strain containing pET28a(+) without target sequences (denoted by "empty vector") was used as control. Chromatographic separation was performed with elution of 43% B over 4.9 min and 100% B over the next 3.1 min where A was 0.1% formic acid and B was methanol. The mass spectrometer was run in negative ionization mode.

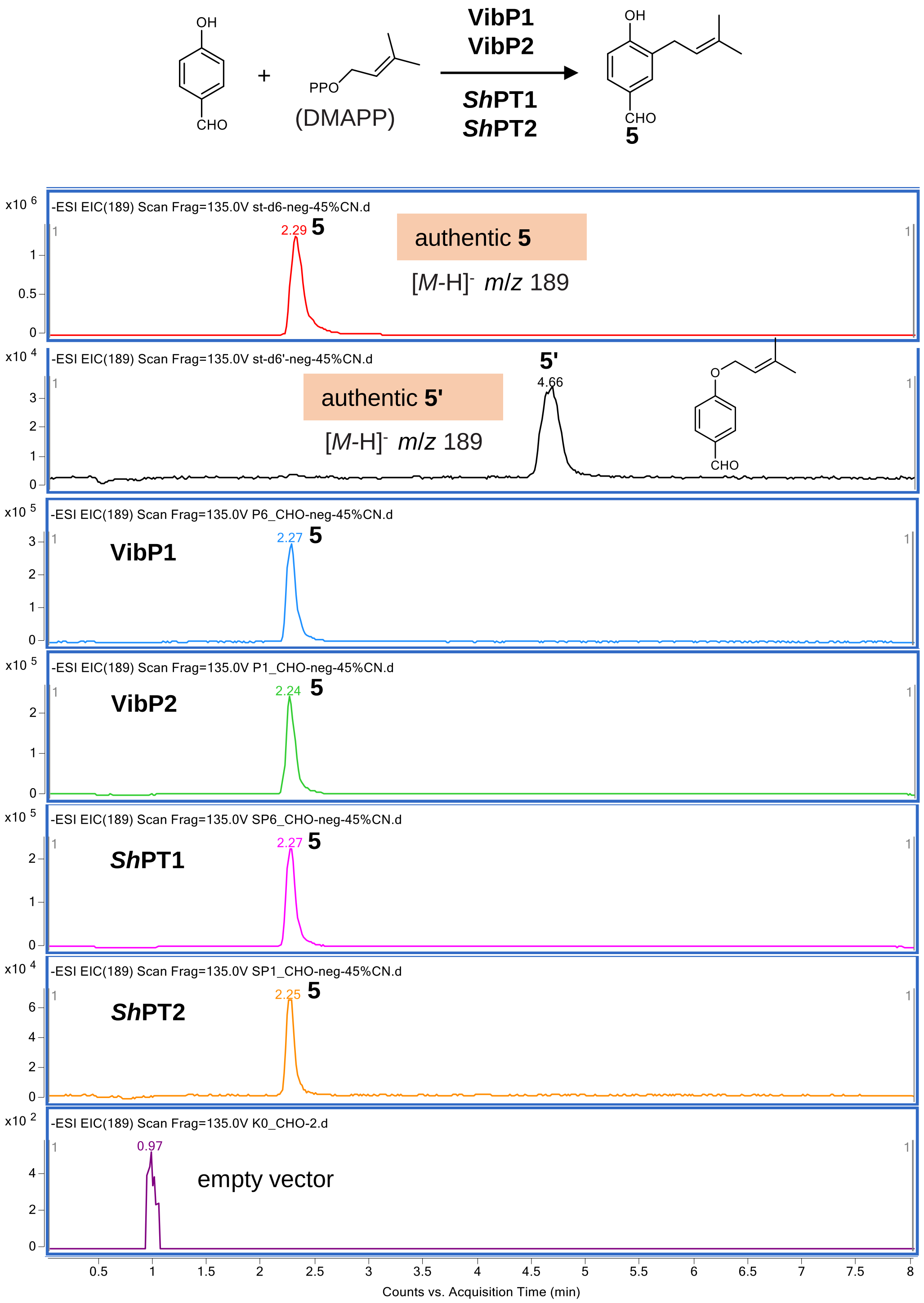

Supplementary Fig. 44. The original LC-MS data for enzymatic production of 5 **from 4-hydroxybenzaldehyde in Figure 7a**. The *E. coli* strain containing pET28a(+) without target sequences (denoted by "empty vector") was used as control. Chromatographic separation was performed with elution of 45% B over 6 min and 100% B over the next 3 min where A was H_2O and B was acetonitrile. The mass spectrometer was run in negative ionization mode.

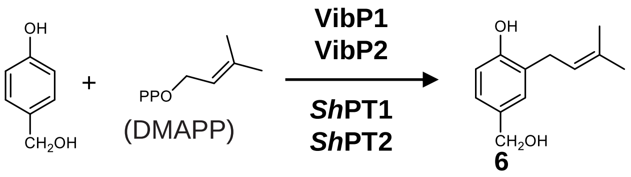

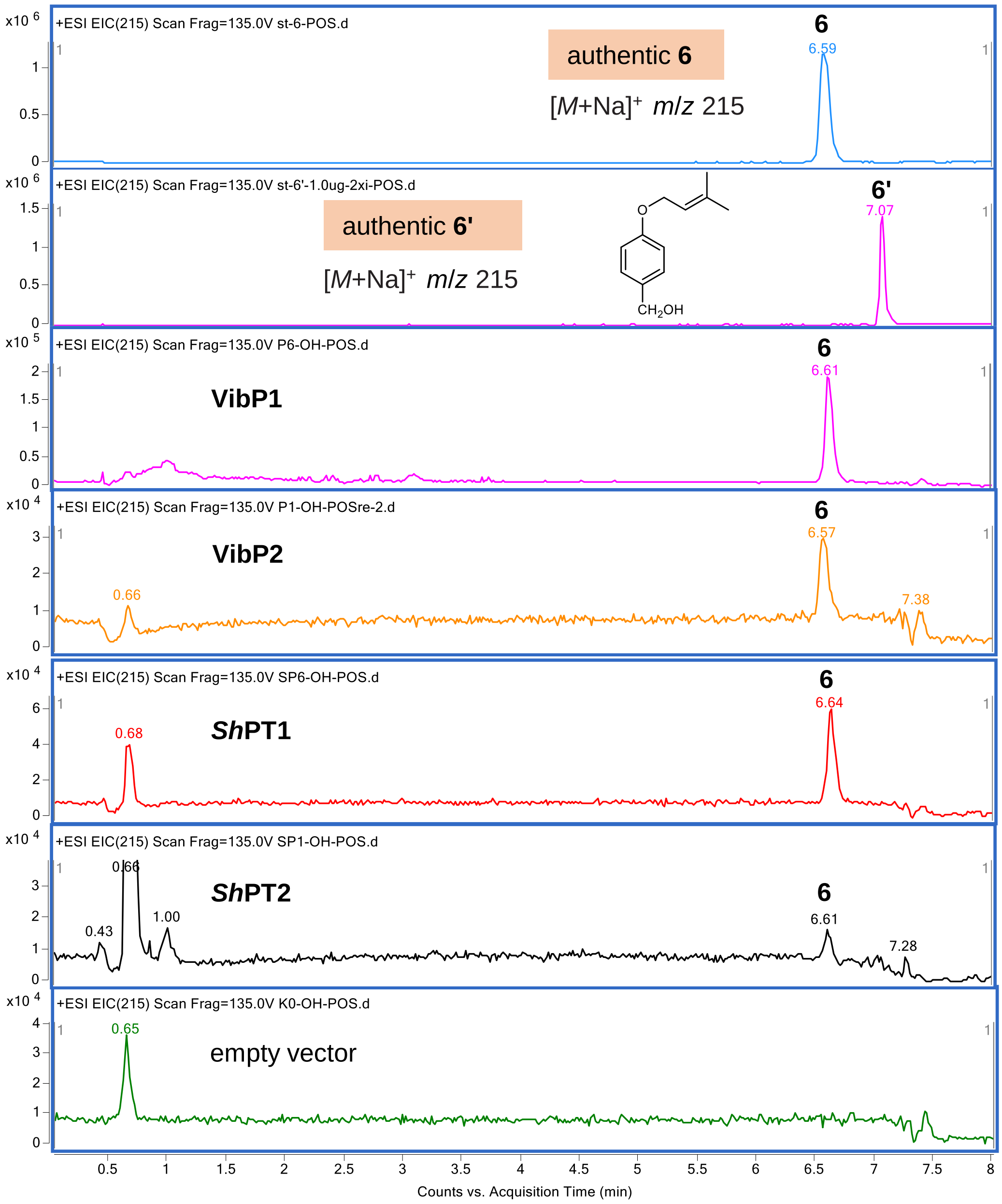

Supplementary Fig. 45. The original LC-MS data for enzymatic production of 6 **from 4-hydroxybenzyl alcohol in Figure 7a**. The *E. coli* strain containing pET28a(+) without target sequences (denoted by "empty vector") was used as control. The chromatographic separation was the same as described in Supplementary Fig. 4. To recognize **6** from **6′**, the mass spectrometer was run in positive ionization mode as **6′** was barely detected under negative mode.

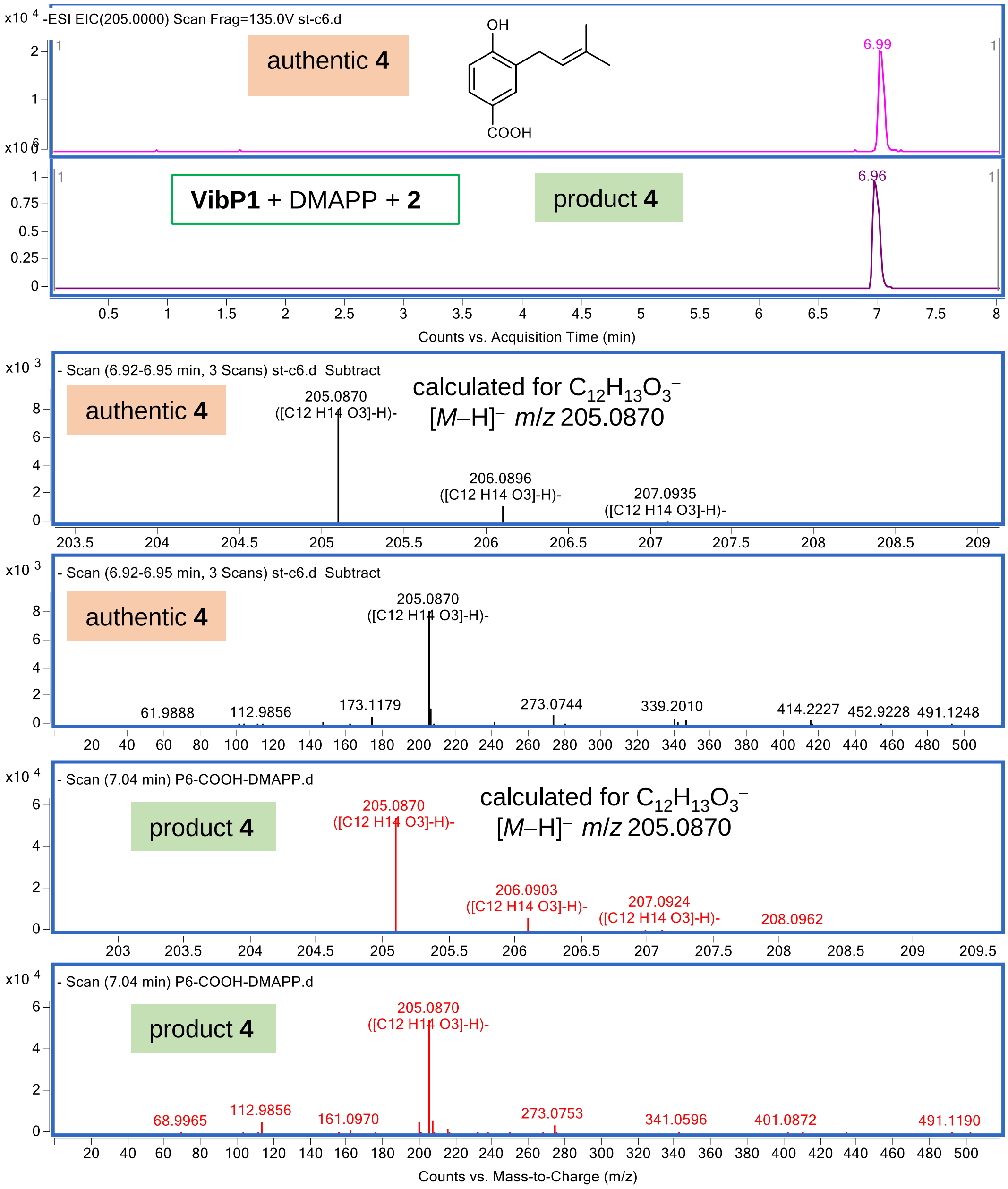

Supplementary Fig. 46. The original HRMS data for compound 4 in Figure 7d.

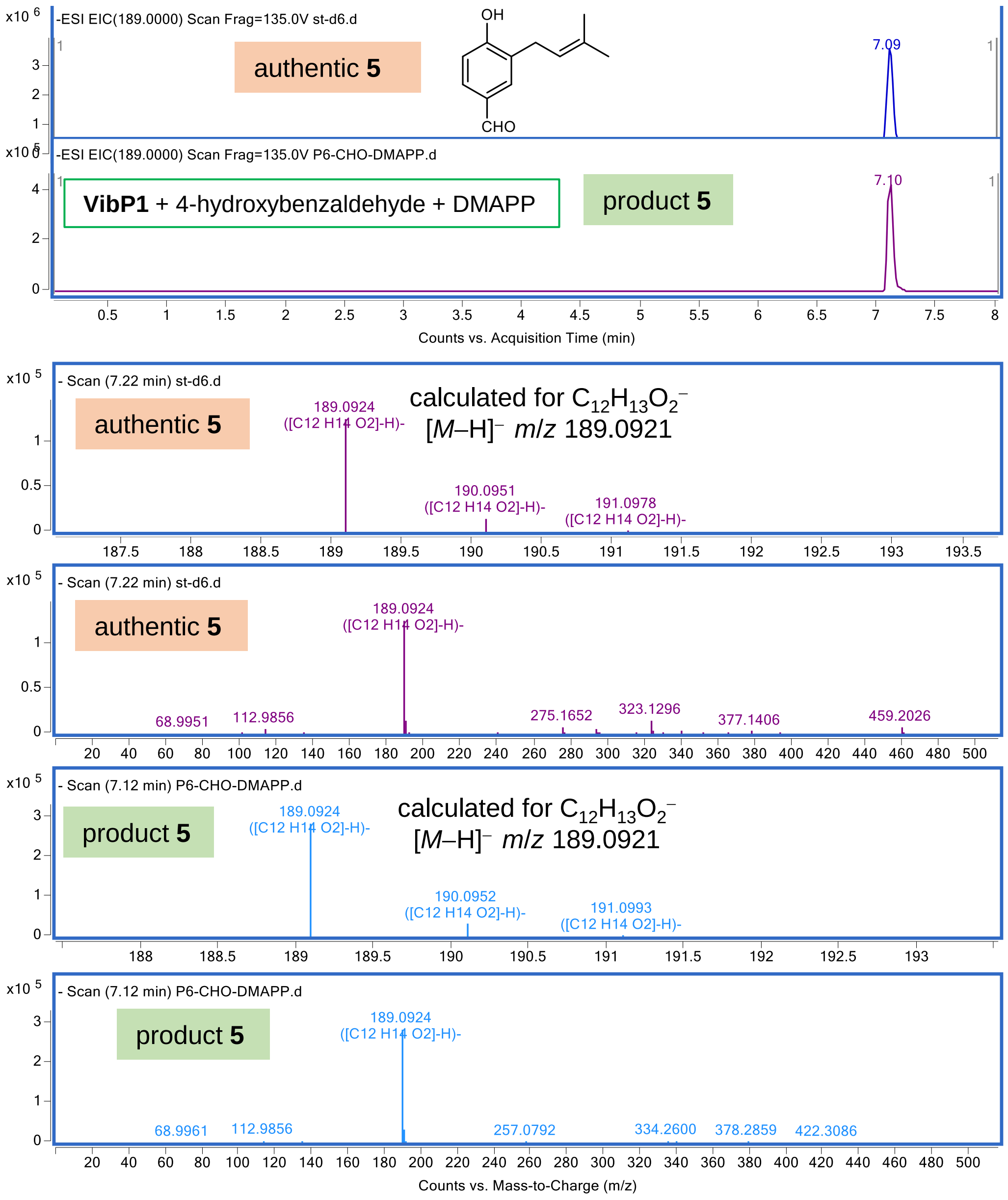

Supplementary Fig. 47. The original HRMS data for compound 5 **in Figure 7d**. Chromatographic separation was performed with elution of 43% B over 4.9 min and 100% B over the next 3.1 min where A was H_2O and B was methanol. The mass spectrometer was run in negative ionization mode.

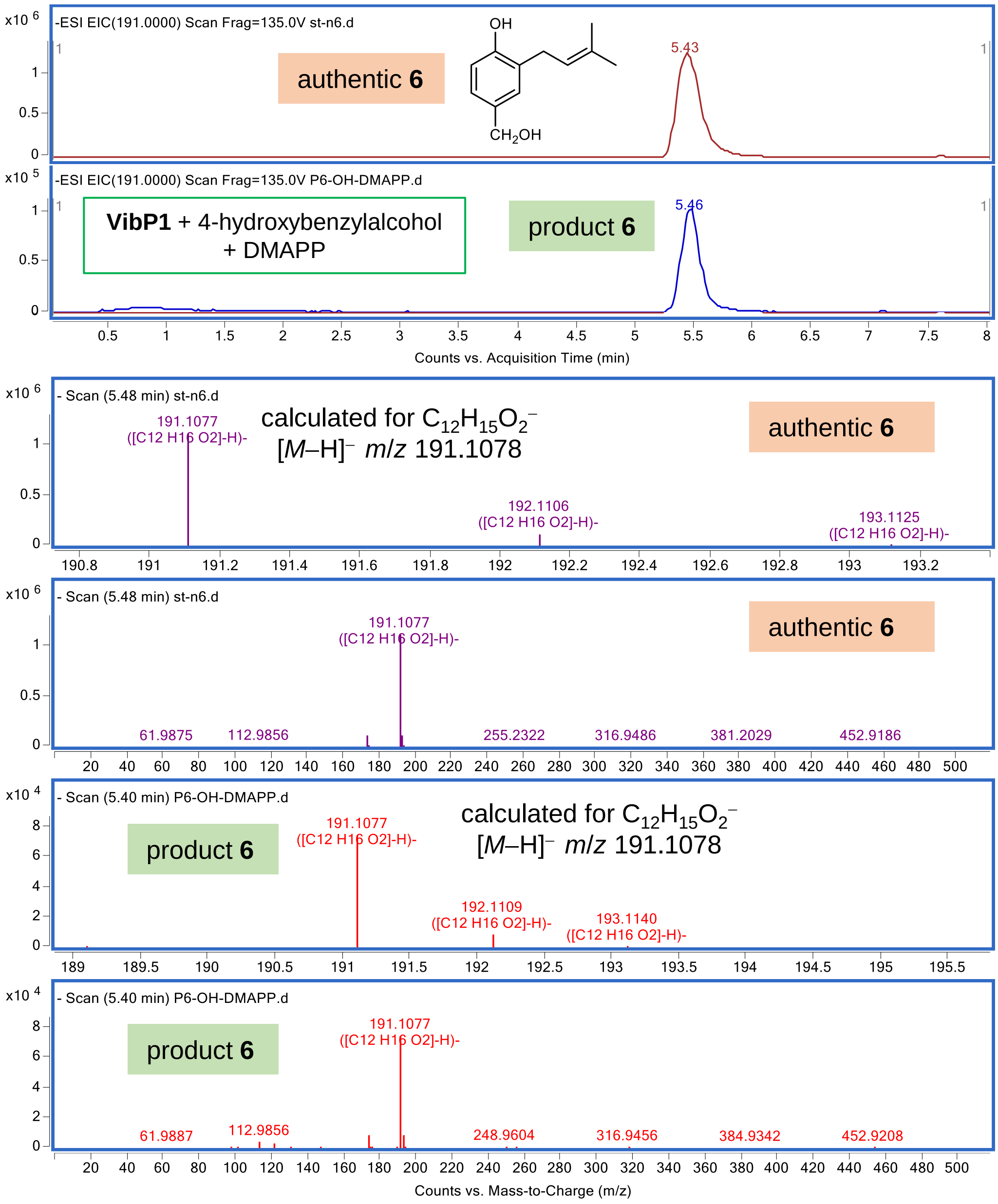

Supplementary Fig. 48. The original HRMS data for compound 6 **in Figure 7d**. The chromatographic separation was the same as described in Supplementary Fig. 4. The mass spectrometer was run in negative ionization mode.

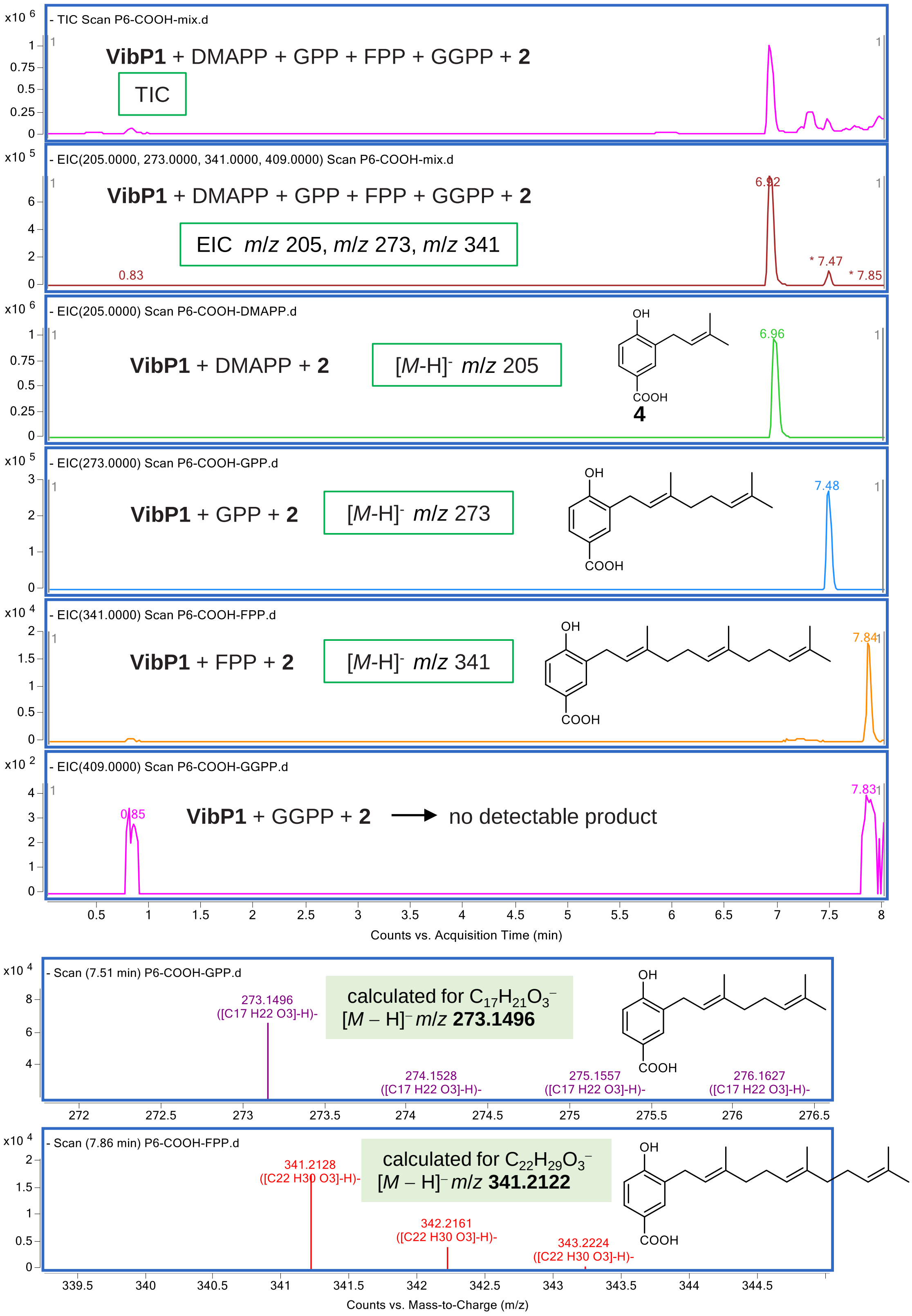

Supplementary Fig. 49. LC-HRMS analyses of products of VibP1 and prenyl pyrophosphates with different chain lengths. Membrane extracts (40 µL) of VibP1 were incubated with 4-hydoxybenzoate (**2**, 0.5 mM) and mixture of DMAPP / GPP / FPP / GGPP (each 0.2 mM), in parallel with VibP1 with individual DMAPP or GPP or FPP or GGPP at 0.2 mM, at 30°C for 3h.

Chromatographic separation was performed with elution of 43% B over 4.9 min and 100% B over the next 3.1 min where A was 0.1% formic acid and B was methanol. C_{10} and C_{15} prenylated products were tentatively identified by HRMS.

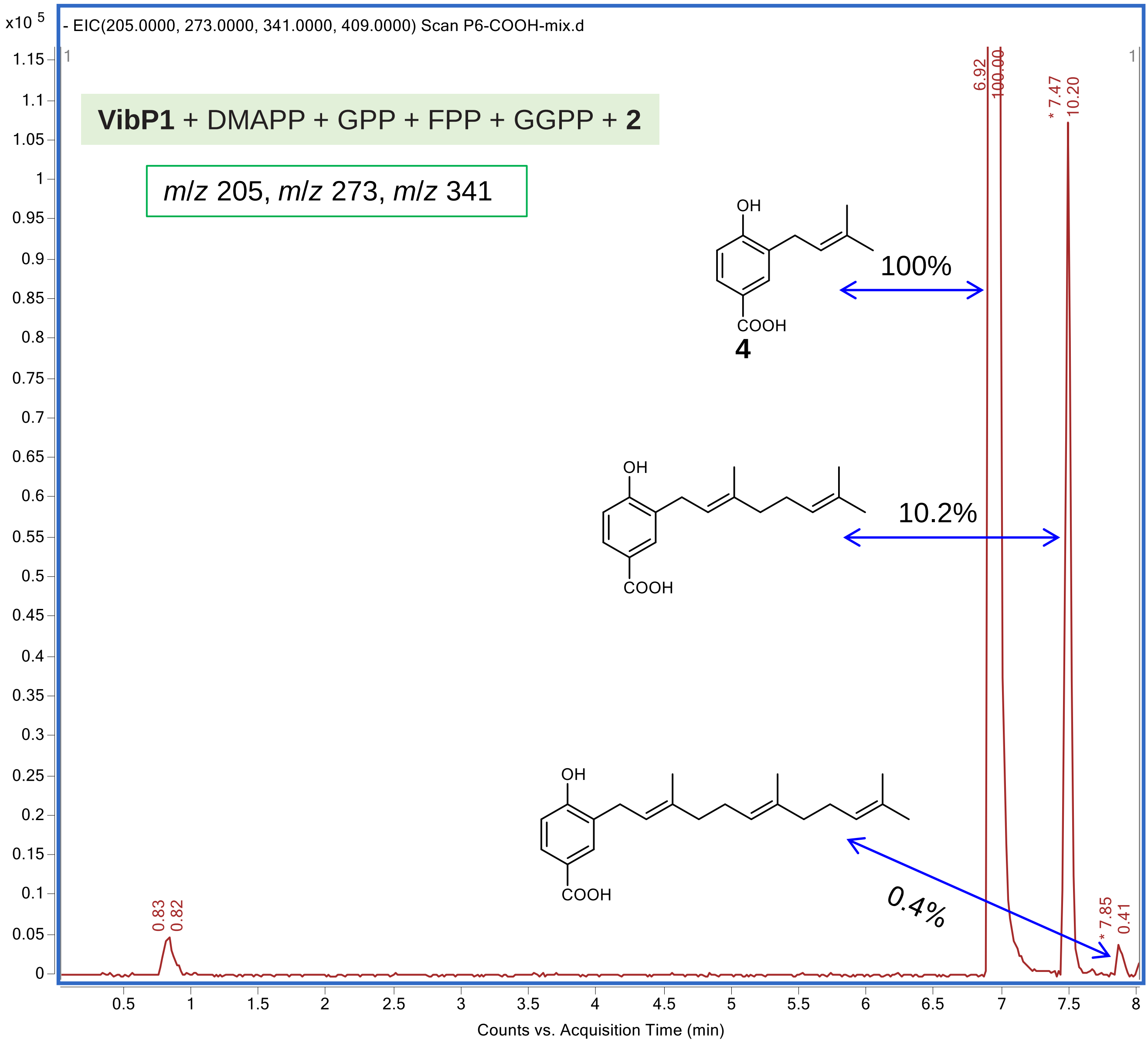

Supplementary Fig. 50. Peak area ratios of putative products by VibP1 with mixture of DMAPP / GPP / FPP / GGPP shown in Supplementary Fig. 49.

Seq->	VibP1	VibP2	ShPT1	ShPT2	BisJ	PesD	UbiA	XimB	LePGT1	BvPT	BypB
VibP1	ID										
VibP2	0.671	ID									
ShPT1	0.929	0.674	ID								
ShPT2	0.680	0.945	0.683	ID							
BisJ	0.229	0.266	0.226	0.269	ID						
PesD	0.228	0.269	0.233	0.263	0.651	ID					
UbiA	0.289	0.287	0.295	0.291	0.219	0.237	ID				
XimB	0.301	0.315	0.301	0.327	0.252	0.254	0.284	ID			
LePGT1	0.326	0.322	0.326	0.325	0.278	0.257	0.299	0.387	ID		
BvPT	0.061	0.056	0.059	0.058	0.072	0.066	0.043	0.070	0.062	ID	
BypB	0.069	0.070	0.067	0.070	0.081	0.077	0.065	0.089	0.071	0.655	ID

Species	Name<br>Accession number	Annotation	Typic reaction	Reference
Boreostereum vibrans<br>Basidiomycete	**VibP1**, ON653009<br>**VibP2**, ON653010	**membrane-bound**,<br>UbiA-type<br>prenyltransferase	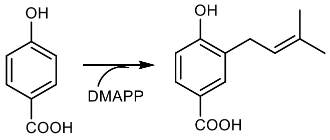 	This study
Biscogniauxia sp.<br>ascomycete	**BisJ**<br>QJQ82464			[7]
Pestalotiopsis humus<br>ascomycete	**PesD**<br>QJQ82469			
Escherichia coli<br>bacteria	**UbiA**<br>NP_418464	**membrane-bound**,<br>4-hydoxybenzoate<br>polyprenyltransferase	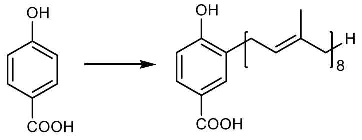 	[8, 9]
Streptomyces xiamenensis<br>bacteria	**XimB**<br>AGY49248	**membrane-bound**,<br>UbiA-type<br>prenyltransferase	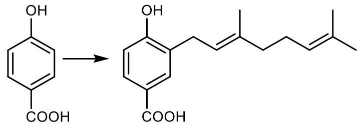 	[10]
Lithospermum erythrorhizon<br>Plant	**LePGT1**<br>BAB84122	**membrane-bound**,<br>UbiA-type<br>prenyltransferase		[11]
Boreostereum vibrans<br>Basidiomycete	**BvPT**	**soluble**,<br>aromatic<br>prenyltransferase<br>(ABBA-family)	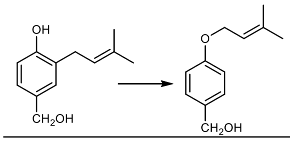 	[12]
Stereaceae family<br>(BY1)<br>basidiomycete	**BypB**<br>APH07541		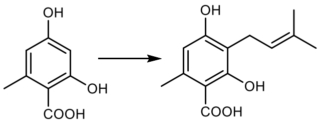 	[13]

Supplementary Fig. 51. Sequence analyses and reactions of relevant prenyltransferases.

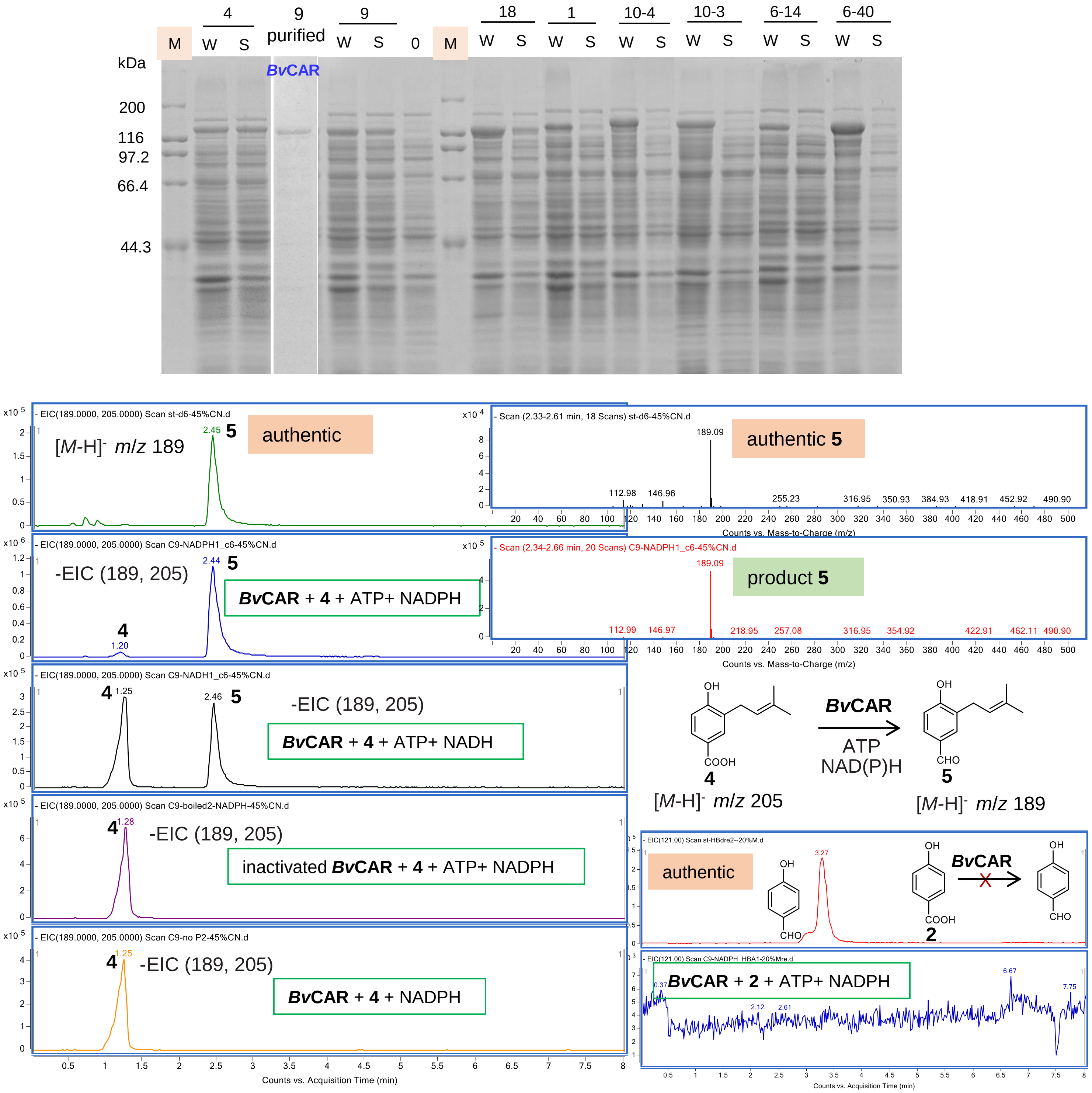

Supplementary Fig. 52. Identification of the carboxylic acid reductase (CAR) converting 4 **to** 5. Candidates were expressed in pET28a(+) / *E. coli* BL21(DE3) and induced with 0.1 mM IPTG except for the candidate 9[#] via 0.01 mM IPTG at 16 ℃ for 24 h. The SDS-PAGE experiments were repeated twice independently with similar results and the original photographs were supplied at the end of this file. M, protein size marker; 0, empty vector as blank; w, whole proteins; s, soluble proteins. Out of eight candidates, only the 9[#] protein showed the CAR activity in the in vitro enzyme assays and thereby named as ***Bv*CAR**.

The chromatographic separation for **5** was the same as described in Supplementary Fig. 31. The LC-MS extracted ion traces are corresponding to the [*M*–H]⁻ at *m*/*z* 205 for **4**, the [*M*–H]⁻ at *m*/*z* 189 for **5**. The chromatographic separation for 4-hydroxybenzaldehyde was performed with elution of 20% B over 4.9 min and 100% B over the next 3.1 min where A was H_2O and B was methanol. The mass spectrometer was run in negative ionization mode.

Seq->	BvCAR	PcCAR4	TvCAR	NcCAR	SbCAR	UttJ	NiCAR
BvCAR	ID	0.449	0.379	0.227	0.233	0.239	0.136
PcCAR4		ID	0.363	0.211	0.221	0.240	0.136
TvCAR			ID	0.236	0.212	0.231	0.137
NcCAR				ID	0.192	0.205	0.130
SbCAR					ID	0.339	0.134
UttJ						ID	0.143
NiCAR							ID

Species	Annotation<br>Accession number	Typic reaction	Reference
Boreostereum vibrans<br>Basidiomycete	***Bv*CAR**<br>ON653011	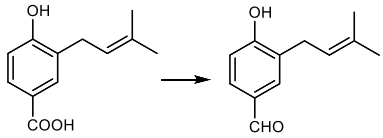	This study
Pycnoporus cinnabarinus<br>Basidiomycete	***Pc*CAR4**<br>carboxylic acid reductase<br>CDO75719	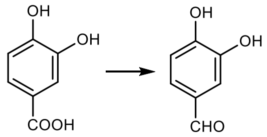	[14]
Trametes versicolor<br>Basidiomycete	***Tv*CAR**<br>carboxylate reductase<br>XP_008043822	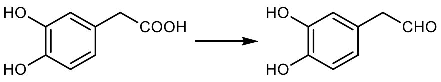	[15]
Neurospora crassa<br>Ascomycete	***Nc*CAR**<br>carboxylate reductase<br>XP_955820	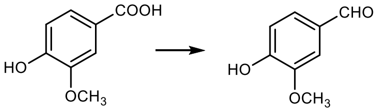	[16]
Stachybotrys bisbyi<br>Ascomycete	***Sb*CAR**<br>carboxylate reductase<br>BAV19380	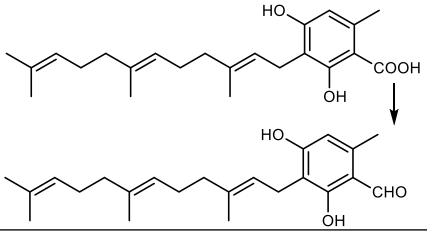	[17]
Aspergillus ustus<br>Ascomycete	**UttJ**<br>aryl acid reductase<br>KIA75587	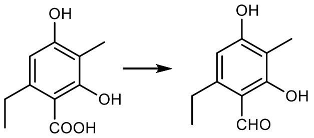	[18]
Nocardia iowensis<br>Bacteria	***Ni*CAR**<br>carboxylic acid reductase<br>Q6RKB1	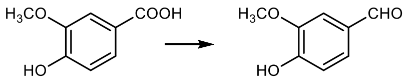	[19]

Supplementary Fig. 53. Sequence analyses and reactions of relevant CARs.

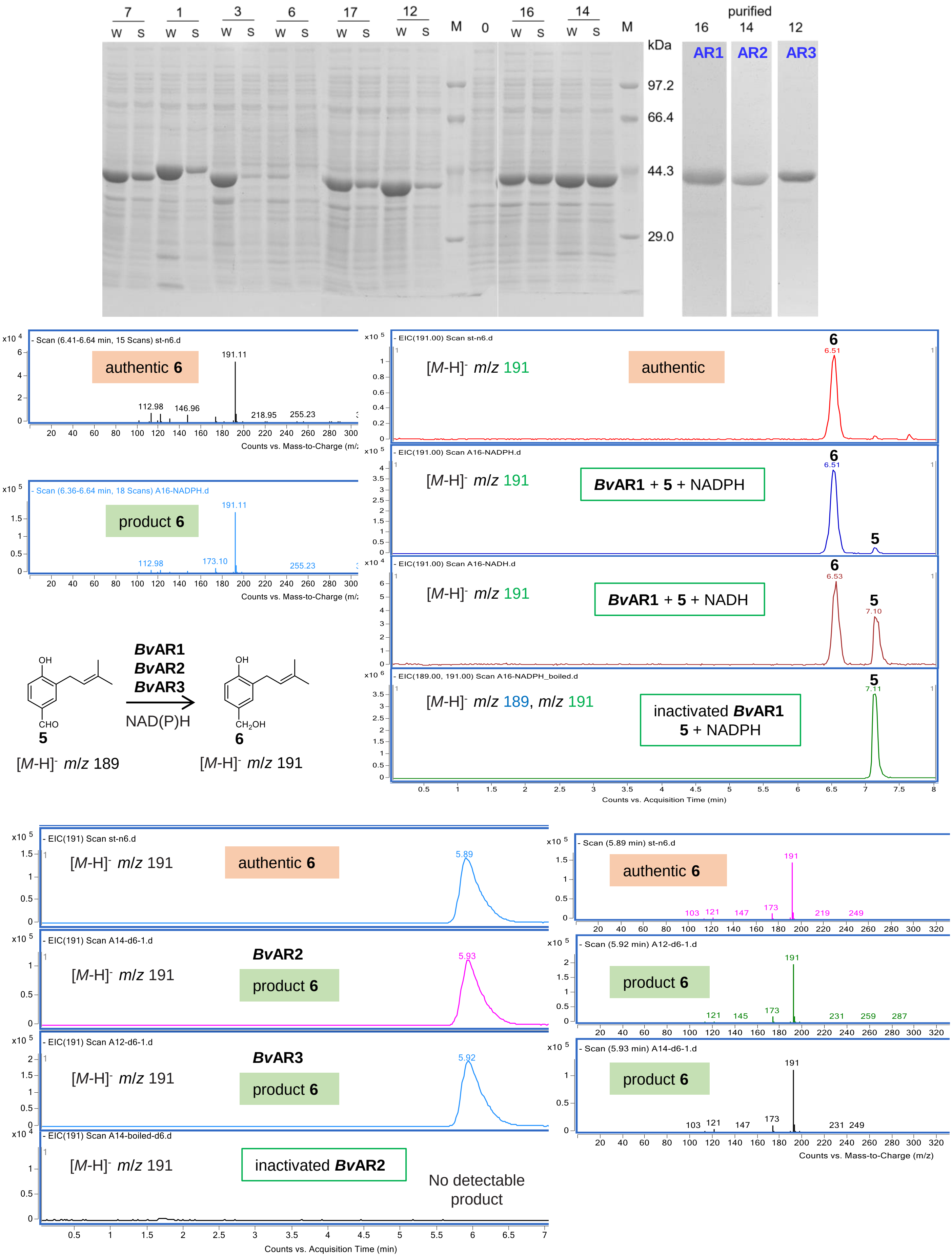

Supplementary Fig. 54. Identification of aldehyde reductases (AR) converting 5 **to** 6**.** Candidates were expressed in pET28a(+) / *E. coli* BL21(DE3) and induced with 0.1 mM IPTG at 16 °C for 24 h. The SDS-PAGE experiments were repeated twice independently with similar results and the original photographs were supplied at the end of this file. M, protein size marker; 0, empty vector as blank; w, whole proteins; s, soluble proteins. Out of eight candidates, three proteins showed the AR activity and named as ***Bv*AR1** (for 16#), ***Bv*AR2** (for 14#), and ***Bv*AR3** (for 12#), respectively. The chromatographic separation was the same as described in Supplementary Fig. 4.

Seq->	BvAR1	BvAR2	BvAR3	PcAAD	TcAAD	ADH6	YahK	ApADH
BvAR1	ID	0.219	0.682	0.255	0.627	0.079	0.096	0.054
BvAR2		ID	0.232	0.201	0.215	0.051	0.052	0.060
BvAR3			ID	0.253	0.642	0.076	0.088	0.070
PcAAD				ID	0.215	0.058	0.054	0.061
TcAAD					ID	0.086	0.107	0.072
ADH6						ID	0.319	0.206
YahK							ID	0.273
ApADH								ID

aldehyde reductases (**AR**) / aryl-alcohol dehydrogenases (**AAD**) / alcohol dehydrogenase (**ADH**)

Species	Annotation<br>Accession number	Typic reaction	Reference
Boreostereum vibrans<br>Basidiomycete	***Bv*AR**<br>ON653012<br>ON653013<br>ON653014	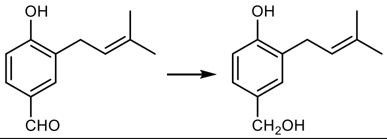	This study
Phanerodontia chrysosporium<br>Basidiomycete	***Pc*AAD**<br>aryl-alcohol dehydrogenase [NADP(+)]<br>Q01752	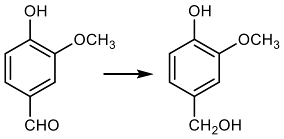	[20]
Taiwanofungus camphoratus<br>Basidiomycete	***Tc*AAD**<br>aryl-alcohol dehydrogenase<br>ADV77220	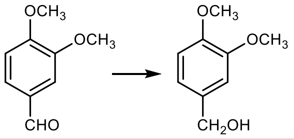	[21]
Saccharomyces cerevisiae<br>Ascomycete	**ADH6**<br>NADP-dependent alcohol dehydrogenase 6<br>Q04894	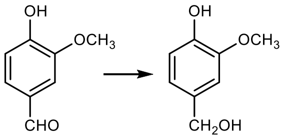	[22]
Escherichia coli K-12<br>Bacteria	**YahK**<br>aldehyde reductase<br>P75691 (PDB: 1UUF)	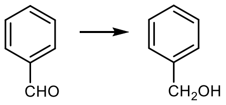	[23]
Aeropyrum pernix<br>Bacteria	***Ap*ADH**<br>alcohol dehydrogenase<br>WP_010866880	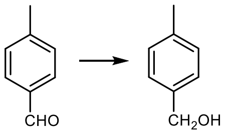	[24]

Supplementary Fig. 55. Sequence analyses and reactions of relevant aldehyde reductases.

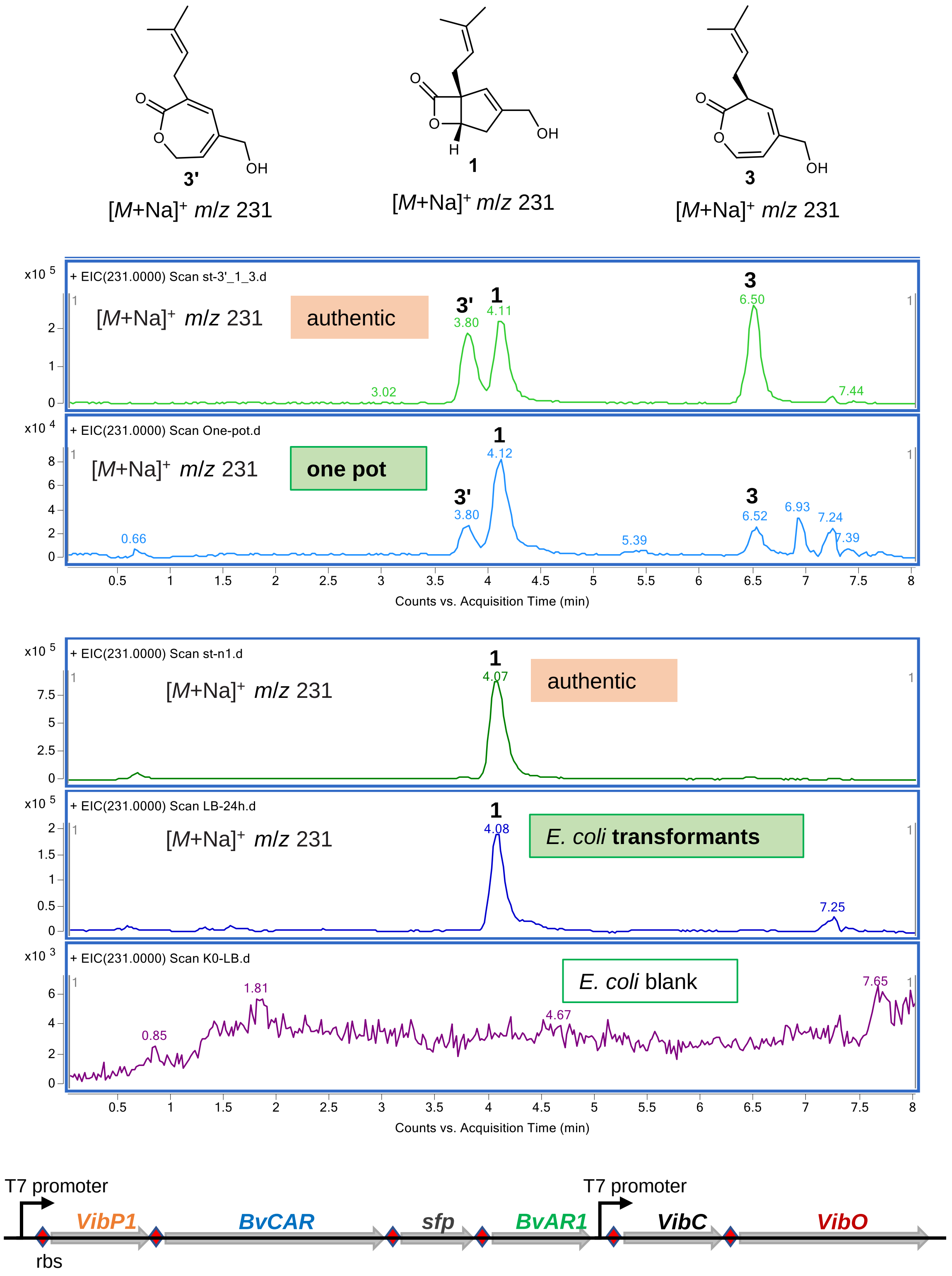

Supplementary Fig. 56. The original LC-MS data for in vitro **(one-pot)** and in vivo **(*E. coli*)** reconstruction of the vibralactone pathway, and schematic illustration of the constructed plasmid (rbs, ribosome-binding site). The chromatographic separation was the same as described in Supplementary Fig. 4.

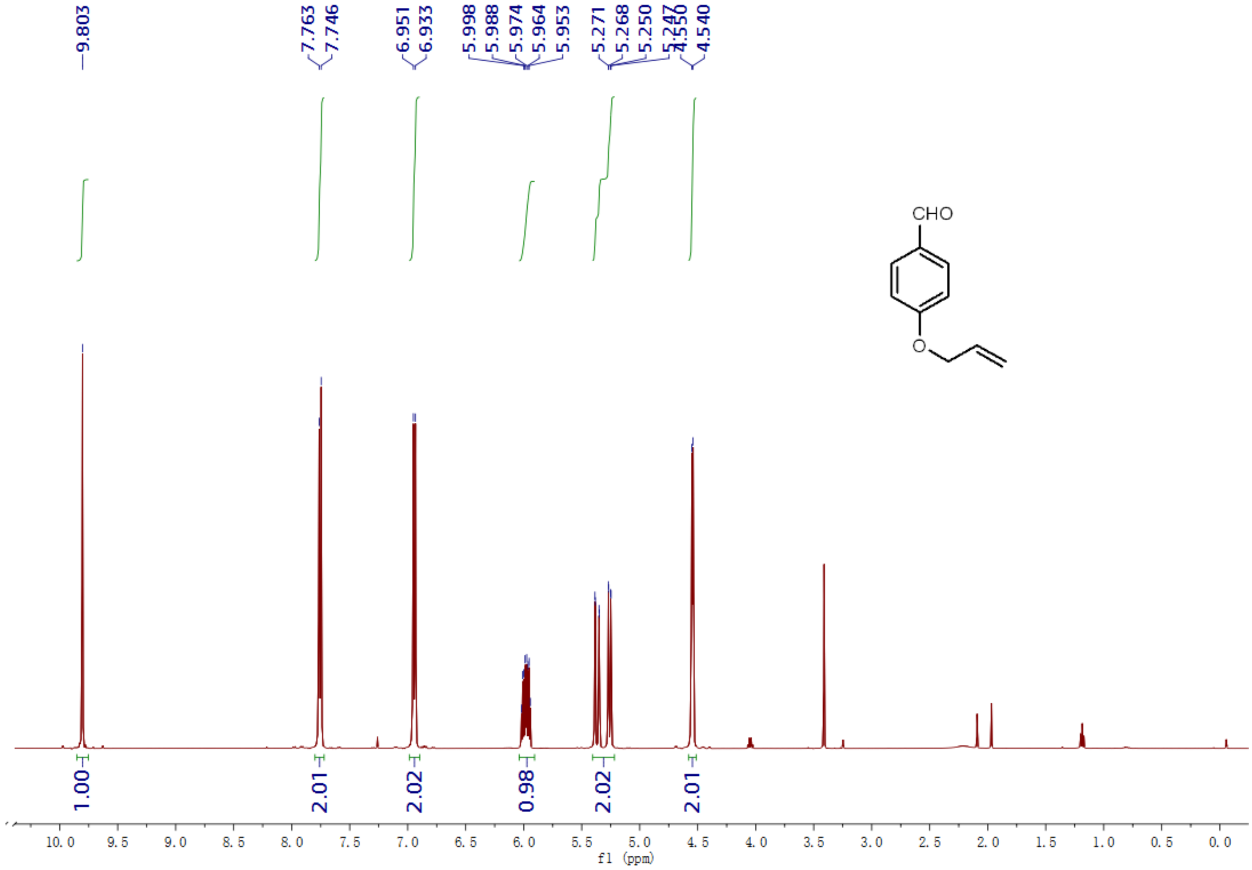

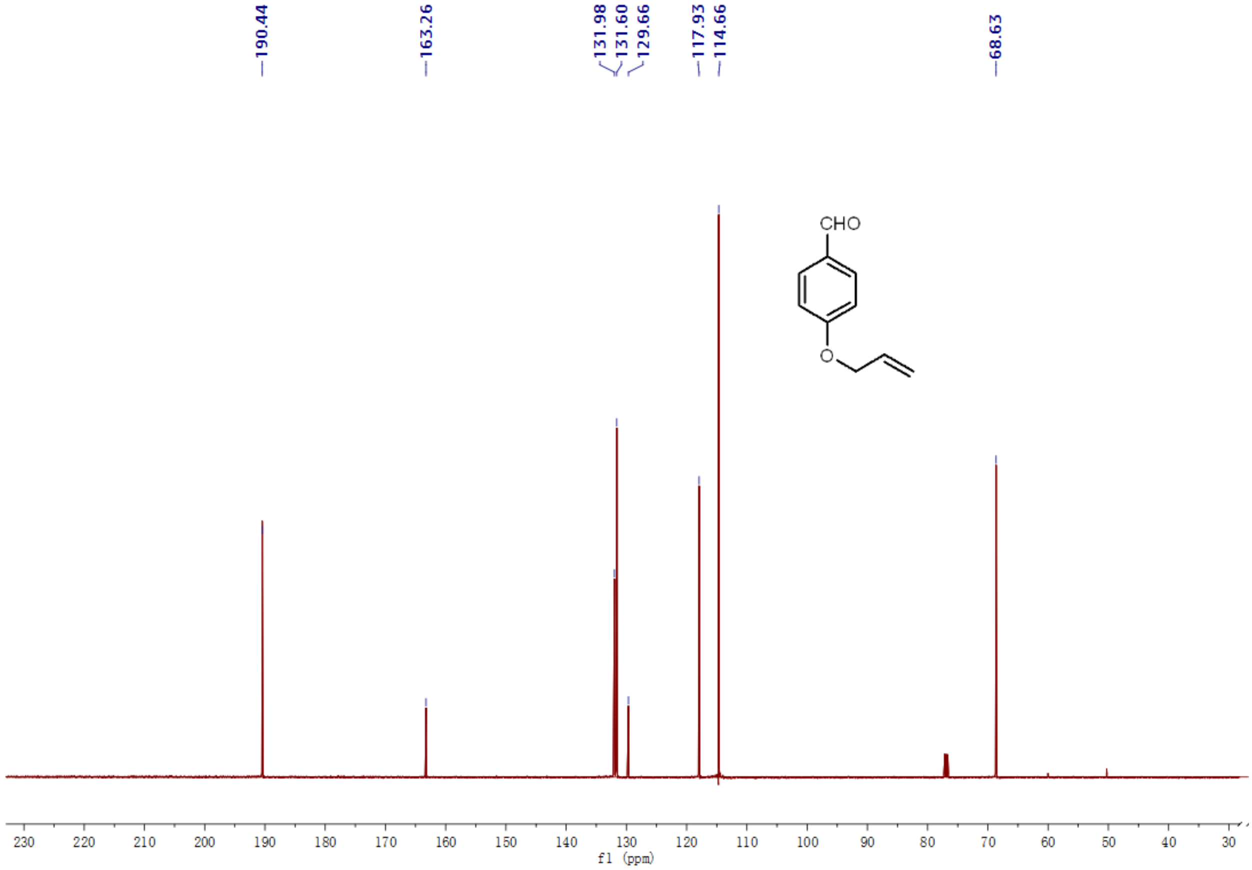

Supplementary Fig. 57. ^{1}H-NMR spectrum (500 MHz, $CDCl_3$) and ^{13}C-NMR spectrum (126 MHz, $CDCl_3$) of **5a′**.

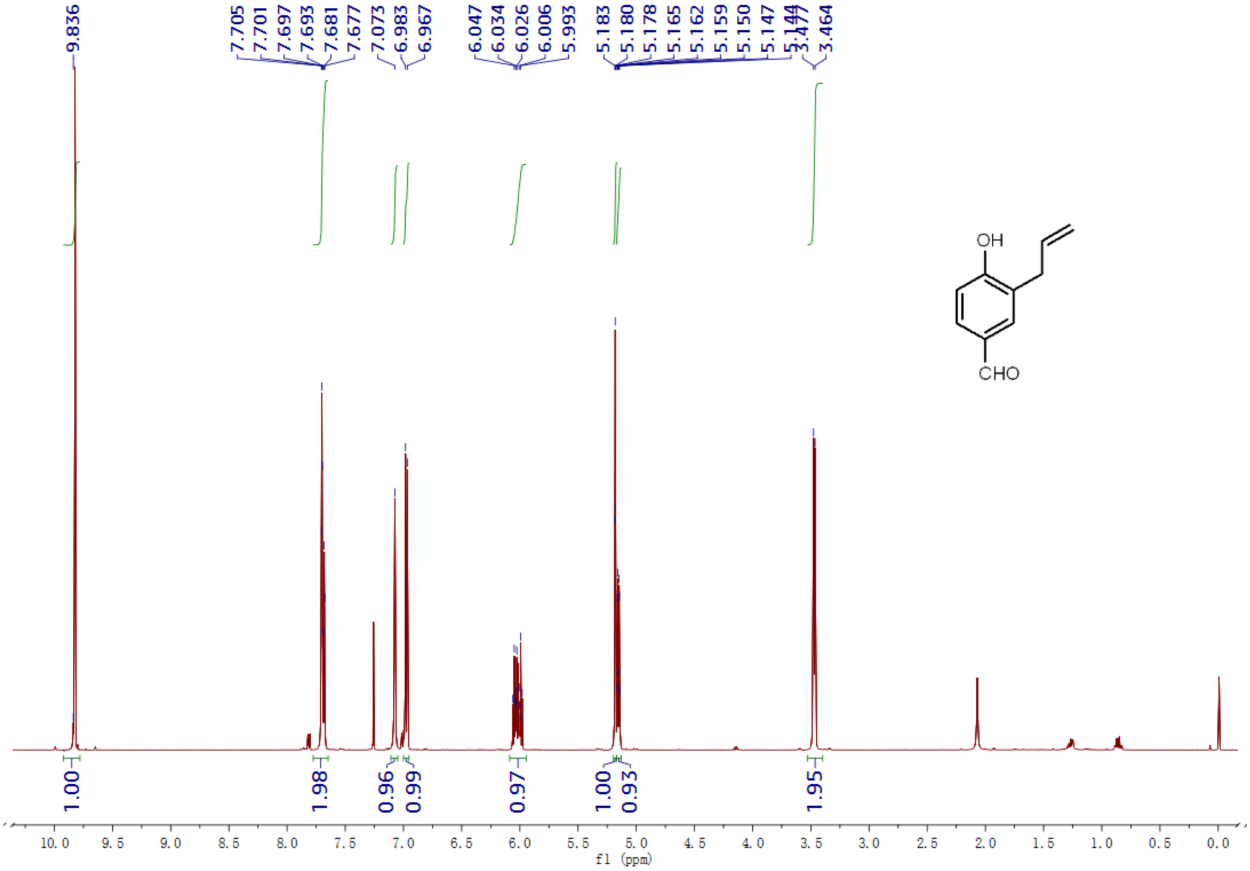

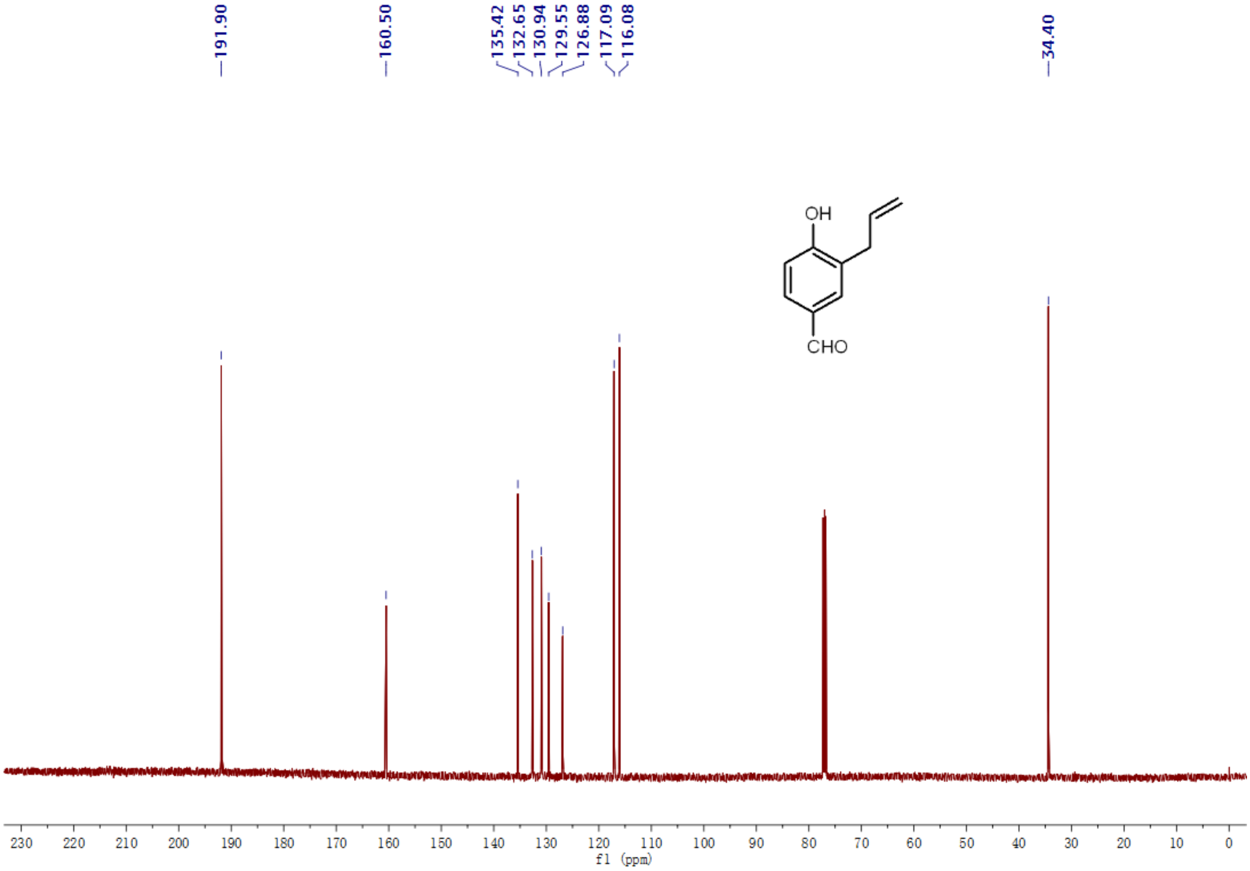

Supplementary Fig. 58. ^{1}H-NMR spectrum (500 MHz, $CDCl_3$) and ^{13}C-NMR spectrum (126 MHz, $CDCl_3$) of **5a**.

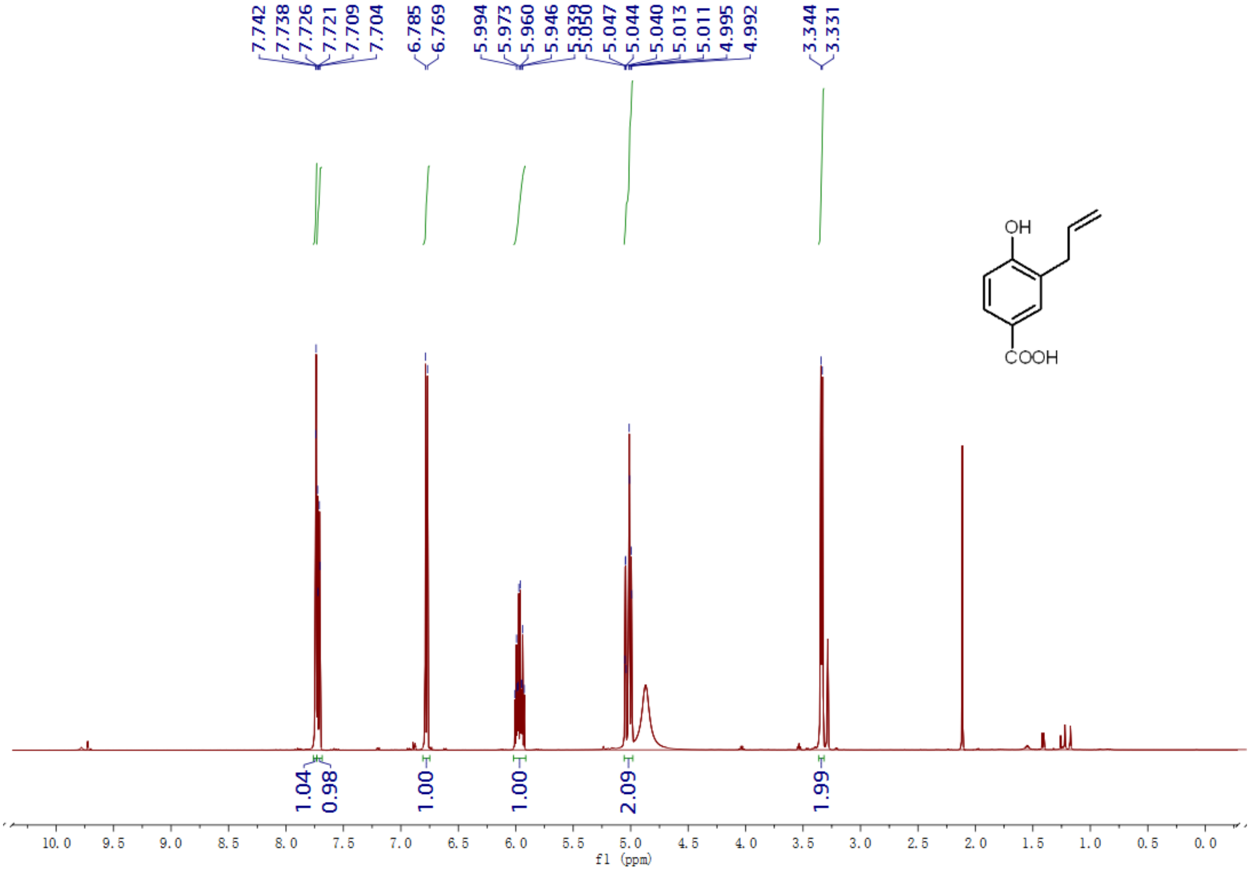

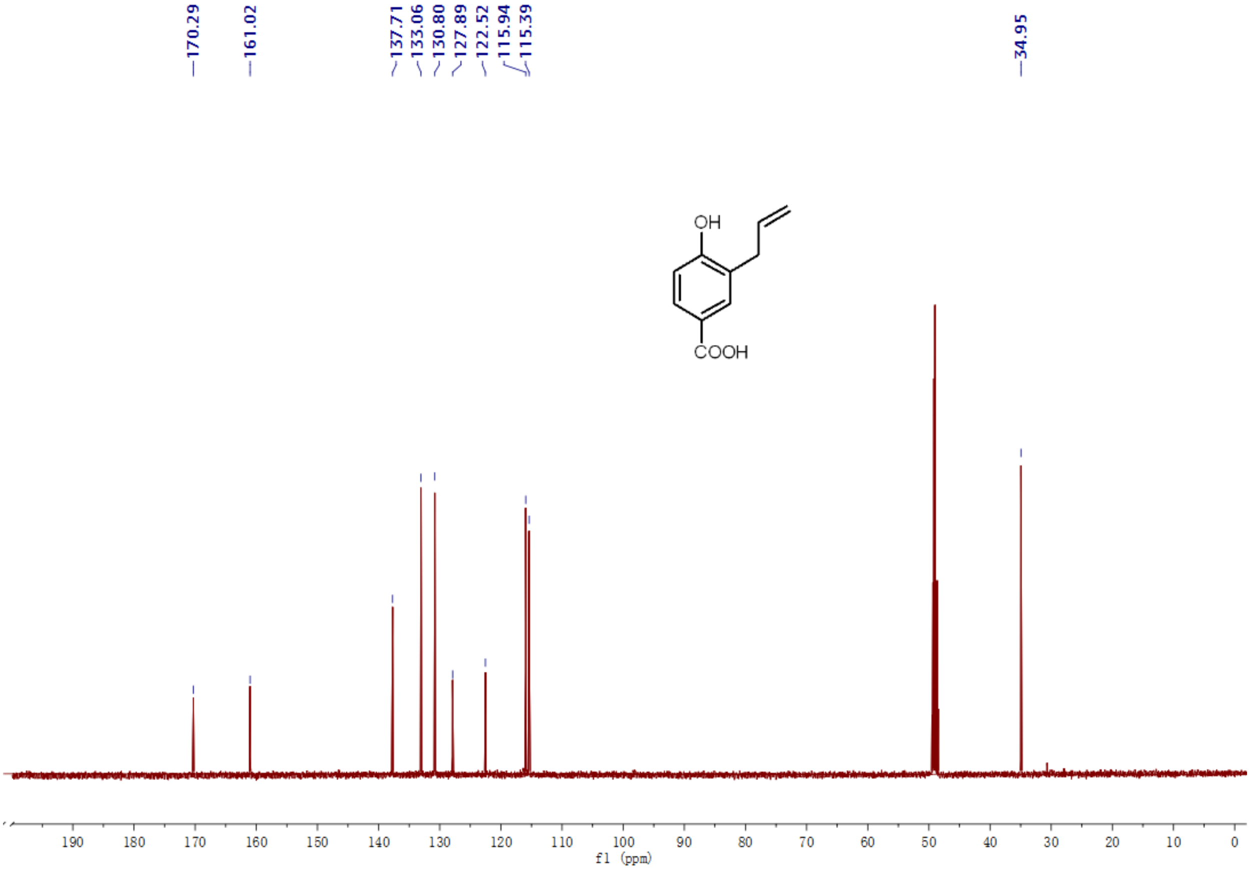

Supplementary Fig. 59. ^{1}H-NMR spectrum (500 MHz, MeOD) and ^{13}C-NMR spectrum (126 MHz, MeOD) of **4a**.

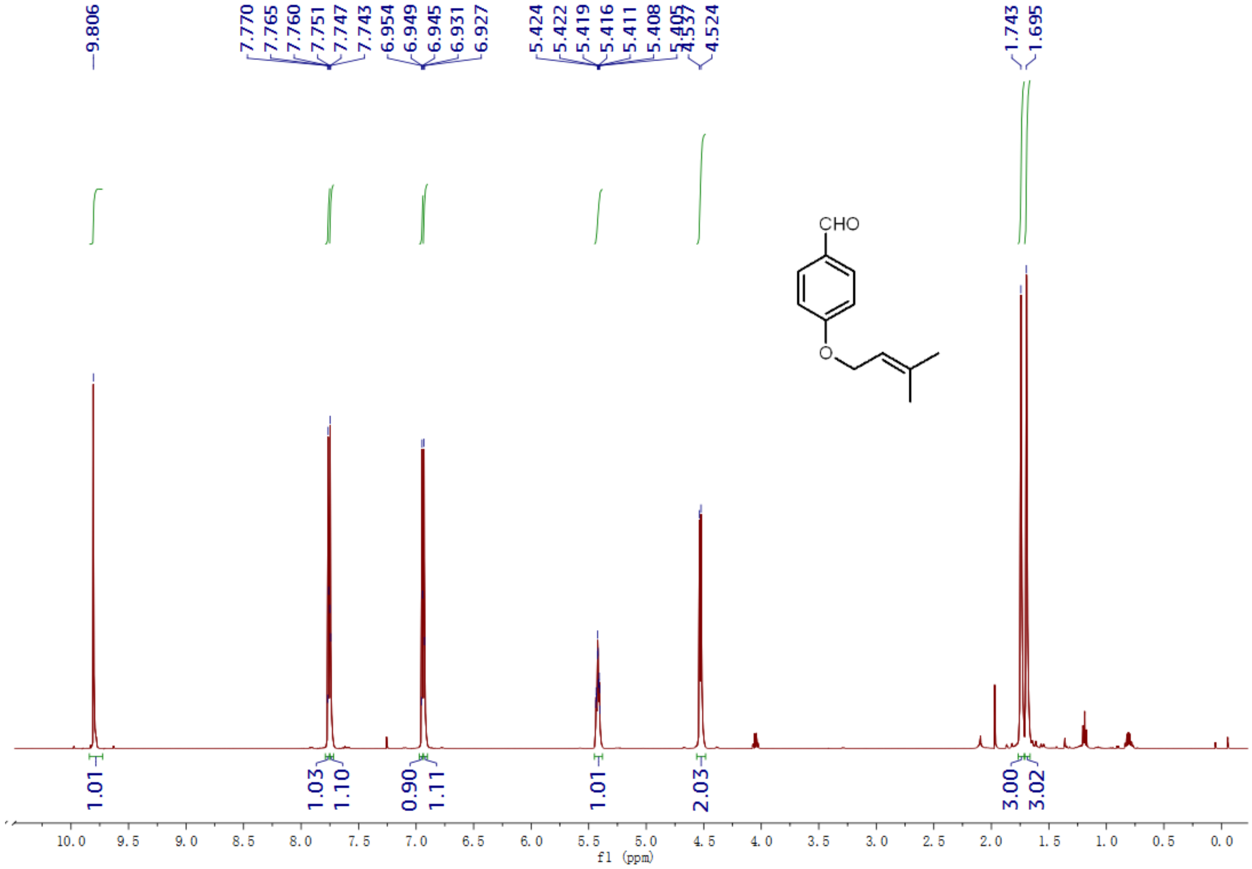

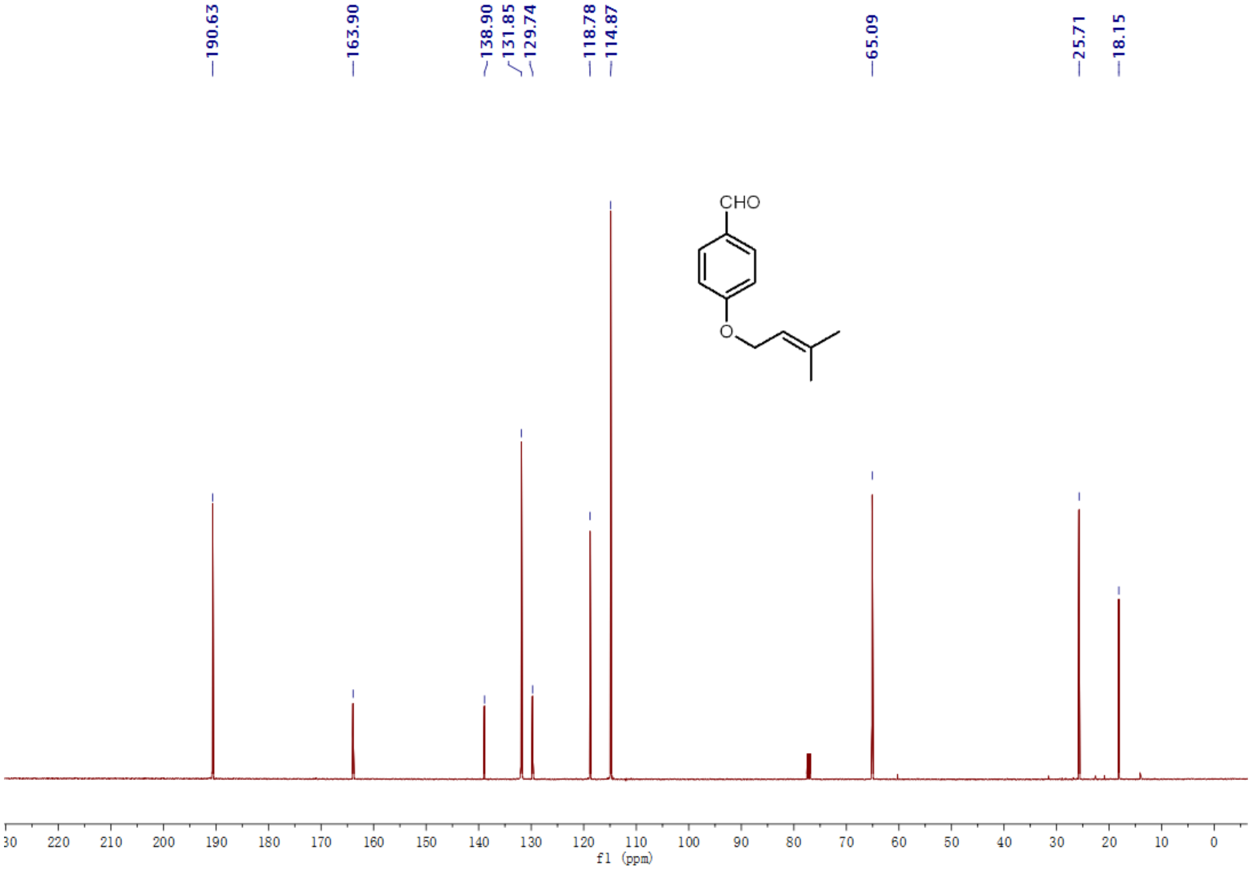

Supplementary Fig. 60. ^{1}H-NMR spectrum (500 MHz, $CDCl_3$) and ^{13}C-NMR spectrum (126 MHz, $CDCl_3$) of **5′**.

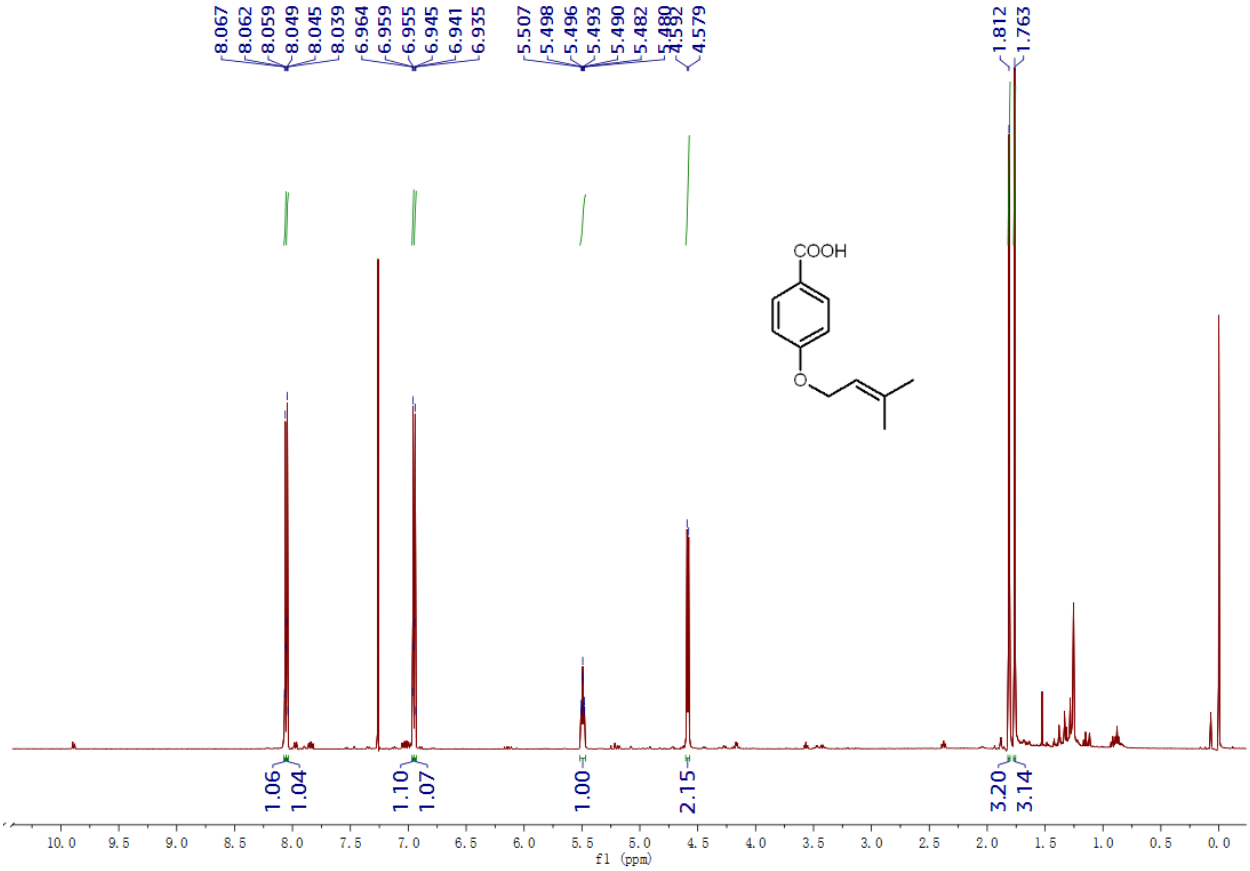

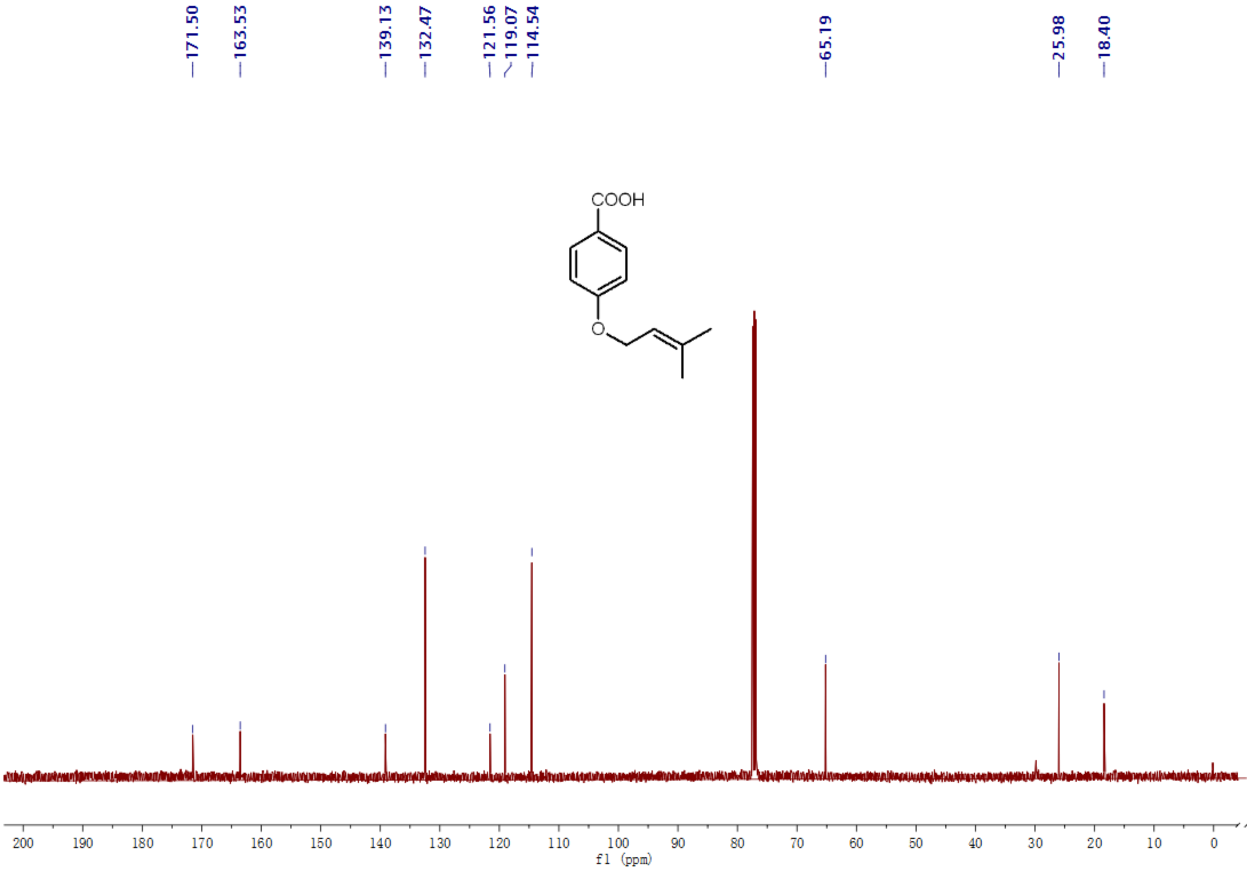

Supplementary Fig. 61. ^{1}H-NMR spectrum (500 MHz, $CDCl_3$) and ^{13}C-NMR spectrum (126 MHz, $CDCl_3$) of **4′**.

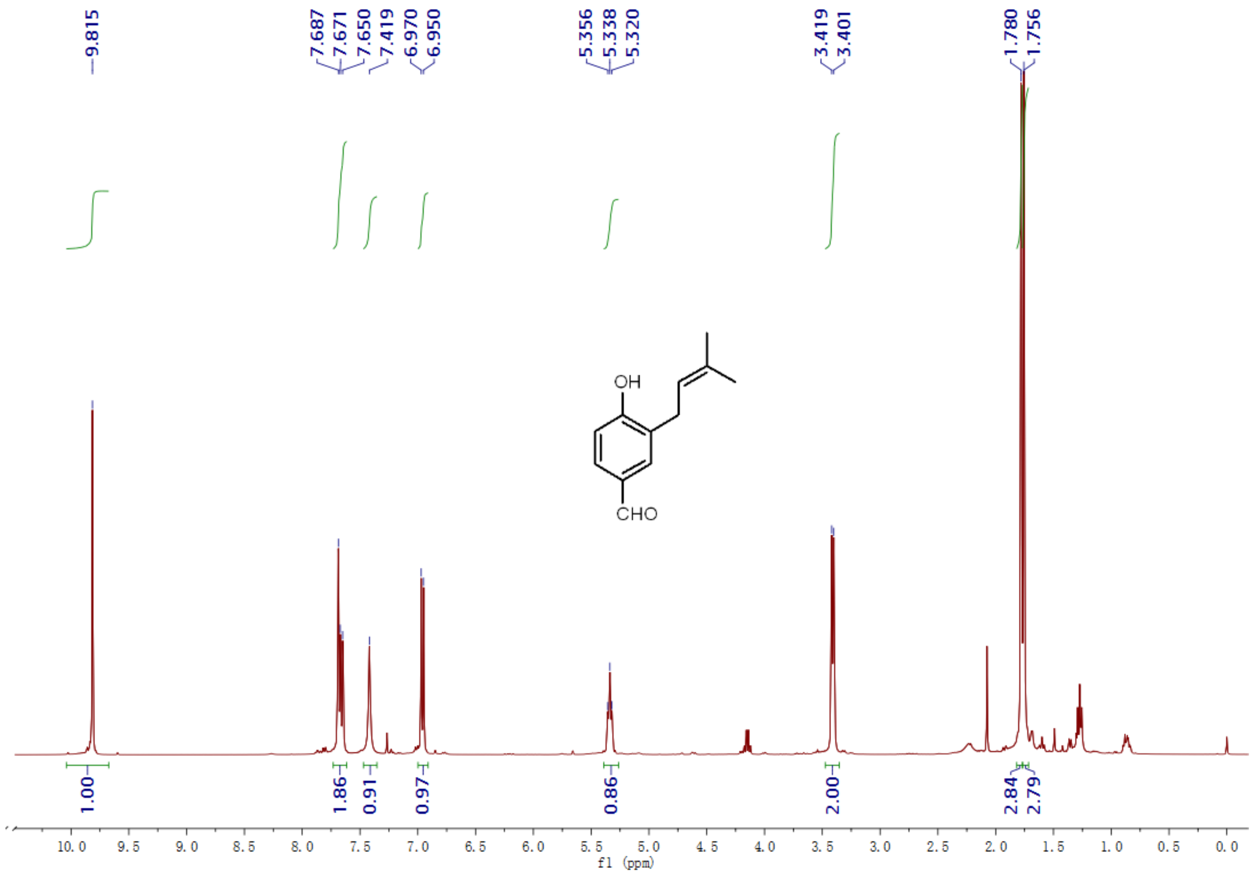

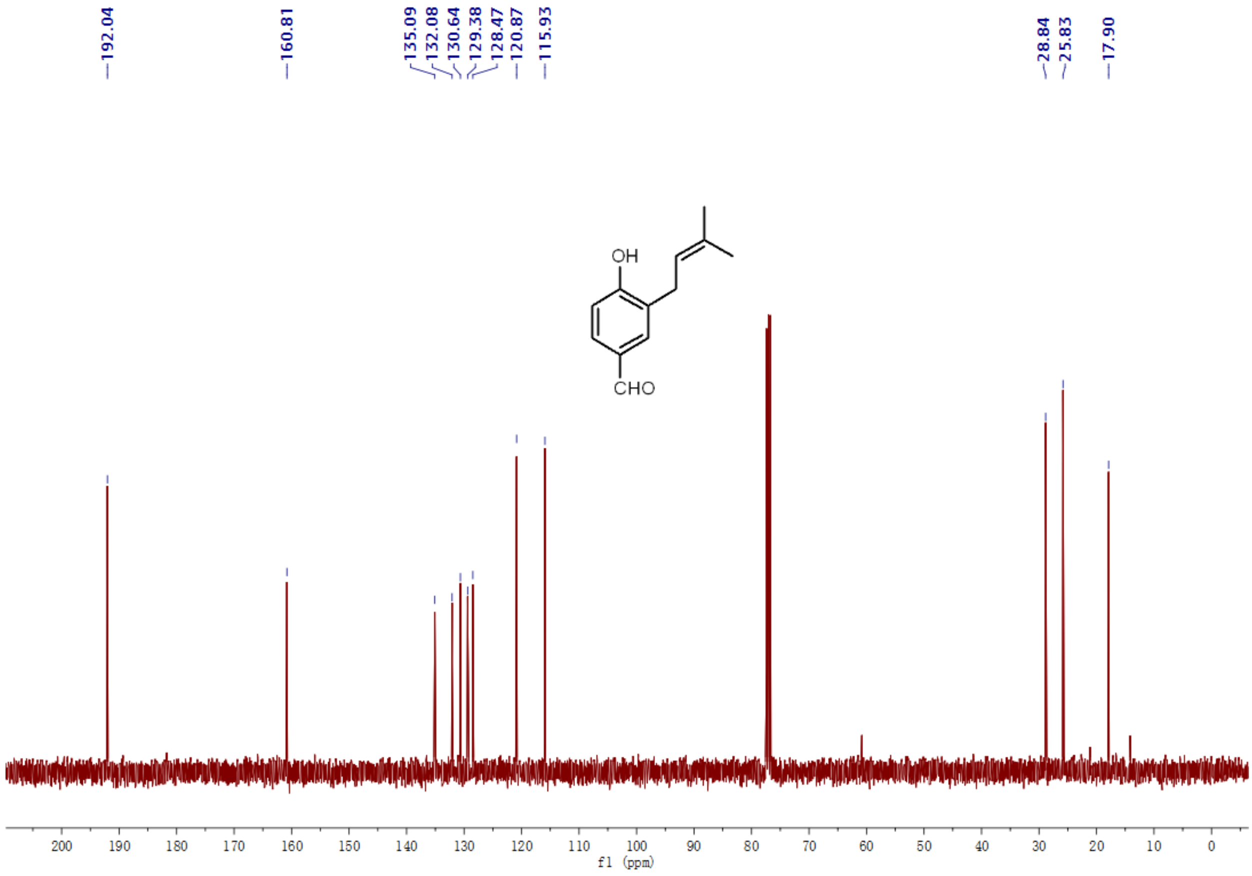

Supplementary Fig. 62. ^{1}H-NMR spectrum (400 MHz, $CDCl_3$) and ^{13}C-NMR spectrum (101 MHz, $CDCl_3$) of **5**.

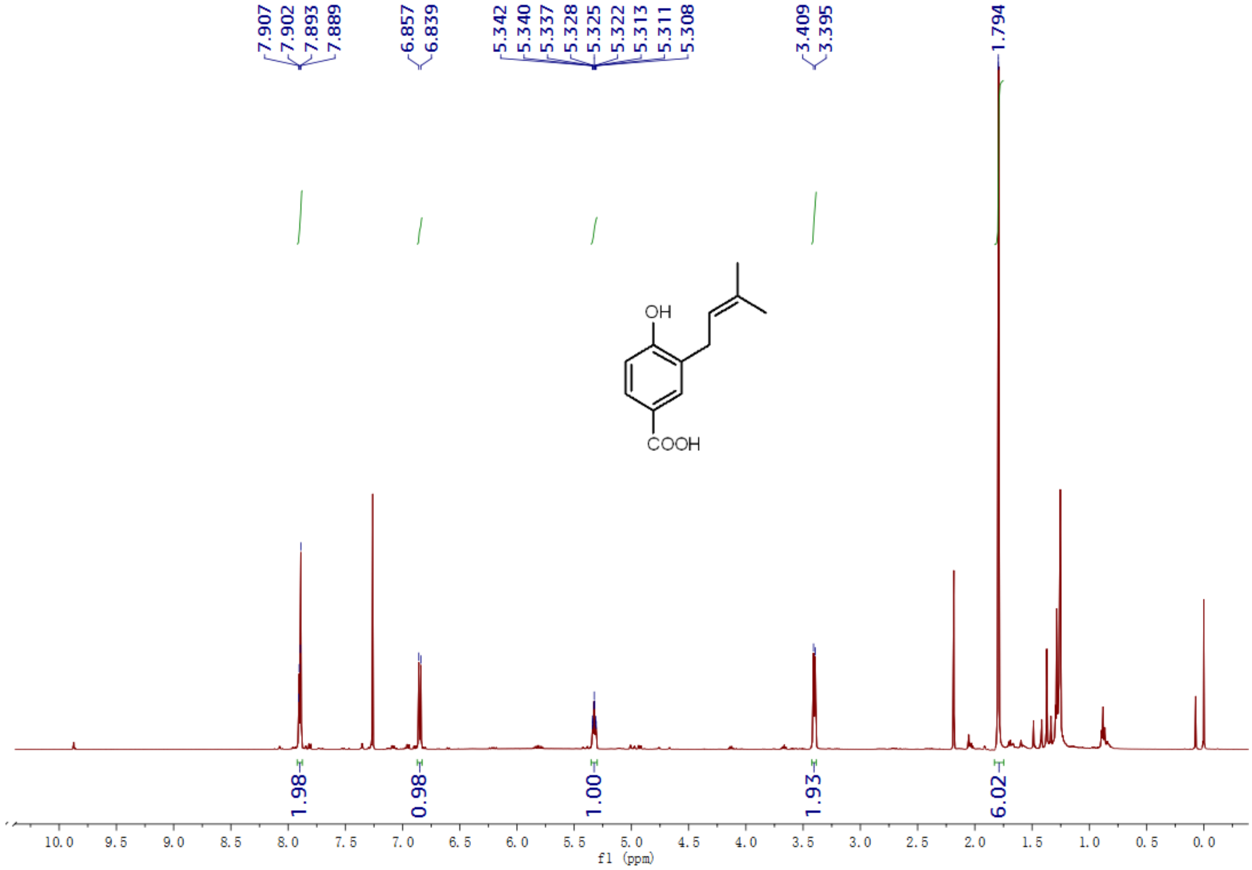

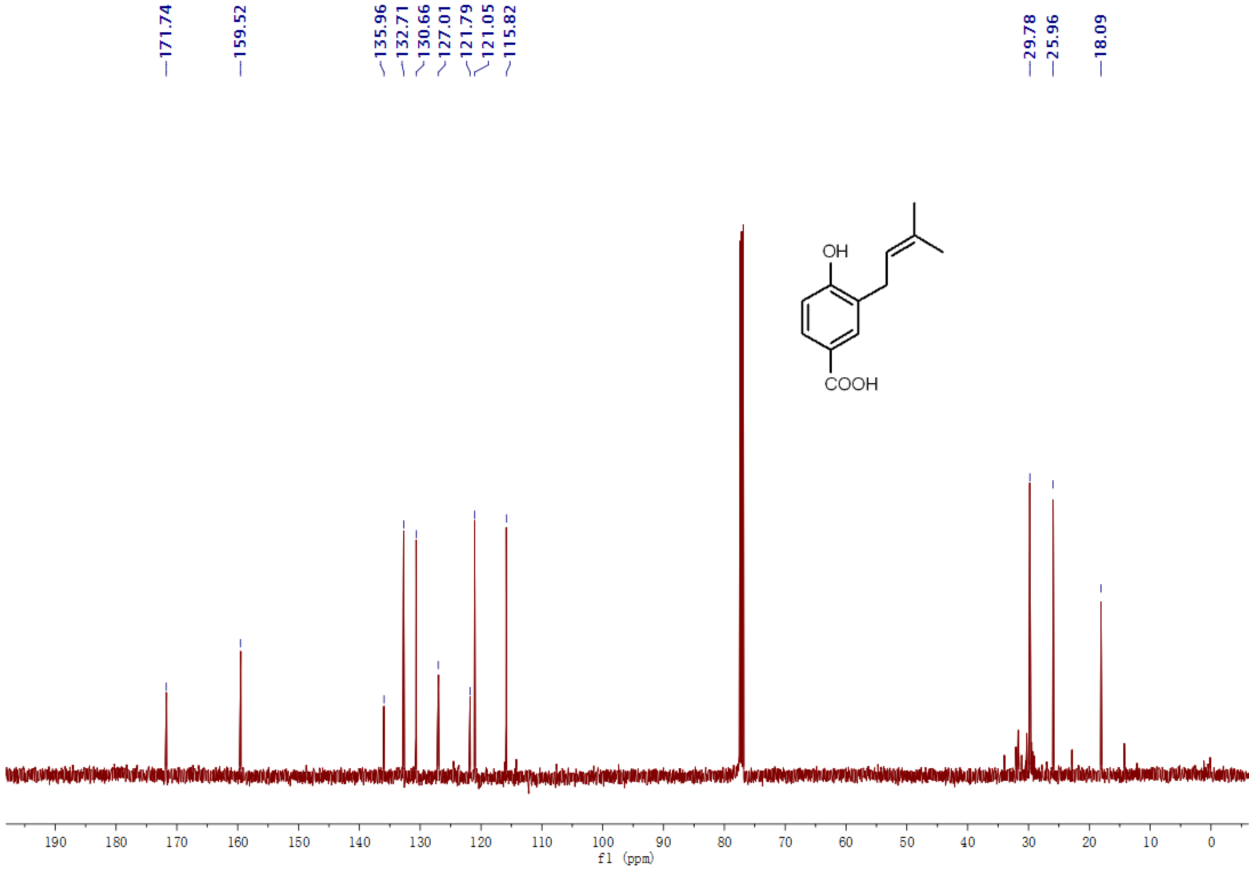

Supplementary Fig. 63. ^{1}H-NMR spectrum (500 MHz, $CDCl_3$) and ^{13}C-NMR spectrum (126 MHz, $CDCl_3$) of **4**.

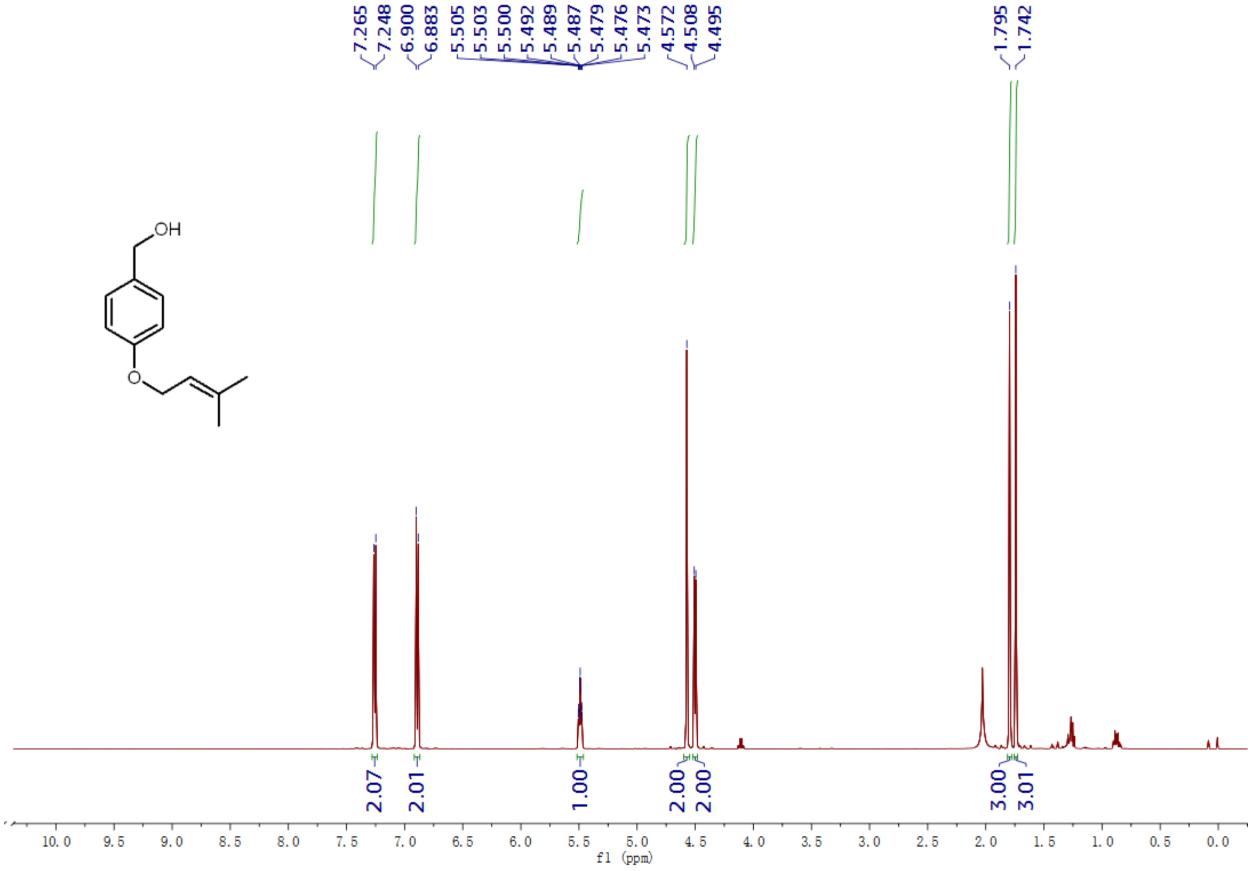

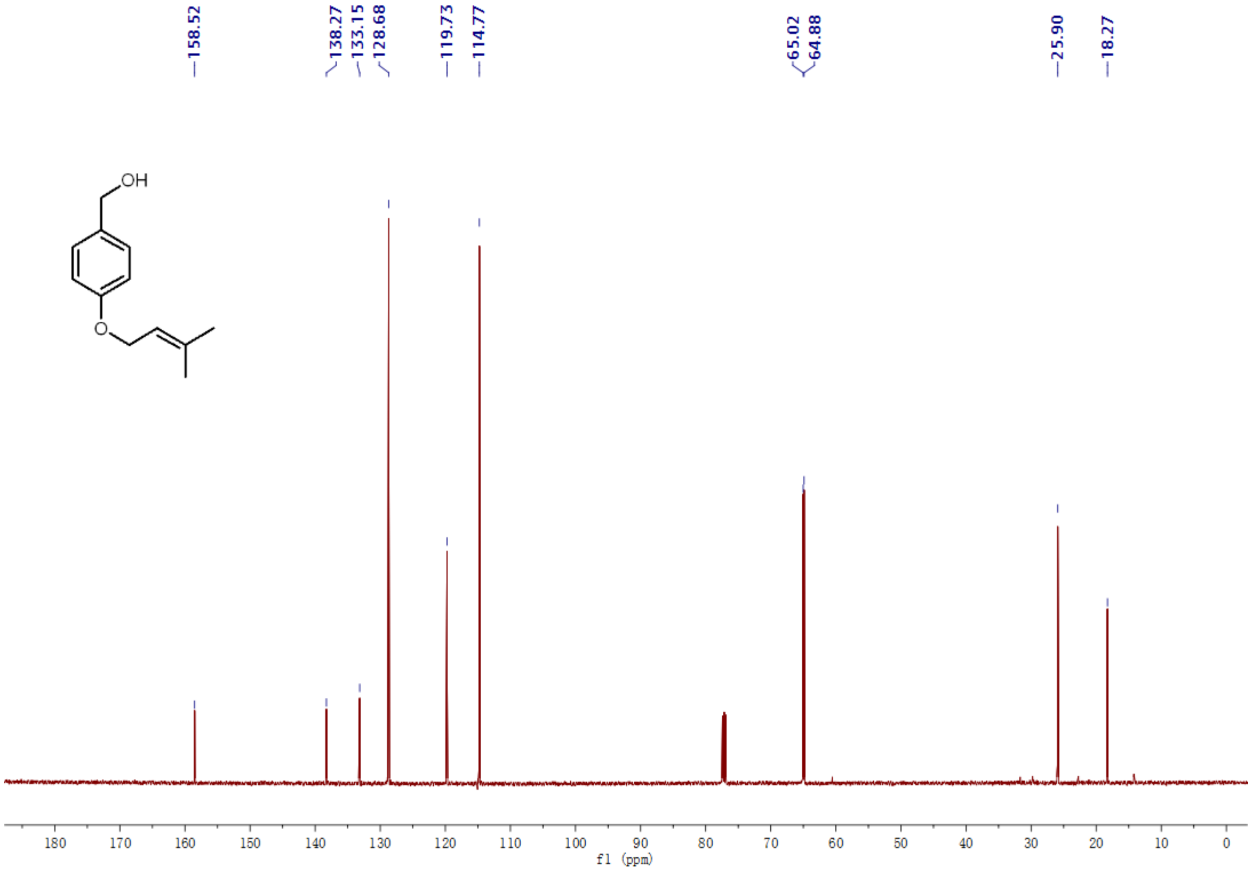

Supplementary Fig. 64. ^{1}H-NMR spectrum (500 MHz, $CDCl_3$) and ^{13}C-NMR spectrum (126 MHz, $CDCl_3$) of **6′**.

Supplementary Tables

Supplementary Table 1. Statistics of X-ray crystallographic data collection and model refinement

**Data collection**	
Space group	*P*1
Unit cell parameters	
a, *b*, *c* (Å)	77.08, 92.50, 104.64
α, β, γ (°)	89.87, 74.92, 75.75
Resolution range (Å)	71.98 - 2.43 (2.47 - 2.43)
No. of total reflections	327479
No. of unique reflections	94891
I/σ	6.0 (2.2)
Completeness (%)	92.8 (95.1)
R_{merge} (%)[a]	7.4 (53.5)
CC1/2	0.998 (0.877)
**Structure refinement**	
Resolution (Å)	34.38 - 2.43
R_{cryst} /R_{free} (%)[b]	17.9(24.7)/23.0(31.2)
r.m.s.d bonds (Å)/angles (º)	0.010/1.155
No. of reflection working set /test set	94834/4737
No. of atoms	
macromolecules	19275
ligand	218
water	444
B-factor	
macromolecules	51.24
ligand	51.31
water	45.50
Ramachandran plot	
most favored regions (%)	95.93
additionally allowed (%)	3.79
generously allowed (%)	0.28

[a] $R_{merge} = \sum|I_i - I_m|/\sum I_i$, where I_i is the intensity of the measured reflection and I_m is the mean intensity of all symmetry related reflections.

[b] $R_{cryst} = \Sigma||F_{obs}| - |F_{calc}||/\Sigma|F_{obs}|$, where F_{obs} and F_{calc} are observed and calculated structure factors.

$R_{free} = \Sigma_T||F_{obs}| - |F_{calc}||/\Sigma_T|F_{obs}|$, where T is a test data set of about 5% of the total reflections randomly chosen and set aside prior to refinement.

Numbers in parentheses represent the value for the highest resolution shell.

Supplementary Table 2. Primers for cloning VibO candidates

Primer names	Oligonucleotide sequences with adaptors underlined (5′ to 3′)
20-f	AAATGGGTCGCGGATCC**ATG**ATATCCGGGGTCACTGG
20-r	AAGCTTGTCGACGGAGCTC**TCA**AGCGACGAACGCATAGC
21-f	AAATGGGTCGCGGATCC**ATG**TCGACTATTACTCATACGCC
21-r	AAGCTTGTCGACGGAGCTC**CTA**CTCATCCCCCAACTCCTC
22-f	AAATGGGTCGCGGATCC**ATG**TCTCCCTCCAGCAGTC
22-r	AAGCTTGTCGACGGAGCTC**TCA**GTCCATCAAAAAGTCTGC
23-f	AAATGGGTCGCGGATCC**ATG**ACAACTTCATCTGCTGT
23-r	AAGCTTGTCGACGGAGCTC**TCA**AGCATCCTTCAAGAAGC
24-f	AAATGGGTCGCGGATCC**ATG**AGCGATACTCACTCGCATG
24-r	AAGCTTGTCGACGGAGCTC**TCA**ATCAACCAGAACCACGC
25-f	AAATGGGTCGCGGATCC**ATG**AGCGACACTCACTCGCA
25-r	AAGCTTGTCGACGGAGCTC**TTA**ATCCACCAGAATCACACTA
26-f	AAATGGGTCGCGGATCC**ATG**TCTCCTCTTCCAAAGACC
26-r	AAGCTTGTCGACGGAGCTC**TCA**AGGGAACACGATGACGT
27-f	AAATGGGTCGCGGATCC**ATG**ACCTCTGCATCCTCCC
27-r	AAGCTTGTCGACGGAGCTC**TTA**CCCGAATCCATAGGCATG
28-f	AAATGGGTCGCGGATCC**ATG**CTGGTTCGTATCATGAAG
28-r	AAGCTTGTCGACGGAGCTC**TTA**GGAGAACGCTTGGATCC
29-f	AAATGGGTCGCGGATCC**ATG**GCGGCCCTCAACATCAA
29-r	AAGCTTGTCGACGGAGCTC**CTA**CTCTGGGTCCCTCTTCC
30-f	AAATGGGTCGCGGATCC**ATG**GCGGCGCCCAACATTAA
30-r	AAGCTTGTCGACGGAGCTC**CTA**CTCTGGGTCCCTCTTCC
31-f	AAATGGGTCGCGGATCC**ATG**GCTGCCATCGCAGAAATC
31-r	AAGCTTGTCGACGGAGCTC**TTA**CAGAGGCCAATCAAGCT
32-f	AAATGGGTCGCGGATCC**ATG**ATTCCACGTCTGCACG
32-r	AAGCTTGTCGACGGAGCTC**CTA**CATACTATTCGAGTCTTTGA
33-f	AAATGGGTCGCGGATCC**ATG**TCTGCTGTTGAAAATTTCG
33-r	AAGCTTGTCGACGGAGCTC**TTA**AGAAGAACCCTCAGTCG
34-f	AAATGGGTCGCGGATCC**ATG**GCAACGGCCCTTAATTC
34-r	AAGCTTGTCGACGGAGCTC**TCA**GTCGAAGGCCTTACTCG
35-f (for VibO)	AAATGGGTCGCGGATCC**ATG**GCTGCTGTTGAAAACTTCG
35-r (for VibO)	AAGCTTGTCGACGGAGCTC**TTA**AGAAGAACCCTCAGTCGAC
36-f	AAATGGGTCGCGGATCC**ATG**GCTTCACCTGCACCCA
36-r	AAGCTTGTCGACGGAGCTC**TTA**TTCCTTATTTCCTTTCCAAAGC
37-f	AAATGGGTCGCGGATCC**ATG**GCGGCCCCCAATGGCCA
37-r	AAGCTTGTCGACGGAGCTC**CTA**GTCAACGACCTCCTCCTCAA
41-f	AAATGGGTCGCGGATCC**ATG**TCCAAGACTCGCGTAGC
41-r	AAGCTTGTCGACGGAGCTC**CTA**CGTACCAGCACTTTGCG
*Sh*35-f	AAATGGGTCGCGGATCC**ATG**GCCACCTTTGAGACTTTC
*Sh*35-r	AAGCTTGTCGACGGAGCTC**TTA**AGAAGAACCCTCAGTCGAC
*Sh*33-f	AAATGGGTCGCGGATCC**ATG**GCTGCCGTTCAGAACTTC
*Sh*33-r	AAGCTTGTCGACGGAGCTC**CTA**AACAGTACCCTGGGTTGAC

Supplementary Table 3. Primers for cloning VibO mutants

Primer names	Sequences with the codon for site-specific mutation marked in red (5′ to 3′)
R97A-f	CCCAGTTGGCGCTGCCGATGGACTG
R97A-r	CAGGTAGCCGTCGCGGTTGACCCTC
D99A-f	GTTGGCCGTGCCGCTGGACTGCAACCTCGTTCGATGGAGG
D99N-f	GTTGGCCGTGCCAATGGACTGCAACCTCGTTCGATGGAGG
D99N-r	CCAACGTCAGGTAACCGTGCCGGTTGACCCTCTC
F265Y-f	CACCGCTGGACTTACGGTACCGTCCTCAACTCTGAGTACGG
F265Y-r	CTCCTGCCATGGCATTCAGGTCGCCACTCCCTTCAGTC
I279F-f	GATGTCTTATTTTCCCTCGCGAGCGCAATATGGTCCGTCTCTACG
I279F-r	CGAGCGCTCCCTTTTATTCTGTAGGTGGCATGAGTC
R281A-f	CTTATTATCCCTGCCGAGCGCAATATGG
R281A-r	TATAACGCGAGCCGTCCCTATTATTCTG
R287A-f	GCAATATGGTCGCTCTCTACGTTCAGC
R287A-r	GACTTGCATCTCTCGCTGGTATAACGCG
Y289A-f	GGTCCGTCTCGCCGTTCAGCTCCGTGCTGAGCCTGG
Y289A-r	GCCTCGACTTGCCGCTCTGCCTGGTATAACGCGAGCG
Y289F-f	GGTCCGTCTCTTCGTTCAGCTCCGTGCTGAGCCTGG
Y289F-r	GCCTCGACTTGCTTCTCTGCCTGGTATAACGCGAGCG
Y289N-f	GGTCCGTCTCAACGTTCAGCTCCGTGCTGAGCCTGG
Y289N-r	GCCTCGACTTGCAACTCTGCCTGGTATAACGCGAGCG
A366L-f	GTGCCAAGGGCCTCTTCGGCATGAACACCGGTGTCATGGACG
A366L-r	CAAGTACGGCTTCTCCGGGAACCGTGACACCTGCACCG
A366Q-f	GTGCCAAGGGCCAGTTCGGCATGAACACCGGTGTCATGGACG
A366Q-r	CAAGTACGGCTTGACCGGGAACCGTGACACCTGCACCG
V459L-f	CAAGAAGTTCCTCGGTCAGGTCGGGCGGTTCCTCATCGGTC
V459L-r	GGCTGGACTGGCTCCTTGAAGAACTTCATCTTGTGCAG

Supplementary Table 4. Primers for cloning prenyltransferase candidates

Primer names	Oligonucleotide sequences with adaptors underlined (5′ to 3′)
1-f (for VibP2)	AAATGGGTCGCGGATCC**ATG**TCTTCCGCTACCACTCC
1-r (for VibP2)	AAGCTTGTCGACGGAGCTC**TCA**GAAGGGCATCTTGAAGGG
4-f	AAATGGGTCGCGGATCC**ATG**TCGTTCGCTGTGCAG
4-r	AAGCTTGTCGACGGAGCTC**TCA**TGAGAGTAATTTCATGACGGTCG
5-f	AAATGGGTCGCGGATCC**ATG**CCGACTTTAACATCTTCGG
5-r	AAGCTTGTCGACGGAGCTC**CTA**CCAAATAAGTATATGCTGACGAGC
6-f (for VibP1)	AAATGGGTCGCGGATCC**ATG**GCCGACGCACACACC
6-r (for VibP1)	AAGCTTGTCGACGGAGCTC**CTA**GAGTTTTGGAAGTGGAATAGCATCG
7-f	AAATGGGTCGCGGATCC**ATG**CTGACTCGTGCTGCATAC
7-r	AAGCTTGTCGACGGAGCTC**TCA**CGACATGACCCGTCCTC
11-f	AAATGGGTCGCGGATCC**ATG**GTTTCTGAGGAGAATGCTC
11-r	AAGCTTGTCGACGGAGCTC**CTA**GGTTTTTAGATACGTATCGATAATCAATCCTGC
17-f	AAATGGGTCGCGGATCC**ATG**TTCGCCGCCGCCA
17-r	AAGCTTGTCGACGGAGCTC**TCA**GACTGTCGTACGCCCCATG
19-f	AAATGGGTCGCGGATCC**ATG**CCTTCATCAGTACCTGCACCTTC
19-r	AAGCTTGTCGACGGAGCTC**TTA**AGTCATCTGCAAAAGAGTATAATCCG
*Sh*PT1-f	AAATGGGTCGCGGATCC**ATG**GCTGACGCGCACACTCC
*Sh*PT1-r	AAGCTTGTCGACGGAGCTC**CTA**GAGTTTGGGGAGAGGAATGG
*Sh*PT2-f	AAATGGGTCGCGGATCC**ATG**CCTTCAGCTACGACTCC
*Sh*PT2-r	AAGCTTGTCGACGGAGCTC**TTA**GAAGGGCAATTTGAAGGGG

Supplementary Table 5. Primers for cloning carboxylic acid reductase (CAR) candidates

Primer names	Oligonucleotide sequences with adaptors underlined (5′ to 3′)
1-f	AAATGGGTCGCGGATCC**ATG**CTACGCCAACCTTATAC
1-r	AAGCTTGTCGACGGAGCTC**TCA**AATATTCCACCACTTAAGCC
4-f	AAATGGGTCGCGGATCC**ATG**TCACCAACGCCCAGC
4-r	AAGCTTGTCGACGGAGCTC**CTA**GAAGCTCCCTAACAG
6-f	AAATGGGTCGCGGATCC**ATG**TCCTCTCGTGCCAGCTC
6-r	AAGCTTGTCGACGGAGCTC**TCA**TTTCCTGTCAACCTGG
9-f (for *Bv*CAR)	AAATGGGTCGCGGATCC**ATG**GCGTCCAAGCCGTAT
9-r (for *Bv*CAR)	AAGCTTGTCGACGGAGCTC**CTA**CTGCAGCAACCCAAC
10-f	AAATGGGTCGCGGATCC**ATG**AAAGTAGTCCCCATCCC
10-r1	AAGCTTGTCGACGGAGCTC**CTA**CAGCAGAGGACTCTCCCC
10-r2	AAGCTTGTCGACGGAGCTC**TCA**CAGCTCTAAATACCCCCG
18-f	AAATGGGTCGCGGATCC**ATG**TCTTCCTCCTCTCTGAATC
18-r	AAGCTTGTCGACGGAGCTC**TCA**AAGCAATAATCCGACCTT

Supplementary Table 6. Primers for cloning aldehyde reductase (AR) candidates

Primer names	Oligonucleotide sequences with adaptors underlined (5′ to 3′)
1-f	AAATGGGTCGCGGATCC**ATG**ACCTCGAACAACGTGAAC
1-r	AAGCTTGTCGACGGAGCTC**TCA**GAAGATAACGTTCATAGGCC
3-f	AAATGGGTCGCGGATCC**ATG**GCCGACCGTCGTATTC
3-r	AAGCTTGTCGACGGAGCTC**TTA**GACATGACCTATCAAGGGC
6-f	AAATGGGTCGCGGATCC**ATG**AGTTACGGCTCTTCGCAGTGG
6-r	AAGCTTGTCGACGGAGCTC**TCA**ATGACCAACAATGAGCACCG
7-f	AAATGGGTCGCGGATCC**ATG**TCGTCCCCAAGTCAAGTC
7-r	AAGCTTGTCGACGGAGCTC**TCA**GTTGATTCGGAGTGCTTG
12-f (for *Bv*AR3)	AAATGGGTCGCGGATCC**ATG**GCTAGTCGTCGCGTC
12-r (for *Bv*AR3)	AAGCTTGTCGACGGAGCTC**TTA**GATGTGGCCAATGATACGG
14-f (for *Bv*AR2)	AAATGGGTCGCGGATCC**ATG**ACTTCTTTCCCTACTCGC
14-r (for *Bv*AR2)	AAGCTTGTCGACGGAGCTC**CTA**CAAGGCAGGCGTGTCTG
16-f (for *Bv*AR1)	AAATGGGTCGCGGATCC**ATG**TCCAAGCTCGAAGTCAAC
16-r (for *Bv*AR1)	AAGCTTGTCGACGGAGCTC**TCA**GTGGCCAATGACTCGGAC
17-f	AAATGGGTCGCGGATCC**ATG**GCCAACTGTCGCATG
17-r	AAGCTTGTCGACGGAGCTC**CTA**GACATGACCTATCAATATCATC

Supplementary Table 7. Primers for reconstruction of the vibralactone pathway by overlapping PCR

Primer names	Oligonucleotide sequences with homology arms underlined and ribosome binding site marked in red (5′ to 3′)
VibP1-f	CAGCAAATGGGTCGCGGATCC**ATG**GCCGACGCACACACCCCACTTATC
VibP1-r	CCATGGTATATTCCTCCTAGATCC**CTA**GAGTTTTGGAAGTGGAATAGC
*Bv*CAR-f	CTAGGGATCTAGGAGGAATATACC**ATG**GCGTCCAAGCCGTATTTC
*Bv*CAR-r	CATGTCTATCCTCCTACTACTA**CTA**CTGCAGCAACCCAACTTTC
Sfp-f	GTAGTAGTAGGAGGATAGAC**ATG**AAAATCTATGGCATTTAC
Sfp-r	CATGTCTGTACTCCTAGGACTCG**TTA**CAGCAGTTCTTCGTAGC
*Bv*AR1-f	CGAGTCCTAGGAGTACAGAC**ATG**TCCAAGCTCGAAGTCAAC
*Bv*AR1-r	AAGCTTGTCGACGGAGCTC**TCA**GTGGCCAATGACTCGGAC
VibC-f	GAGCTCCGTCGACAAGCTTGCTAATACGACTCACTATAGG
VibC-r	ATATCTCCTTTTATTAGAGCTC**TTA**CTGGGGTGTCTTGCTG
VibO-f	GCTCTAATAAAAGGAGATATACC**ATG**GCTGCTGTTGAAAAC
VibO-r	GTGGTGGTGCTCGAGTGCGGCC**TTA**ATAAGAACCCTCAGTCGAC

Supplementary Methods

All the structural diagrams were prepared using the program PyMOL (http://www.pymol.org/) and CorelDRAW Graphics Suite. Bar graphs and spectral plots were constructed by Graphpad Prism 9. NMR data were collected by Bruker TopSpin and analyzed by MestReNova. ChemBioDraw 14.0 was used for drawing chemical structures.

Supplementary Method 1. Activity-guided fractionation and proteomic analysis

Approximately 60 g of frozen mycelia were powdered in liquid N_2 in the presence of silicon dioxide, successively homogenized by adding 100 mL of 25 mM Hepes (N-(2-hydroxyethyl) piperazine-N′-(2-ethanesulfonic acid)) buffer (pH 7.5). The following operations were carried out at 4 °C. The slurry was centrifuged for 20 min at 12,000 rpm. After centrifugation, ammonium sulfate was added to the supernatant and the final concentration was 40% saturation. The suspension was left for 1 h and centrifuged at 12,000 rpm for 15 min. The pellet was dissolved in 25 mM Hepes (pH 7.5) buffer. The undissolved residue was removed by centrifugation. The supernatant sample was desalted and applied to a DEAE-Sepharose FF column equilibrated with 25 mM Hepes (pH 7.5) buffer, and eluted stepwise with NaCl at 0.1, 0.2, 0.3, 0.4, 0.7, 1.0 M. The 0.4 M NaCl fraction showing the enzyme activity (Supplementary Fig. 1) was collected and changed buffer to 20 mM Tris-HCl (pH 7.3) and concentrated with a 10 kDa cut-off Centriprep 10 (Millipore) up to 3 mL.

Enzyme fraction was conducted on ÄKTApure (GE Healthcare) at 7 °C and data were collected by Unicorn 6.3. The concentrated sample was centrifuged (10,000 × *g* for 10 min) and applied to a Mono Q column (5/50 GL) (GE Healthcare, 1 mL) equilibrated with 20 mM Tris-HCl (pH 7.3). The column was eluted with buffer A (20 mM Tris-HCl, pH 7.3) and buffer B (20 mM Tris-HCl, 1 M NaCl, pH 7.3) at 0.8 $mL·min^{-1}$, sequentially in buffer B 4% 5 mL, 8% 6 mL, 12% 8 mL, 14% 6 mL, 16% 8 mL, 18% 6 mL, 20% 6 mL, and 100% 12 mL. The fractions having the enzyme activity were desalted, replaced with 50 mM PBS (Na_2HPO_4, NaH_2PO_4, pH 7.0) buffer containing 2 M $(NH_4)_2SO_4$, and applied to a hydrophobic interaction chromatography (HIC) (Resource PHE 1 mL, GE Healthcare) equilibrated with the same buffer. The column was eluted with buffer A (50 mM PBS, 2 M $(NH_4)_2SO_4$, pH 7.0) and buffer B (50 mM PBS, pH 7.0) at 1.0 $mL·min^{-1}$, sequentially in buffer A from 100% to 50% for 5 mL, and then to 0% for 15 mL. Fractions having the enzyme activity were desalted and stored at -80 °C. The above steps were repeated 22 times to obtain enough proteins (from approximately 1,320 g of mycelia). The sample combined from the same fraction was concentrated up to 0.5 mL with a Centriprep 10 (Millipore) and applied to a Superdex 75 10/300 GL (24 mL, GE Healthcare) and eluted with 25 mM Hepes (pH 7.5) buffer containing 0.15 M NaCl at 0.5 $mL·min^{-1}$. The fraction containing the enzyme activity was concentrated up to 0.5 mL, applied to a Superdex 200 Increase 10/300GL (24 mL, GE Healthcare), and eluted at 0.75 $mL·min^{-1}$ in the same conditions used for the Superdex 75. Fractions showing the enzyme activity were desalted, concentrated, and freeze-dried in vacuum at -55 °C for the next step of protein identification.

The protein sample was dissolved and subjected to SDS-PAGE electrophoresis. The resulting gel bands were added with 10 mM DTT (dithiothreitol)/125 mM NH_4HCO_3 solution to carry out reduction at 56 °C for 30 min. The supernatant was removed by centrifugation. 55 mM IAM (iodoacetamide)/125 mM NH_4HCO_3 solution was added to the sample and kept in the dark for 30 min at room temperature. The gel bands were washed twice with 50% acetonitrile solution before drying the gel pieces in a vacuum centrifuge. Vacuum-dried gel pieces were added trypsin in 25 mM NH_4HCO_3 buffer (pH 8.0) and left to incubate overnight at 37 °C. Peptide extraction was performed three times with 0.5% formic acid in 50% acetonitrile. The entire supernatant was collected and then completely vacuum-dried. The peptide was subjected to shotgun proteomic analysis using nano liquid chromatography (*ekspert™ nanoLC*) on a quadrupole time-of-flight instrument (AB SCIEX Triple TOF™ 5600 plus). The mass spectrometry data were submitted to the Proteinpilot software connected to the AB SCIEX Triple TOF^{TM} 5600 plus mass spectrometer for database retrieval. The proteome data were analyzed against an annotated database of the *B. vibrans* draft genome (unpublished) to identify proteins.

Supplementary Method 2. Expression and purification of recombinant enzymes.

Single colony was used to inoculate 5 ml Luria-Bertani (LB) medium containing 50 μg·mL^{-1} kanamycin and grown overnight at 37 °C at 220 rpm. The overnight culture was diluted (1:50 or 100) with fresh LB medium of the same antibiotic selection and incubated at 37 °C until the optical density at 600 nm (OD_{600}) reached 0.6~0.8. The *E. coli* cells were induced with 0.1 mM isopropyl-β-D-thiogalactopyranoside (IPTG) for expression of VibO and AR candidates (0.01 mM IPTG for CAR), followed by further incubation for 24 h at 16 °C. For expression of prenyltransferase candidates, 0.2 mM IPTG was added and cultured for 15 h at 25 °C. Cells were harvested by centrifugation at 8,000 rpm at 4 °C for 2 min, washed with deionized water and suspended in 50 mM sodium phosphate buffer pH 7.5 for VibO candidates and variants, followed by sonication on ice. Lysed cells were centrifuged at 12,000 rpm for 5 min at 4 °C to remove cellular debris. The cleared lysate was used as crude enzymes for in vitro activity assays. Purification of His-tagged proteins was performed in 25 mM Tris-HCl buffer pH 7.5 with Ni-NTA Agarose resin by (i) binding with addition of imidazole (10 mM) and NaCl (0.3 M), (ii) washing with 75 mM imidazole and 0.5 M NaCl, and (iii) elution with 200 mM imidazole and 0.5 M NaCl. The elute containing the purified enzyme was immediately desalted with 50 mM sodium phosphate buffer pH7.5 and concentrated with a 10 kDa cut-off Centriprep 10 (Millipore). The purified proteins were instantly assayed for enzyme activity and analyzed by SDS-PAGE. Storage may abolish the VibO enzyme activity. The induced *E. coli* cells harboring AR candidates were suspended in 50 mM MES buffer pH 6.2. For CAR candidates 50 mM Tris-HCl buffer pH 7.5 containing 10 mM $MgCl_2$, 1 mM EDTA, 1 mM DTT and 10% glycerol was used. Purification of His-tagged proteins was performed as described above but desalted with their respective buffers.

Supplementary Method 3. VibO protein for crystallization

The DNA encoding VibO was ligated into the vector pET28a-T7-3C (a modified version of pET28a vector containing a N-terminal His_6-tag, T7-tag with HRV 3C site). The recombinant plasmid was transferred into *E. coli* BL21 (DE3) cells for protein expression. Single colony was used to inoculate 10 mL LB medium containing 50 μg·mL^{-1} kanamycin and grown overnight at 37 °C at 220 rpm. Each 100 mL of fresh LB medium with the same antibiotic selection was inoculated with 1 mL of the overnight culture and incubated at 37 °C until the optical density at 600 nm (OD_{600}) reached 0.4~0.6. Protein expression was induced with 0.1 mM IPTG, followed by further incubation for 20 h at 16 °C. *E. coli* cells were harvested by centrifugation at 3,500 rpm at 4 °C for 10 min, re-suspended in the binding buffer (50 mM Tris, 500 mM NaCl, 5 mM imidazole at pH 7.9), and then lysed by the ultrahigh pressure homogenizer FB-110XNANO homogenizer machine (Shanghai Litu Machinery Equipment Engineering Co., Ltd.). Then the lysis was spun down by centrifuge at 17000 rpm for 30 minutes to remove the pellets fractions. His_6-tagged proteins were purified by Ni^{2+}-NTA agarose (GE Healthcare) affinity chromatography with elution buffer (25 mM Hepes Buffer, pH 7.5, 0.5 M NaCl, 200 mM imidazole).

The sedimentation velocity analysis was performed on a Beckman XL-I analytical ultracentrifuge equipped with an eight-cell rotor under 42000 rpm at 20 °C. The partial specific volume of VibO protein sample and the buffer density were calculated using the program SEDNTERP (http://www.rasmb.org/). The final sedimentation velocity data were analyzed and fitted to a continuous sedimentation coefficient distribution model using the program SEDFIT[25]. The fitting results were further output to the Origin 9.0 software.

Supplementary Method 4. Chemical synthesis

Synthesis of **5a′**, **5a**, and **4a**

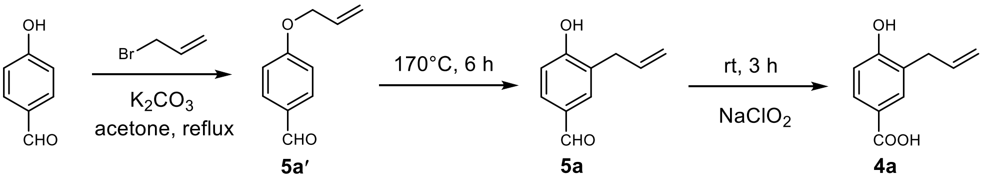

Synthesis of 4-allyloxybenzaldehyde (**5a′**)[26]

4-hydroxybenzaldehyde (4.9 g, 40 mmol), dried acetone (50 mL) and benzyltriphenylphosphonium bromide as a phase transfer catalyst (0.430 g) were sequentially added to the flask (50 mL). The solid K_2CO_3 (16.6 g, 120 mmol) was vigorously stirred and refluxed at 56 °C for 15 min before allyl bromide (6 mL, 68 mmol) was added dropwise. Stirring was continued at 56 °C for 6 h and then cooled to room temperature. After full consumption of the starting material, the resulting mixture was regulated to pH < 3 by the addition of 3 M HCl. The precipitation was filtered and the filtrate was extracted with ethyl acetate (10 mL) three times. The combined organic extracts were concentrated under reduced pressure and followed by normal-phase chromatographic purification (Silica gel, petroleum ether/ethyl acetate = 45/1 to 40/1, v/v) to afford a colorless oil **5a′** (5.5 g, 84.6%). ^{1}H NMR (500 MHz, $CDCl_3$): δ 9.80 (s, 1H), 7.75 (d, *J* = 8.8 Hz, 2H), 6.94 (d, *J* = 8.7 Hz, 2H), 5.98 (m, 1H), 5.37 (dd, *J* = 17.3, 1.4 Hz, 1H), 5.26 (dd, *J* = 10.5, 1.4 Hz, 1H), 4.54 (d, *J* = 5.3 Hz, 2H). ^{13}C NMR (126 MHz, $CDCl_3$): δ 190.4, 163.3, 132.0, 131.6, 129.7, 117.9, 114.7, 68.6 (Supplementary Fig. 57).

Synthesis of 3-Allyl-4-hydroxybenzaldehyde (**5a**)[27]

A 50 mL reaction vessel containing the purified colorless oil **5a′** (3.5 g, 21.6 mmol) and a Teflon-coated magnetic stir bar was immersed in an oil bath that had been preheated to 170 °C. The reaction mixture was stirred and heated at 170 °C for 6 h with a reflux condenser. The resulting crude product was cooled to room temperature and subsequently purified by column chromatography (Silica gel, petroleum ether/ethyl acetate = 8/1 to 6/1, v/v) to obtain the title compound **5a** (1.21 g, 34.6%) as a white powder. ^{1}H NMR (500 MHz, $CDCl_3$): δ 9.84 (s, 1H), 7.69 (m, 2H), 7.07 (s, 1H), 6.97 (d, *J* = 8.1 Hz, 1H), 6.09–5.93 (m, 1H), 5.18 (t, *J* = 1.4 Hz, 1H), 5.17–5.13 (m, 1H), 3.47 (d, *J* = 6.5 Hz, 2H). ^{13}C NMR (126 MHz, $CDCl_3$): δ 191.9, 160.5, 135.4, 132.7, 130.9, 129.6, 126.9, 117.1, 116.1, 34.4 (Supplementary Fig. 58).

Synthesis of 3-Allyl-4-hydroxybenzoic acid (**4a**)

5a (1.2 g, 7.4 mmol) was dissolved into 17.669 mL THF: H_2O: t-BuOH (4:1:1) mixture in a 50 mL round-bottom flask. 2-Methyl-2-butene (1.5 mL) was added followed by 1.378 g NaH_2PO_4 (8.83 mmol) and 798 mg $NaClO_2$ (8.83 mmol). After being stirring at room temperature for 3 h, a homogeneous solution was obtained and quenched by the addition of saturated NH_4Cl solution. The reaction mixture was extracted with ethyl acetate three times (3 × 30 mL). The combined organic layer was evaporated under reduced pressure for crude residue, followed by purification over flash chromatography (petroleum ether/ethyl acetate = 25/1 to 20/1, v/v) to afford **4a** (550 mg, 41.8%) as a white powder. ^{1}H NMR (500 MHz, MeOD): δ 7.74 (d, *J* = 2.1 Hz, 1H), 7. 71 (dd, *J* = 8.4, 2.2 Hz, 1H), 6.78 (d, *J* = 8.4 Hz, 1H), 5.97 (m, 1H), 5.02 (m, 2H), 3.34 (d, *J* = 6.6 Hz, 2H). ^{13}C NMR (126 MHz, MeOD): δ 170.3, 161.0, 137.7, 133.1, 130.8, 127.9, 122.5, 115.9, 115.4, 35.0 (Supplementary Fig. 59).

Synthesis of 4-((3-methylbut-2-en-1-yl)oxy)benzaldehyde (**5′**)[28]

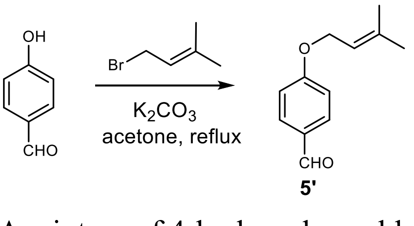

A mixture of 4-hydroxybenzaldehyde (0.61 g, 5 mmol), benzyltriphenylphosphonium bromide (0.115 g, 1-

3% M) and K_2CO_3 (1.83 g, 15 mmol) in anhydrous acetone (15 mL) was stirred and refluxed at 56 °C for 15 min. 3,3-dimethylallyl bromide (1.175 mL, 10 mmol) was added dropwise to the mixture. Stirring was continued at 56 °C for an additional 6 h. The reaction solution was quenched by dropwise addition of 3 M HCl until pH < 3. The resulting mixture was filtered and then extracted with ethyl acetate (3 × 20 mL). The filtrate was evaporated and subjected to silica gel column eluted with petroleum ether/ethyl acetate (with a ratio of 45/1, v/v) to give **5′** (779 mg, 82%) as a colorless oil. ^{1}H NMR (500 MHz, $CDCl_3$): δ 9.81 (s, 1H), 7.76 (m, 1H), 7.75 (m, 1H), 6.95 (m, 1H), 6.93 (m, 1H), 5.45–5.39 (m, 1H), 4.53 (d, *J* = 6.7 Hz, 2H), 1.74 (s, 3H), 1.70 (s, 3H). ^{13}C NMR (126 MHz, $CDCl_3$): δ 190.6, 163.9, 138.9, 131.9, 129.7, 118.8, 114.8, 65.1, 25.7, 18.2 (Supplementary Fig. 60).

Synthesis of 4-((3-methylbut-2-en-1-yl)oxy)benzoic acid (**4′**)[28]

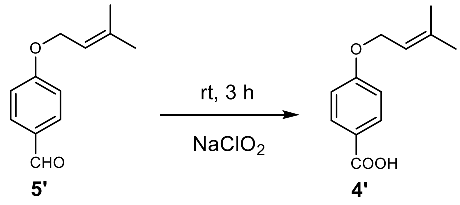

5′ (200 mg, 1.05 mmol) with corresponding mixture THF: H_2O: t-BuOH (4:1:1, 2.256 mL) was added to a reaction vial (25 mL) containing a magnetic stir bar. 2-methyl-2-butene (210 µL), NaH_2PO_4 (0.197 mg, 1.26 mmol) and $NaClO_2$ (114 mg, 1.26 mmol) were added successively. The resulting mixture was stirred at room temperature for 3 h before the reaction was quenched with saturated aqueous NH_4Cl solution and extracted with ethyl acetate (3 × 10 mL). The combined organic solvents were removed under reduced pressure to provide the crude residue, which was separated by silica gel column chromatography (petroleum ether/ethyl acetate = 45/1, 40/1, 30/1, v/v) for a white powder **4′** (90 mg, 41.6%). ^{1}H NMR (500 MHz, $CDCl_3$): δ 8.07–8.05 (m, 1H), 8.05–8.04 (m, 1H), 6.97–6.95 (m, 1H), 6.95–6.93 (m, 1H), 5.51–5.47 (m, 1H), 4.59 (d, *J* = 6.8 Hz, 2H), 1.81 (s, 3H), 1.76 (s, 3H). ^{13}C NMR (126 MHz, $CDCl_3$): δ 171.5, 163.5, 139.1, 132.8, 121.6, 119.1, 114.5, 65.2, 26.0, 18.4 (Supplementary Fig. 61).

Synthesis of 4-hydroxy-3-(3-methylbut-2-en-1-yl)benzaldehyde (**5**)[28]

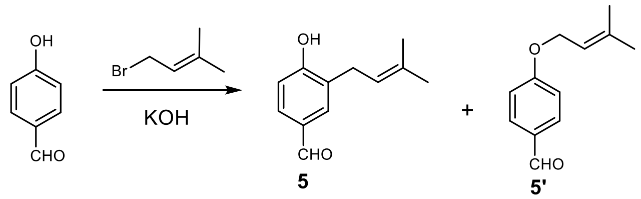

1.120 g KOH (20 mmol) was subjected to 20 mL water in a vial (50 mL). When the reaction was cooled to 0 °C in an ice bath, 4-hydroxybenzaldehyde (1.237 g, 10 mmol), 3,3-dimethylallyl bromide (2.4 mL, 20 mmol) were added in sequence. After stirring at this temperature for 4 h, the reaction mixture was acidified with HCl (3 M) to pH < 3.0, and then extracted with ethyl acetate (10 mL) three times. The organic fractions were combined and concentrated. The crude residue was purified by flash silica gel column chromatography (petroleum ether/ethyl acetate = 45/1 to 6/1, v/v) to access the product **5**, a colorless oil (249.7 mg, 6.6%). ^{1}H NMR (400 MHz, $CDCl_3$): δ 9.81 (s, 1H), 7.73–7.62 (m, 2H), 7.42 (s, 1H), 6.96 (d, *J* = 8.2 Hz, 1H), 5.34 (t, *J* = 7.2 Hz, 1H), 3.41 (d, *J* = 7.2 Hz, 2H), 1.78 (s, 3H), 1.76 (s, 3H). ^{13}C NMR (101 MHz, $CDCl_3$): δ 192.0, 160.8, 135.1, 132.1, 130.6, 129.4, 128.5, 120.9, 115.9, 28.8, 25.8, 17.9. HRMS (ESI-QTOF) calcd. for $C_{12}H_{14}O_3$ $[M-H]^-$: 189.0921, found 189.0924 (Supplementary Figs. 47 and 62).

Synthesis of 4-hydroxy-3-(3-methylbut-2-en-1-yl)benzylalcohol (**6**)[28]

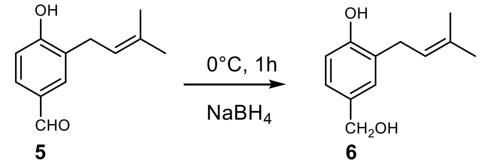

5 (38 mg) dissolved in 2 mL of MeOH at 0 °C was mixed with $NaBH_4$ (7.56 mg). The reaction mixture was cooled in an ice bath for 1 h at dark, and then extracted with EtOAc. **6** (18 mg) was purified from the extract as colorless oil, $C_{10}H_{16}O_2$, ESI-MS *m/z*: 215 $[M + Na]^+$. ^{1}H-NMR ($CHCl_3$, 400 MHz) δ: 7.14 (1H, s), 7.14 (1H, d, *J* = 7.5 Hz), 6.81 (1H, d, *J* = 7.5 Hz), 5.35 (1H, t, *J* = 7.0 Hz), 4.61 (2H, s), 3.39 (1H, d, *J* = 7.0 Hz), 1.80 (6H, s); ^{13}C-NMR ($CHCl_3$, 100 MHz) δ: 153.9, 134.9, 133.0, 129.2, 127.1, 126.7, 121.5, 115.7, 65.2, 29.7, 25.8, 17.9

Synthesis of 4-hydroxy-3-(3-methylbut-2-en-1-yl)benzoic acid (**4**)[28]

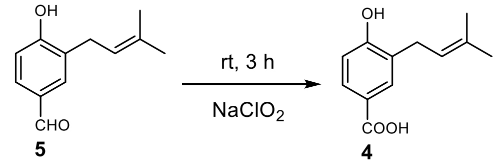

5 (130 mg, 0.68 mmol) was dissolved into 1.642 mL mixture containing THF: H_2O: t-BuOH (4:1:1) in a round bottom flask. 2-Methyl-2-butene (137 μL), NaH_2PO_4 (128 mg, 0.82 mmol) and $NaClO_2$ (74 mg, 0.82 mmol) were added in succession. Once the addition was complete, the resulting mixture was stirred at ambient temperature and the reaction was monitored by TLC. When most conversion was reached (ca. 3 h), saturated NH_4Cl solution was added to quench the reaction, which was extracted with ethyl acetate (3 × 10 mL). The organic layers were combined and the solvent was evaporated under reduced pressure. The desired white powder **4** (20 mg, 14.3%) was purified from concentrated residue by flash chromatography (petroleum ether/ethyl acetate = 25/1, 20/1, 15/1, v/v). ^{1}H NMR (500 MHz, $CDCl_3$): δ 7.90 (m, 2H), 6.85 (d, *J* = 9.0 Hz, 1H), 5.35–5.29 (m, 1H), 3.40 (d, *J* = 7.1 Hz, 2H), 1.79 (s, 6H). ^{13}C NMR (126 MHz, $CDCl_3$): δ 171.7, 159.5, 136.0, 132.7, 130.7, 127.0, 121.8, 121.1, 115.8, 29.8, 26.0, 18.1. HRMS (ESI-QTOF) calcd. for $C_{12}H_{14}O_3$ $[M-H]^-$: 205.0870, found 205.0870 (Supplementary Figs. 46 and 63).

Synthesis of (4-((3-methylbut-2-en-1-yl)oxy)phenyl)methanol (**6′**)[28]

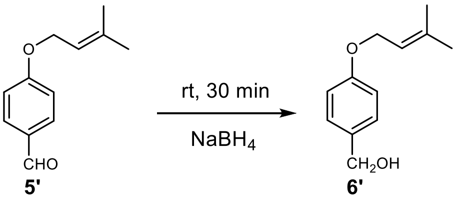

A solution of **5′** (125 mg, 0.65 mmol) in 1 mL MeOH was charged with $NaBH_4$ (0.038 g, 0.981 mmol) and stirred at room temperature for 30 min. The reaction mixture was extracted with ethyl acetate three times (3 × 10 mL). The organic layers were combined and evaporated under reduced pressure. Purification of the crude residue by column chromatography (petroleum ether/ethyl acetate = 8/1) afforded the compound **6′** (30 mg, 24%) as a white powder. ^{1}H NMR (500 MHz, $CDCl_3$): δ 7.26 (d, *J* = 8.7 Hz, 2H), 6.89 (d, *J* = 8.7 Hz, 2H), 5.52–5.47 (m, 1H), 4.57 (s, 2H), 4.50 (d, *J* = 6.7 Hz, 2H), 1.80 (s, 3H), 1.74 (s, 3H). ^{13}C NMR (126 MHz, $CDCl_3$): δ 158.5, 138.3, 133.2, 128.7, 119.7, 114.8, 65.0, 64.9, 25.9, 18.3 (Supplementary Fig. 64).

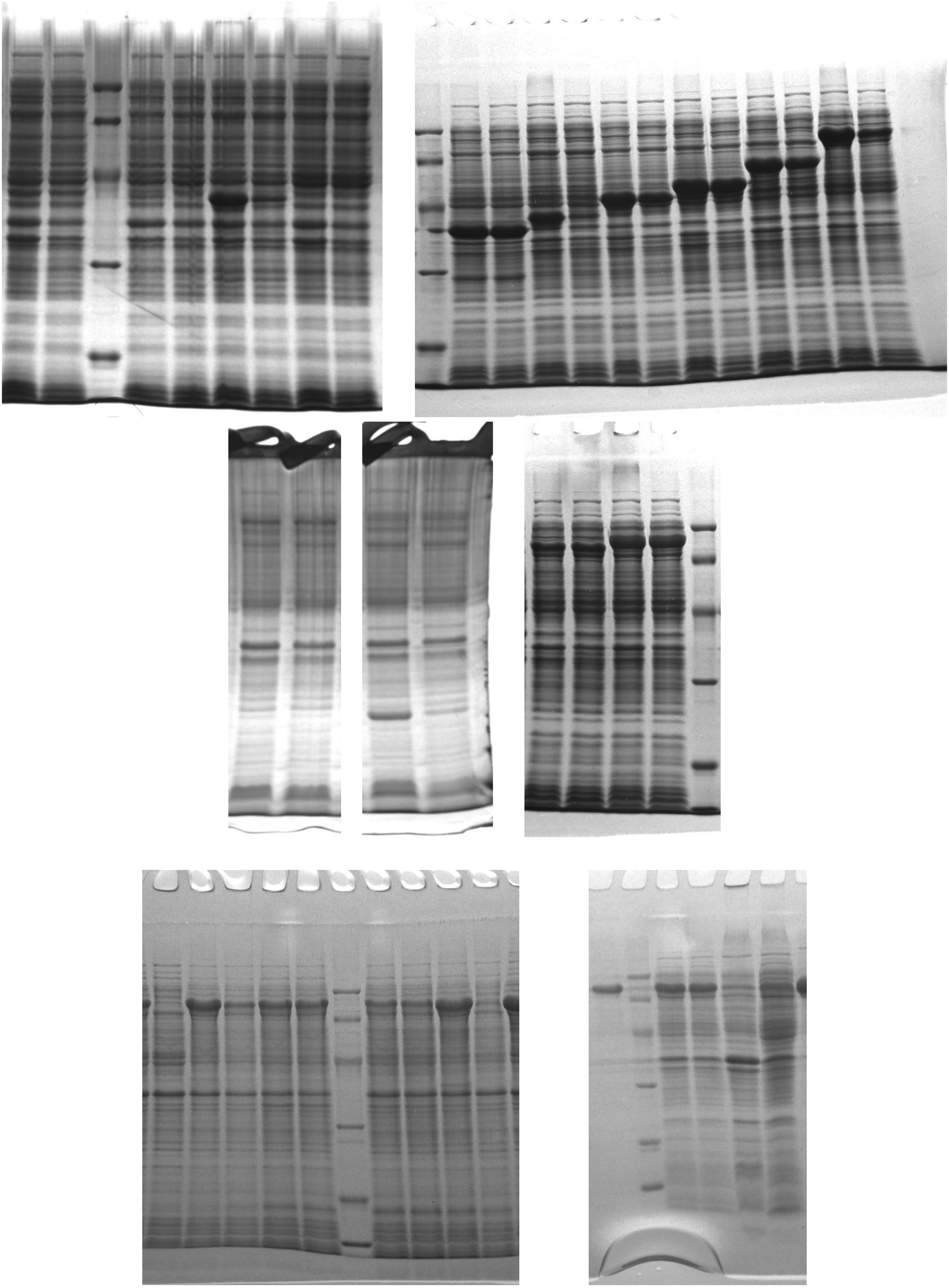

The original SDS-PAGE gels in Supplementary Figure 3

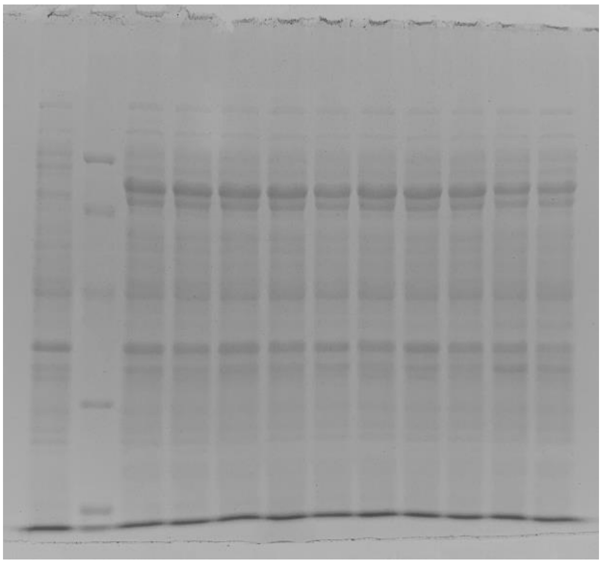

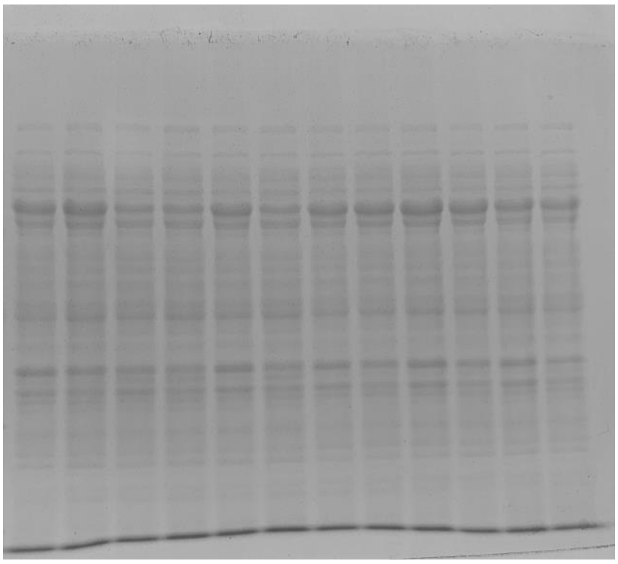

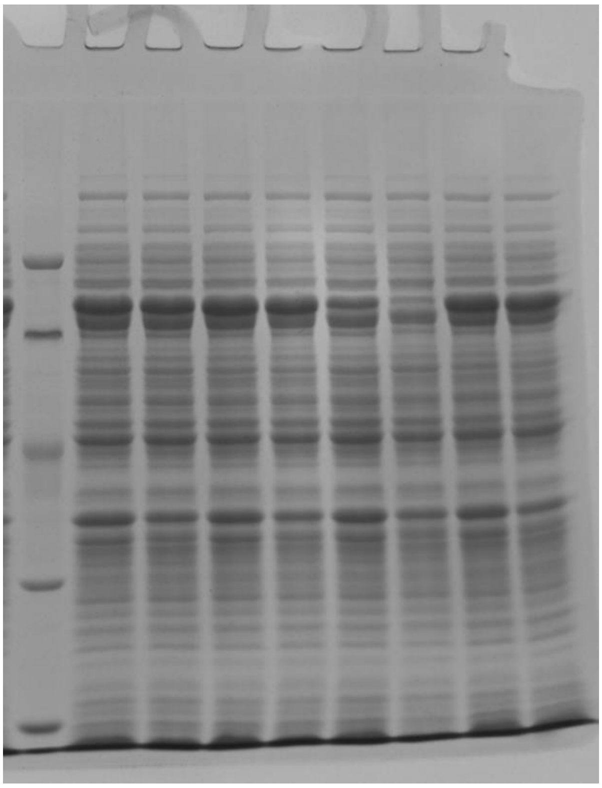

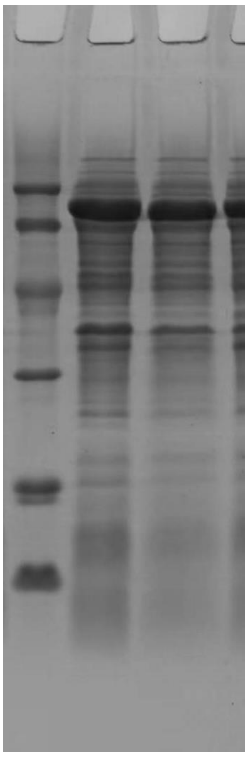

The original SDS-PAGE gels in Supplementary Figure 30

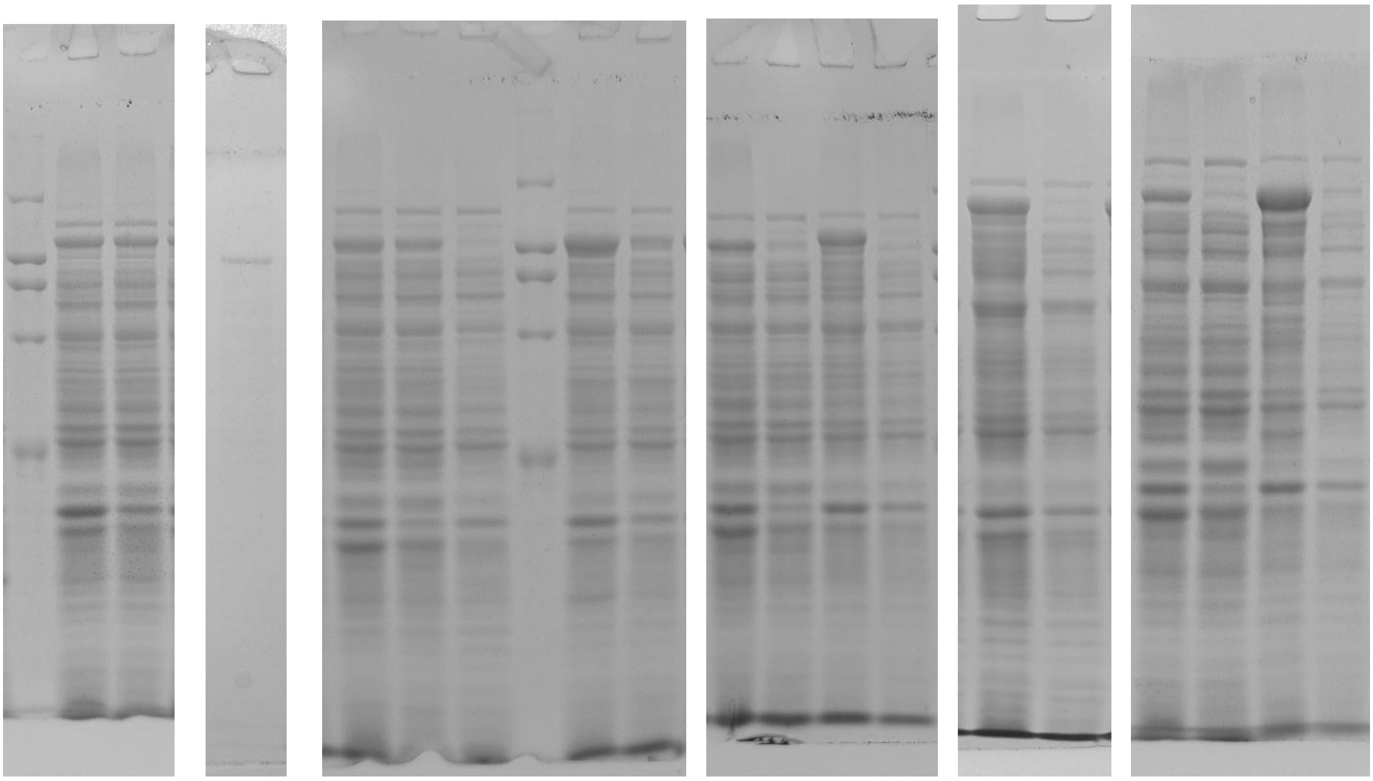

The original SDS-PAGE gels in Supplementary Figure 52

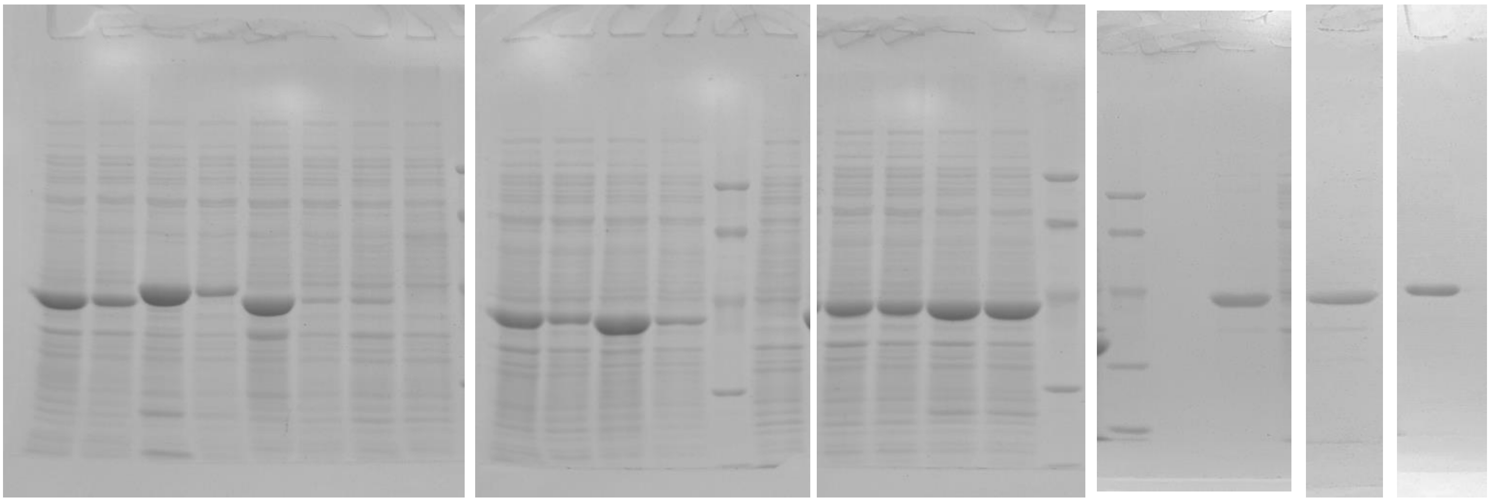

The original SDS-PAGE gels in Supplementary Figure 54

Supplementary References

1. Jiang, M.Y. et al. Derivatives of vibralactone from cultures of the basidiomycete *Boreostereum vibrans*. *Chem. Pharm. Bull.* **56**, 1286–1288 (2008).
2. Liu, D.Z. et al. Vibralactone: A lipase inhibitor with an unusual fused *β*-lactone produced by cultures of the basidiomycete *Boreostereum vibrans*. *Org. Lett.* **8**, 5749–5752 (2006).
3. Morris, R. A. C., Ewing D. F., Whipps J. M. & Coley-Smith J. R. Antifungal hydroxymethyl-phenols from the mycoparasite *Verticillium biguttatum*. *Phytochemistry* **39**, 1043–1048 (1995).
4. Torres Pazmiño, D. E., Baas, B. J., Janssen, D. B. & Fraaije, M. W. Kinetic mechanism of phenylacetone monooxygenase from *Thermobifida fusca*. *Biochemistry* **47**, 4082-4093 (2008).
5. Fordwour, O. B., Luka, G., Hoorfar, M. & Wolthers, K. R. Kinetic characterization of acetone monooxygenase from *Gordonia* sp. strain TY-5. *AMB Express*. **8**, 181 (2018).
6. Teufel, R. et al. Flavin-mediated dual oxidation controls an enzymatic Favorskii-type rearrangement. *Nature* **503**, 552–556 (2013).
7. Lv, J.-M. et al. Biosynthesis of biscognienyne B involving a cytochrome P450-dependent alkynylation. *Angew. Chem. Int. Ed*. **59**, 13531–13536 (2020); *Angew. Chem.* **132**, 13633–13638 (2020).
8. Young, G., Gibson, F., Leppik, R., A. & Hamilton, J. A. Biochemical and genetic studies on ubiquinone biosynthesis in *Escherichia coli* K-12: 4-hydroxybenzoate octaprenyltransferase. *J. Bacteriol*. **110**, 18–25 (1972).
9. Siebert, M. et al. Ubiquinone biosynthesis. Cloning of the genes coding for chorismate pyruvate-lyase and 4-hydroxybenzoate octaprenyl transferase from *Escherichia coli*. *FEBS Lett*. **307**, 347–350 (1992).
10. He, B.-B. et al. Combinatory biosynthesis of prenylated 4-hydroxybenzoate derivatives by overexpression of the substrate-promiscuous prenyltransferase XimB in engineered *E. coli*. *ACS Synth. Biol.* **7**, 2094–2104 (2018).
11. Yazaki, K., Kunihisa, M., Fujisaki, T. & Sato, F. Geranyl diphosphate: 4-hydroxybenzoate geranyltransferase from *Lithospermum erythrorhizon*, cloning and characterization of a key enzyme in shikonin biosynthesis. *J. Biol. Chem.* **277**, 6240–6246 (2002).
12. Bai, N. et al. An aromatic prenyltransferase involved in the biosynthesis of vibralactone from *Stereum vibrans*. *Appl. Environ. Microbiol.* **86**, e02687–19 (2020).
13. Braesel, J., Fricke, J., Schwenk, D. & Hoffmeister, D. Biochemical and genetic basis of orsellinic acid biosynthesis and prenylation in a stereaceous basidiomycete. *Fungal Genet. Biol.* **98**, 12–19 (2017).
14. Ling, J.-G. et al. A functionally-distinct carboxylic acid reductase PcCAR4 unearthed from a repertoire of type IV CARs in the white-rot fungus *Pycnoporus cinnabarinus*. *J. Biotechnol.* **307**, 55–62 (2020).
15. Winkler,M. & Winkler, C. K. *Trametes versicolor* carboxylate reductase uncovered. *Monatshefte Fur Chemie* **147**, 575–578 (2016).
16. Schwendenwein, D. et al. Selective enzymatic transformation to aldehydes in vivo by fungal carboxylate reductase from *Neurospora crassa*. *Adv. Synth. Catal.***358**, 3414–3421 (2016).
17. Li, C. et al. Biosynthesis of LL-Z1272β: Discovery of a new member of NRPS-like enzymes for aryl-aldehyde formation. *ChemBioChem* **17**, 904–907 (2016).
18. Zheng, L. et al. Ustethylin biosynthesis implies phenethyl derivative formation in *Aspergillus ustus*. *Org. Lett.* **22**, 7837–7841 (2020).
19. Kunjapur, A. M., Cervantes, B. & Prather, K. L. J. Coupling carboxylic acid reductase to inorganic pyrophosphatase enhances cell-free *in vitro* aldehyde biosynthesis. *Biochem. Eng. J.* **109**, 19–27 (2016).
20. Yang, D., François, J. M. & de Billerbeck, G. M. Cloning, expression and characterization of an aryl-alcohol dehydrogenase from the white-rot fungus *Phanerochaete chrysosporium* strain BKM-F-1767. *BMC Microbiol.* **12**, 126 (2012).
21. Ken, C. F. et al. Biochemical characterization of a functional recombinant aryl-alcohol dehydrogenase from *Taiwanofungus camphorate. Botanical Studies* **55**, 14 (2014).
22. Larroy, C. et al. Characterization of the *Saccharomyces cerevisiae YMR318C (ADH6)* gene product as a broad specificity NADPH-dependent alcohol dehydrogenase: relevance in aldehyde reduction. *Biochem. J.* **361**, 163–172

(2002).
23. Pick, A., Rühmann, B., Schmid, J. & Sieber, V. Novel CAD-like enzymes from *Escherichia coli* K-12 as additional tools in chemical production. *Appl. Microbiol. Biotechnol.* **97**, 5815–5824 (2013).
24. Finnigan, W. et al. Engineering a seven-enzyme biotransformation using mathematical modelling and characterized enzyme parts. *ChemCatChem* **11**, 3474–3489 (2019).
25. Schuck, P. Size-distribution analysis of macromolecules by sedimentation velocity ultracentrifugation and lamm equation modeling. *Biophysical Journal* **78** (3), 1606–1619 (2000).
26. Yang, Y.-L. et al. A monooxygenase from *Boreostereum vibrans* catalyzes oxidative decarboxylation in a divergent vibralactone biosynthesis pathway. *Angew. Chem. Int. Ed.* **55**, 5463–5466 (2016); *Angew. Chem.* **128**, 5553–5556 (2016).
27. Tsai, T. W. et al. A new synthesis of benzofurans from phenols via Claisen rearrangement and ring-closing metathesis. *J. Chin. Chem. Soc-Taip.* **51**, 1307–1318 (2013).
28. Zhao, P.-J. et al. Elucidating the biosynthetic pathway for vibralactone: a pancreatic lipase inhibitor with a fused bicyclic β-lactone. *Angew. Chem. Int. Ed.* **52**, 2298–2302 (2013); *Angew. Chem.* **125**, 2354–2358 (2013)
