# Supplementary material for: A flavin-monooxygenase catalyzing oxepinone formation and the complete biosynthesis of vibralactone
Source: Nat Commun. 2023 Jun 10;14:3436. doi: 10.1038/s41467-023-39108-x (PMC10257657; doi:10.1038/s41467-023-39108-x)
Supplement: Supplementary file 3 — Reporting Summary [file 41467_2023_39108_MOESM3_ESM.pdf]

nature portfolio

Corresponding author(s): Ying Zeng

Last updated by author(s): May 6, 2023

Reporting Summary

Nature Portfolio wishes to improve the reproducibility of the work that we publish. This form provides structure for consistency and transparency in reporting. For further information on Nature Portfolio policies, see our Editorial Policies and the Editorial Policy Checklist.

Statistics

For all statistical analyses, confirm that the following items are present in the figure legend, table legend, main text, or Methods section.

n/a	Confirmed	
☐	☒	The exact sample size (*n*) for each experimental group/condition, given as a discrete number and unit of measurement
☐	☒	A statement on whether measurements were taken from distinct samples or whether the same sample was measured repeatedly
☒	☐	The statistical test(s) used AND whether they are one- or two-sided<br>*Only common tests should be described solely by name; describe more complex techniques in the Methods section.*
☒	☐	A description of all covariates tested
☒	☐	A description of any assumptions or corrections, such as tests of normality and adjustment for multiple comparisons
☐	☒	A full description of the statistical parameters including central tendency (e.g. means) or other basic estimates (e.g. regression coefficient) AND variation (e.g. standard deviation) or associated estimates of uncertainty (e.g. confidence intervals)
☒	☐	For null hypothesis testing, the test statistic (e.g. *F*, *t*, *r*) with confidence intervals, effect sizes, degrees of freedom and *P* value noted<br>*Give P values as exact values whenever suitable.*
☒	☐	For Bayesian analysis, information on the choice of priors and Markov chain Monte Carlo settings
☒	☐	For hierarchical and complex designs, identification of the appropriate level for tests and full reporting of outcomes
☒	☐	Estimates of effect sizes (e.g. Cohen's *d*, Pearson's *r*), indicating how they were calculated

Our web collection on statistics for biologists contains articles on many of the points above.

Software and code

Policy information about availability of computer code

Data collection	LC-MS data were collected by Agilent MassHunter Workstation Data Acquisition. NMR data were collected by Bruker TopSpin. Protein purification data were collected by Unicorn 6.3. Crystals diffraction data were collected at Shanghai Synchrotron Radiation Facility beamline BL17B. Spectral data were monitored by NanoDrop One UV-Vis Spectrophotometer (Thermo Scientific) and BioTek Cytation 1 cell imaging multimode reader.
Data analysis	Agilent MassHunter Qualitative Analysis B.06.00, ChemBioDraw 14.0, MestReNova 6.1.0, Origin 9.0, SEDNTERP, SEDFIT, PHASER, PHENIX 1.17.1, Coot 0.8.9.2, XDS, autoPROC 1.0.5, MolProbity, PyMOL 2.4.0, autodock vina 1.2.2, GraphPad Prism 9.5.0, CorelDRAW Graphics Suite 2020v22.0.0.412, ClustalX 2.1, FastTree 2.1.10, Proteinpilot software connected to the AB SCIEX Triple TOFTM 5600 plus.

For manuscripts utilizing custom algorithms or software that are central to the research but not yet described in published literature, software must be made available to editors and reviewers. We strongly encourage code deposition in a community repository (e.g. GitHub). See the Nature Portfolio guidelines for submitting code & software for further information.

Data

Policy information about availability of data

All manuscripts must include a data availability statement. This statement should provide the following information, where applicable:

- Accession codes, unique identifiers, or web links for publicly available datasets
- A description of any restrictions on data availability
- For clinical datasets or third party data, please ensure that the statement adheres to our policy

The coordinate and structure factor of VibO in complex with FAD solved in this study have been deposited in the Protein Data Bank under the accession code 7YJ0 [https://doi.org/10.2210/pdb7YJ0/pdb]. Sequences have been deposited in GenBank under accession nos. OP484926 [https://www.ncbi.nlm.nih.gov/nuccore/OP484926], ON653009 [https://www.ncbi.nlm.nih.gov/nuccore/ON653009], ON653010 [https://www.ncbi.nlm.nih.gov/nuccore/ON653010], ON653011 [https://www.ncbi.nlm.nih.gov/nuccore/ON653011], ON653012 [https://www.ncbi.nlm.nih.gov/nuccore/ON653012], ON653013 [https://www.ncbi.nlm.nih.gov/nuccore/ON653013] and ON653014 [https://www.ncbi.nlm.nih.gov/nuccore/ON653014], respectively. Other sequences and structures mentioned in the article are XP_007301038.1 [https://www.ncbi.nlm.nih.gov/protein/XP_007301038.1], XP_007301015.1 [https://www.ncbi.nlm.nih.gov/protein/XP_007301015.1], XP_007303462 [https://www.ncbi.nlm.nih.gov/protein/XP_007303462], XP_007299647 [https://www.ncbi.nlm.nih.gov/protein/XP_007299647], WP_015715234.1 [https://www.ncbi.nlm.nih.gov/protein/WP_015715234.1], 5J7X [https://www.rcsb.org/structure/5J7X], 1PN0_A [https://www.rcsb.org/structure/1PN0], 2DKI_A [https://www.rcsb.org/structure/2DKI], 4K5R_A [https://www.rcsb.org/structure/4K5R], 7VWP_A [https://www.rcsb.org/structure/7VWP], 4X4J_A [https://www.rcsb.org/structure/4X4J], QSV12647.1 [https://www.ncbi.nlm.nih.gov/protein/QSV12647.1], ADE22300.1 [https://www.ncbi.nlm.nih.gov/protein/292386119], Q0D1P1.1 [https://www.ncbi.nlm.nih.gov/protein/Q0D1P1.1], AXS67818.1 [https://www.ncbi.nlm.nih.gov/protein/AXS67818.1], AIZ66878.1 [https://www.ncbi.nlm.nih.gov/protein/AIZ66878.1], DAA64700.1 [https://www.ncbi.nlm.nih.gov/protein/DAA64700.1], A0A2U8U2L6.1 [https://www.ncbi.nlm.nih.gov/protein/A0A2U8U2L6.1], B6HN76.1 [https://www.ncbi.nlm.nih.gov/protein/B6HN76.1], 5F9P_A [https://www.rcsb.org/structure/5F9P], AAV52247.1 [https://www.ncbi.nlm.nih.gov/protein/AAV52247.1], AAP69583.1 [https://www.ncbi.nlm.nih.gov/protein/AAP69583.1], C8VE79.1 [https://www.ncbi.nlm.nih.gov/protein/C8VE79.1], 270787 [https://mycocosm.jgi.doe.gov/cgi-bin/dispGeneModel?db=Aspdur1&id=270787], AIE17460.1 [https://www.ncbi.nlm.nih.gov/protein/AIE17460.1], QDO73504.1 [https://www.ncbi.nlm.nih.gov/protein/QDO73504.1], WP_013222574.1 [https://www.ncbi.nlm.nih.gov/protein/WP_013222574.1], BAN13304.1 [https://www.ncbi.nlm.nih.gov/protein/BAN13304.1], 5AEC _A [https://www.rcsb.org/structure/5AEC] and AVI57411.1 [https://www.ncbi.nlm.nih.gov/protein/AVI57411.1]. All data supporting the findings of this study are available in the main text and the supplementary information. Source data are provided with this paper.

Human research participants

Policy information about studies involving human research participants and Sex and Gender in Research.

Reporting on sex and gender	It is not relevant to this study.
Population characteristics	It is not relevant to this study.
Recruitment	It is not relevant to this study.
Ethics oversight	It is not relevant to this study.

Note that full information on the approval of the study protocol must also be provided in the manuscript.

Field-specific reporting

Please select the one below that is the best fit for your research. If you are not sure, read the appropriate sections before making your selection.

☒ Life sciences ☐ Behavioural & social sciences ☐ Ecological, evolutionary & environmental sciences

For a reference copy of the document with all sections, see nature.com/documents/nr-reporting-summary-flat.pdf

Life sciences study design

All studies must disclose on these points even when the disclosure is negative.

Sample size	No statistical method was used to predetermine sample size in this work. Replicates of n≥2 were chosen for in vitro enzyme activity assays, SDS-PAGE experiments and pathway reconstruction by one-pot and in E.coli, as these are sufficient for us to assess the result.
Data exclusions	No data were excluded from any analyses.
Replication	All experiments were verified at least two independent replicates. All attempts at replication were successful.
Randomization	Randomization was not typically applied in enzyme assays and pathway reconstruction by one-pot and in E.coli, as samples were not allocated to groups.
Blinding	Blinding was not relevant to this study as samples were prepared and measured by the same researcher. In addition, these experiments

Blinding	required the insight of the researcher into the enzymes, genes and metabolites being analyzed.

Reporting for specific materials, systems and methods

We require information from authors about some types of materials, experimental systems and methods used in many studies. Here, indicate whether each material, system or method listed is relevant to your study. If you are not sure if a list item applies to your research, read the appropriate section before selecting a response.

Materials & experimental systems

n/a	Involved in the study
☒	☐ Antibodies
☒	☐ Eukaryotic cell lines
☒	☐ Palaeontology and archaeology
☒	☐ Animals and other organisms
☒	☐ Clinical data
☒	☐ Dual use research of concern

Methods

n/a	Involved in the study
☒	☐ ChIP-seq
☒	☐ Flow cytometry
☒	☐ MRI-based neuroimaging
